# Vascular effects on the BOLD response and the retinotopic mapping of hV4

**DOI:** 10.1371/journal.pone.0204388

**Published:** 2019-06-13

**Authors:** H. G. Boyd Taylor, A. M. Puckett, Z. J. Isherwood, M. M. Schira

**Affiliations:** 1 School of Psychology, University of Wollongong, Wollongong, NSW, Australia; 2 School of Psychology, University of Queensland, Brisbane, QLD, Australia; 3 Queensland Brain Institute, University of Queensland, Brisbane, QLD, Australia; Brigham and Women’s Faulkner Hospital, UNITED STATES

## Abstract

Despite general acceptance that the retinotopic organisation of human V4 (hV4) takes the form of a single, uninterrupted ventral hemifield, measured retinotopic maps of this visual area are often incomplete. Here, we test hypotheses that artefact from draining veins close to hV4 cause inverted BOLD responses that may serve to obscure a portion of the lower visual quarterfield—including the lower vertical meridian—in some hemispheres. We further test whether correcting such responses can restore the ‘missing’ retinotopic coverage in hV4. Subjects (N = 10) viewed bowtie, ring, drifting bar and full field flash stimuli. Functional EPIs were acquired over approximately 1.5h and analysed to reveal retinotopic maps of early visual cortex, including hV4. Normalised mean maps (which show the average EPI signal amplitude) were constructed by voxel-wise averaging of the EPI time course and used to locate venous eclipses, which can be identified by a decrease in the EPI signal caused by deoxygenated blood. Inverted responses are shown to cluster in these regions and correcting these responses improves maps of hV4 in some hemispheres, including restoring a complete hemifield map in one. A leftwards bias was found whereby 6/10 left hemisphere hV4 maps were incomplete, while this was the case in only 1/10 right hemisphere maps. Incomplete hV4 maps did not correspond with venous artefact in every instance, with incomplete maps being present in the absence of a venous eclipse and complete maps coexisting with a proximate venous eclipse. We also show that mean maps of upper surfaces (near the boundary between cortical grey matter and CSF) provide highly detailed maps of veins on the cortical surface. Results suggest that venous eclipses and inverted voxels can explain some incomplete hV4 maps, but cannot explain the remainder nor the leftwards bias in hV4 coverage reported here.

## Introduction

Human visual cortex is comprised of multiple orderly visual areas which are functionally and anatomically distinct from each other [1–4]. Many of these areas are organised according to the principle of retinotopy, which is to say they are topographically organised such that the spatial organisation of the retina and therefore the visual field, is mapped to the neurons of each area [5–7]. These retinotopic maps are highly preserved across individuals and species, hence the cortical organisation of visual areas is unlikely to be random, but an integral part of visuo-cortical function [8–10]. It is therefore important to determine the organisation and position of visual areas, as these factors are central to our understanding of visual information processing.

Functional magnetic resonance imaging (fMRI) is currently one of the most well-suited and popular tools for studying visual areas in human visual cortex, due to its safe and non-invasive nature; however, it is not without issue. The impact of veins on the measured blood-oxygen-level dependent (BOLD) response in fMRI has been emphasised as one such issue, both inside and outside the visual cortex [11–15]. Here, the strong paramagnetic effect of deoxyhaemoglobin (dHb) in veins changes the homogeneity of the adjacent local magnetic field, causing a reduction or loss of the fMRI signal in its vicinity [16, 17]. This hinders the usefulness of fMRI for studying some visual areas, as the BOLD signal can be obfuscated by venous artefact. For example, this artefact has been accused of contributing to the deterioration of fMRI measurements in human ventral occipital cortex, particularly in area V4—the organisation of which remains unsettled despite years of experimental investigation and debate [15, 18–24].

**Fig 1:**
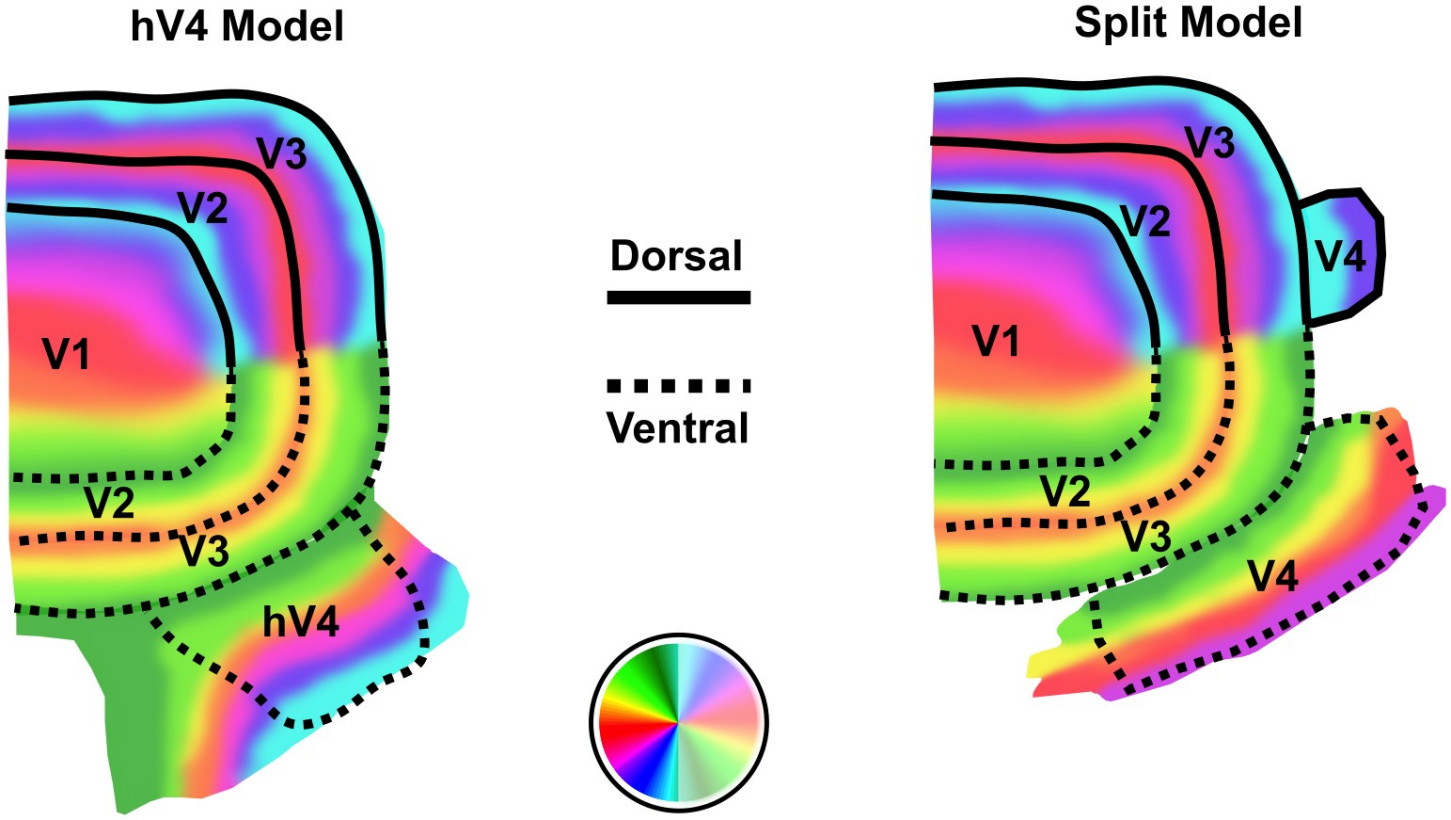
Diagrams of early visual cortex in humans showing the ‘hV4’ model (left) and the ‘Split’ model (right). According to the hV4 model, V4 contains a map of the entire contralateral visual hemifield in a continuous retinotopic map on the ventral surface, adjacent to ventral V3 (V3v). In this model, the lower boundary of hV4 is defined by a polar angle reversal at the lower vertical meridian on the ventral surface. The hV4 map illustrated here is based on descriptions provided in Winawer and Witthoft [28]. The ‘split’ model assumes the upper vertical meridian, contralateral horizontal meridian and approximately half of the lower visual quarterfield are represented on the ventral surface, while the remainder of the map—including the lower vertical meridian—is represented on the dorsal surface. The V4 map illustrated here is based on V4d and V4v retinotopic maps defined in Hansen and colleagues [21].

The most significant debate around V4 organisation in humans arguably centers around the observance of ‘incomplete’ maps on the ventral surface, which indicate that human V4 follows the same non-symmetric dorsal/ventral split seen in macaque V4 [15, 18–24]. However, the evidence for a dorsal component of V4 in humans is limited [21, 23, 25]. Rather, these incomplete maps are likely a result of the proximity of V4 to the Transverse Sinuses (TSs), a pair of large veins that drain dHb from the back of the head [15, 26]. In 2010, Winawer and colleagues [15] demonsrated how surface veins, in particular the TS, can be a source of measurement insufficiency affecting human V4 maps, especially on the ventral surface. Veins are known to cause a lower mean intensity of voxels in their proximity due to the high concentrations of dHb present within them [11–15]. This phenomenon has been called the ‘venous eclipse’ and it offers an elegant explanation for incomplete V4 maps, as the TS is anatomically close to this visual area [15]. Hence, it is likely that human V4 is organised in a single, continuous hemifield adjacent to ventral V3 [24] (hereafter called ‘hV4’, in accordance with accepted nomenclature for this model) and that incomplete maps are due to insufficiencies in measurement rather than being genuine portrayals of the existing retinotopic map [15, 27, 28]. A comparison of these two models is shown in Fig 1.

According to Winawer and colleagues [15], if artefact is near enough to the lower hV4 boundary, the measured map of hV4 will be incomplete due to signal dropout and, importantly, abnormally long delays in the BOLD response. Voxels exhibiting such time courses were reported not only to be significantly delayed, but even appeared counterphase to the expected response. That is to say, some delayed responses peaked 180° after the expected BOLD delay of 6s, thus peaking between stimulus cycles rather than in tandem with stimulation [29].

Counterphase delays in the measured BOLD response along the lower hV4 boundary such as those proposed by Winawer and colleagues [15] are one interpretation of time courses disrupted by venous artefact, however another explanation exists. As the BOLD signal is driven by changes in oxygen levels in blood [17, 30], with oxygenated blood producing positive BOLD responses (PBRs), veins draining deoxygenated blood would theoretically result in a decrease in the mean intensity of nearby voxels. Therefore, the haemodynamic response of voxels disturbed by venous artefact may be more accurately characterised as in phase but ‘inverted’, as was proposed by Puckett and colleagues [31], rather than delayed or counterphase.

To examine this, Puckett and colleagues [31] correlated voxel responses to a full field control stimulus with a reference haemodynamic waveform. Ordinarily, the BOLD response is coupled with neural firing thus, the simultaneous stimulation of the whole visual field should result in all the voxels within the range of the stimulus exhibiting PBRs and therefore produce smooth, orderly visual field maps [15, 31]. However, this is not always the case. Puckett and colleagues demonstrated that many voxels lying within both the boundary of simulation and the region of the venous eclipse exhibit an inverted response (that is, the response decreased with stimulation), rather than a delayed response. Furthermore, it was shown that the corresponding regions of polar angle maps was disrupted rather than smooth and orderly as would be expected based on the principle of retinotopic mapping [31]. These authors further showed that in V1, V2 and V3, time courses of voxels exhibiting inverted responses could be successfully corrected and that doing so restored the order of the previously disturbed visual field maps. Whilst the same procedure was not applied to inverted voxels in hV4, this area was shown to contain a significantly higher number of inverted voxels compared with other areas [31]. This suggests that correcting inverted voxels in hV4 may enable a more accurate measure of its response and visual field map in the region of the venous eclipse than has previously been possible.

Here, we aim to investigate the consistency of the venous eclipse and inverted voxel hypotheses proposed by Winawer and colleagues [15] and Puckett and colleagues [31], with relation to retinotopic maps of hV4. Based on the V4 literature in humans, we hypothesised that we would identify complete hV4 maps on the ventral surface of some hemispheres and incomplete maps in others. Furthermore, when an incomplete map was identified we hypothesised that a cause should be found, namely that a venous eclipse would also be present on or near the lower boundary of hV4, consistent with the hypothesis of Winawer and colleagues [15]. Additionally, it was predicted that in the region of the venous eclipse, voxels would present with on-time but inverted BOLD responses, as proposed by Puckett and colleagues [31]. Correcting these responses was predicted to result in the subsequent restoration of hV4 maps where they were affected by inverted voxels, potentially allowing a complete hemifield map to be identified where an incomplete map was initially measured. We further expected that any anomalous voxel responses, that is inverted, delayed or random responses, should be identifiable using a full field flash stimulus, as this should serve as a control that will distinguish voxels that fail to exhibit the expected BOLD response.

## Materials and methods

### Subjects

11 healthy subjects (5 female), aged 21-26 years with normal or corrected-to-normal vision participated in the study. Data from one subject was excluded due to excessive noise present in hV4 visual field maps. As such, the data presented here are from the remaining 10 subjects. The experiment was conducted with the understanding and written consent of each subject and was approved by the University of New South Wales Human Research Advisory Panel (HC14262).

### Visual field mapping

Travelling wave stimuli in the form of rotating bowties and expanding rings were used, adhering to the methods described in previous work [32, 33]. Stimuli were composed of a flickering chequerboard pattern under an extended grey fixation grid, with colours randomly changing every 0.25ms. A 3x3 pixel fixation dot (0.04°) was present in the centre of the screen, which changed colour every 3-8s, with subjects being instructed to press and hold a button whenever this dot was red. The bowties were presented for 15 cycles per scan and the rings for 12.

**Fig 2:**
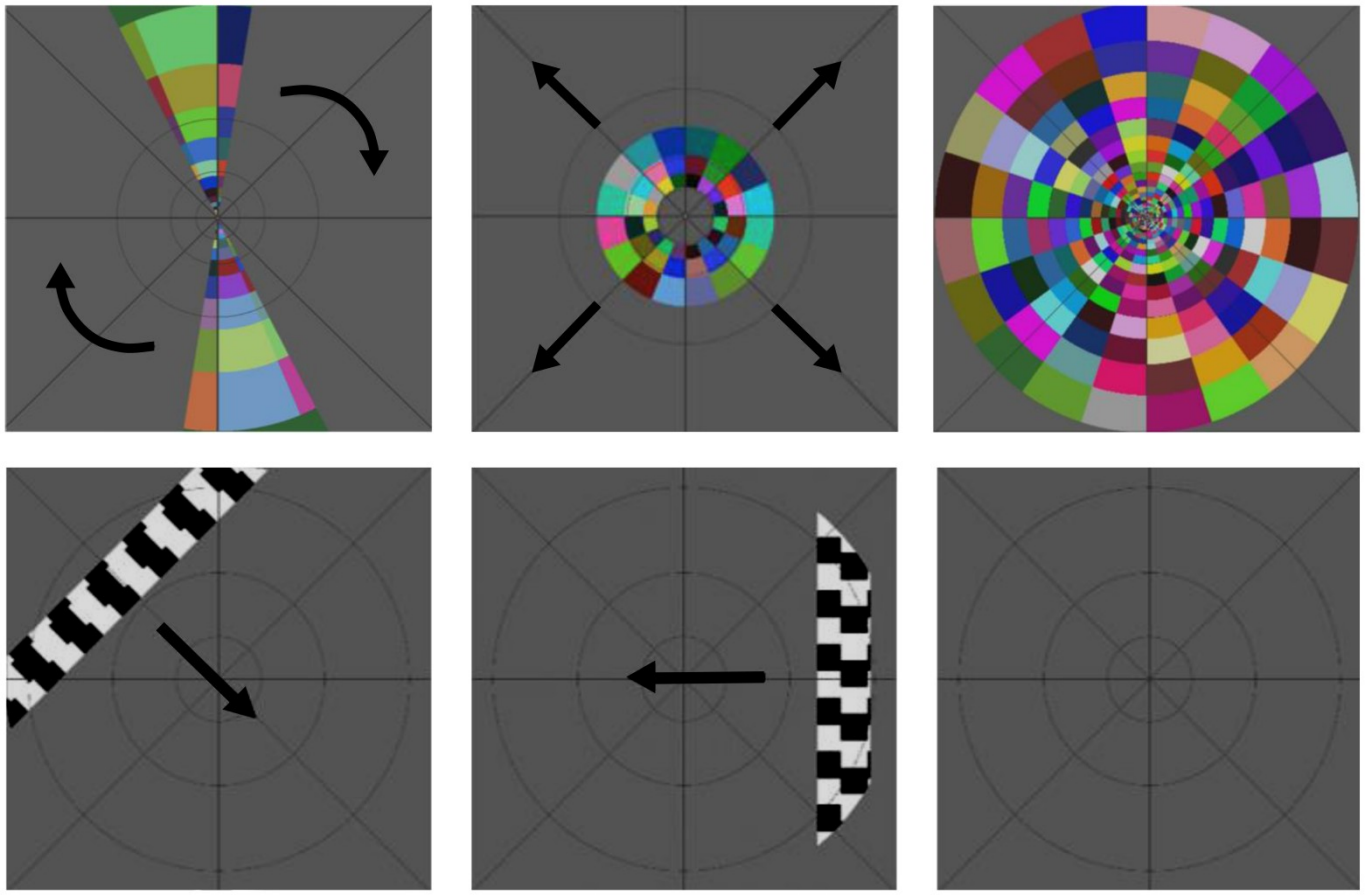
Top Row: Clockwise rotating bowtie, expanding rings and full field flash stimuli. Bottom Row: Drifting bar stimulus at three different time points, showing two of the eight bar directions and a blank luminance block. Arrows indicate the direction of movement.

Two further types of stimuli were used to enable additional analyses. The first of these was a full field flash which was composed of the same flickering chequerboard pattern and fixation grid as the bowties and rings. It was presented for 12 cycles per scan, where each cycle was comprised of an ON period of 4s and an OFF period of 16s. The other stimulus was a drifting bar, adapted from the same stimulus used by Dumoulin and Wandell [34], with the addition of a grey fixation grid [33]. The bar was composed of two black and white chequered bars, one inside the other, coasting in opposite directions. The bar was presented at four orientations (0°, 45°, 90° and 115°) and travelled in the two directions perpendicular to each, for a total of eight directions. Each bar sweep took 40s and there were four periods of a blank luminance block, each lasting 20s, inserted between every second sweep (Fig 2).

### Stimulus presentation

Stimuli were generated using MATLAB R2012a and Psychophysics Toolbox [35, 36] and displayed to participants on a 19” monitor with a 1024 × 768mm resolution. Participants viewed this monitor via a mirror attached to the head coil. The stimuli subtended a visual display spanning 5.5° eccentricity (or 11° of visual angle), with a viewing distance of 1.5m. Total stimuli presentation and scan time per subject varied, with two subjects viewing only one presentation of each stimulus, resulting in a viewing time of approximately 24m and the remaining eight subjects viewing each stimulus up to four times, resulting in total viewing time of 1.5-2h. For five of these subjects, data collection was split across two scanning sessions, one of 25m and the other 1h; the three remaining subjects were scanned in one 2h session.

### Data acquisition

Functional EPI data was collected using a Philips 3T Achieva TX Magnetic Resonance Scanner fitted with Quasar Dual gradients and a 32-channel head coil. 32 oblique, ascending coronal slices covering the occipital pole and ventral occipital cortex were acquired at a voxel resolution of 1.5mm^2^, a 128 × 128 matrix and a field of view (FOV) of 192mm. Images were acquired with a repetition time of 2s, echo time of 25ms and acceleration (SENSE) factor of 2. Higher resolution T1 anatomical images were collected at a resolution of 0.75mm^3^, using a 3D magnetisation-prepared rapid acquisition with gradient echo (MPRAGE) protocol with a matrix size of a 340 × 340, a FOV of 256mm and a TR of 6s. The number of volumes collected varied by stimulus. Bowties, rings and full field scans had some initial volumes discarded to compensate for the initial pulse of the scanner and enable the BOLD response to reach baseline. Details of volumes are as follows—Bowties: 186 volumes, discarded first six; Rings: 174 volumes, discarded first six; Full field: 124 volumes, discarded first four. Zero volumes were discarded from the bars as this stimulus began with an extended baseline (blank luminance block and fixation grid) of 20s. A venogram from Subject 4 was collected using the same parameters as the T1 anatomy scans.

### Cortical surface reconstructions

The cortical grey/white boundary of seven subjects was segmented automatically using FreeSurfer [37, 38] and then manually corrected using ITKGray [39]. For three subjects, segmentations were done entirely manually using ITKGray. Segmentations were installed in mrVISTA and 3D cortical surface reconstructions of the left and right hemispheres were created and displayed using mrMESH, which was also used to visualise fMRI data on the cortical surface (Stanford University, Stanford, CA; http://white.stanford.edu/software/). For Subject 4, the venous anatomy, including the Transverse Sinuses, Superior Sagittal Sinus, Straight Sinus and the confluence of sinuses, were manually segmented from the venogram using ITKGray.

### Preprocessing

Preprocessing of EPI images was performed using SPM8 (SPM software package, Wellcome Department, London, UK; http://www.fil.ion.ucl.ac.uk/spm/). Data was motion corrected using a rigid body transform and 7th degree B-spline interpolation. Images were slice scan time corrected using the first image as the reference slice and resliced into the space of the first image.

### Population receptive field (pRF) modelling

Bowtie, ring and bar stimuli were analysed using a population receptive field (pRF) method, whereby a parameterised model of the underlying neuronal response is used to calculate the predicted BOLD response. Essentially, this is a special adaptation of a linear regression, or the General Linear Model (GLM)—a standard technique in fMRI analysis [40]. The goodness-of-fit of this response is estimated using the residual sum of squares (RSS), which is minimised in a series of two-stage, coarse-to-fine searches until the optimum pRF parameters (position and size) are found. Further detail on this analysis can be found in Dumoulin and Wandell [34].

### Fast Fourier transform and correlation analyses

A fast Fourier transform (FFT) analysis was performed on the bowtie, ring and full field stimuli using inbuilt mrVISTA functions. Mean time courses from the full field stimulus were additionally analysed using a correlation analysis between the empirical waveform and a reference waveform, which was created by convolving the stimulus timing with a canonical model of the haemodynamic response function (HRF) using the WAV model of AFNI’s 3dDeconvolve [41]. MATLAB was used to estimate the correlation between voxel time courses and the reference waveform and voxels responding with ‘positive’ and ‘inverted’ HRFs were identified by the sign of their correlation value.

### Data integration across cortical depth

Data were restricted to the 3mm of voxels above the grey/white boundary (as defined by the segmentation) and averaged depth-wise to create normalised mean maps, correlation maps and retinotopic maps.

### Mean maps

Depth-integrated mean maps (which show the average EPI signal amplitude or EPI brightness) were constructed by voxel-wise averaging of the EPI time course within the grey matter and normalised by dividing the value of each voxel by the value of the voxel with the maximum intensity. The normalised mean maps were then used to identify venous eclipses, which have a lower intensity due to the higher concentration of dHb in veins.

Regions of interest (ROIs) were hand drawn around any venous eclipse that was identified and subsequently projected onto the surface displaying the correlation analysis data to check whether voxels with inverted responses clustered in the region of the venous eclipse. In determining whether a venous eclipse was present, we considered the shape and location of intensity drops, as venous eclipses are known to correspond with the anatomical locations of the Superior Sagittal Sinus and Transverse Sinuses and resemble the elongated shadow of a blood vessel.

As it has not been clearly established in the literature the extent to which artefact from the venous eclipse can spread, or the nature of the impact it may have on nearby voxels, we took a conservative approach toward classifying venous eclipses as having the ability to impact the lower hV4 boundary. As such, we considered the venous eclipse to have the ability to impact the visual field coverage of hV4 if it was present close to the hV4 map (images of all normalised mean intensity and polar angle maps are included as figures in the Supplementary Materials (S1 File)).

### Defining visual areas V1, V2, V3 and hV4

All visual areas were defined on the mrVista surface meshes using depth-integrated polar angle retinotopic maps as measured by the pRF modelling, with reference to the retinotopic maps generated by the FFT analysis. These two sets of maps matched up extremely well for V1, V2 and V3. The retinotopic map of hV4 was defined by a phase reversal in the polar angle maps at the lower vertical meridian or close to it if it was not present, following the ventral hemifield model [19, 24, 28]. The ‘inverted U’ in polar angle maps demarcating the hV4/VO1 boundary, expected eccentricity reversals between hV4 and VO1 in eccentricity maps, and the location of the ptCos sulcus were also used in guiding the definition of hV4 boundaries [28, 42].

It is well documented that voxels in regions directly outside the outermost eccentricity of the visual stimulus exhibit a ‘Negative BOLD Response’ (NBR) [43–45]. To ensure these voxels were excluded from analyses, visual area ROIs were cropped at the periphery of the mapped visual field, prior to the occurrence of NBRs. It is important here to discriminate NBRs, which typically signify a reduction in the neural response just outside the stimulated region of the visual field [43–45] from inverted voxels, which exhibit a negative correlation to visual stimulation in regions where the expected response is positive [31]. We defined ROIs of NBRs immediately outside V1 in all hemispheres.

### Identifying incomplete hV4 maps

**Fig 3:**
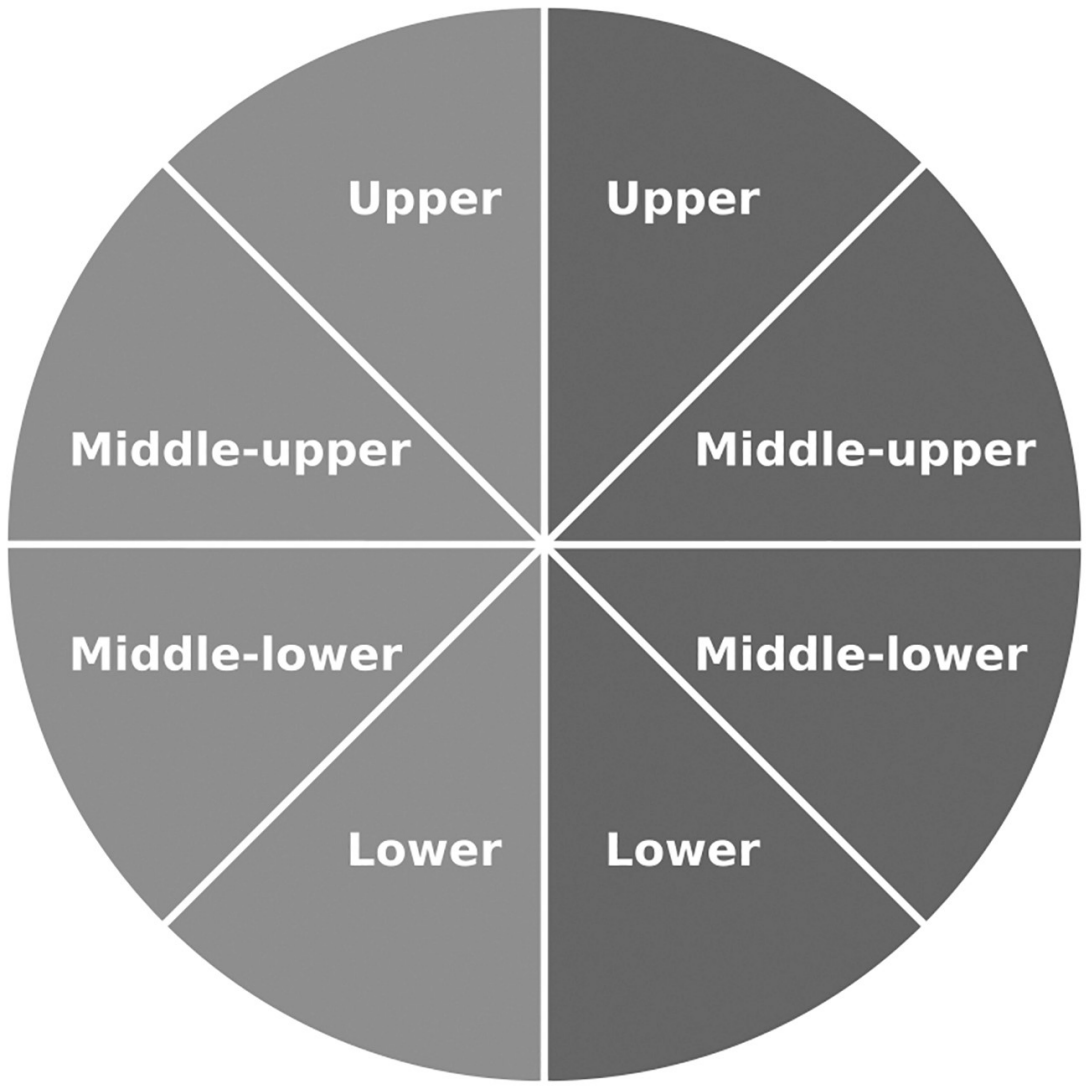
The division of hemifields into quadrants. The left hemifield is shown in light grey and the right hemifield in dark grey.

To classify hV4 maps as either ‘complete’ or ‘incomplete’, we divided each visual hemifield into quadrants which we refer to, beginning from the upper vertical meridian, as follows: the ‘upper’ quadrant, the ‘middle-upper’ quadrant, the ‘middle-lower’ quadrant and the ‘lower’ quadrant. The breakdown of these quadrants for the entire visual field is shown in Fig 3. From here, we measured the percentage of visual field coverage present in each hemifield quadrant based on a binarised version of a normalised pRF density plot, thresholded to include only regions of the visual field with a density of 0.8 and above. We used a cut-off of 20% coverage (rounded to the nearest whole number) as the criterion for ‘completeness’ within a quadrant. This cut-off was determined based on visual inspection of the coverage present in three very clear cases of ‘incomplete’ hV4 maps in the lower quadrant (LH; Subjects 4, 6 & 10), as for each of these percent coverage fell below 20% (min = 1.3%; max = 17.4%). Raw percent coverage of each quadrant and all subjects is presented in Tables 1-4 of the Supplementary Materials (S1 File).

### Correcting time courses and inverted voxel counts

Custom MATLAB scripts were used to flip the time courses of negatively correlated voxels in the bowtie, ring, bar and full field scans. Note that this procedure flips the time courses of all voxels exhibiting a negative correlation, i.e. NBRs and inverted voxels. Voxels with a positive correlation were left untouched. It is important to note that only negatively correlated voxels within the cropped visual area ROIs were included in analyses, unless otherwise stated, in order to prevent the presence of NBRs from influencing results pertaining only to inverted voxels. Corrected time courses were reanalysed using the pRF method, resulting in a set of original and corrected pRF models.

### Measuring the smoothness of hV4 maps

**Fig 4:**
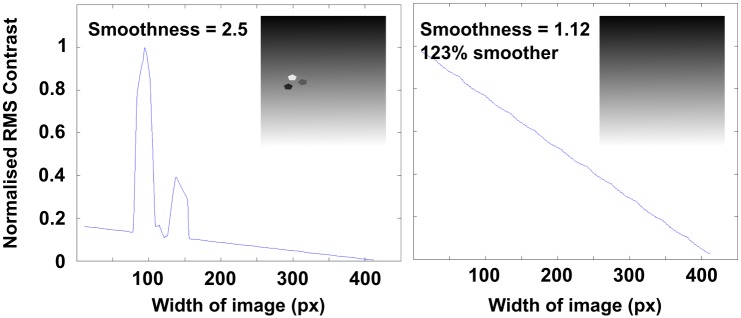
Model of a ‘non-smooth’ and ‘smooth’ retinotopic map, with RMS contrast plots and a corresponding ‘smoothness’ measure, based on the absolute gradient of the plotted line.

A fundamental principle of retinotopic mapping is that neighbouring voxels map neighbouring regions of the visual field. Theoretically, this should result in an orderly transition between polar angles, represented by coherent and smooth retinotopic maps. To test the smoothness of hV4 maps, an image of each hV4 map was taken as it was displayed on the surface mesh coloured with MATLAB’s ‘gray’ colour map. Maps were oriented so that polar angles were as horizontal as possible. These images were then cropped to include only the hV4 map, surrounded by a transparent background. These images were imported into MATLAB and normalised using the rbg2gray function. Normalised RMS contrast was then calculated and plotted for each column across the width of the image (px). We obtained a measure of the overall smoothness of hV4 maps by calculating the absolute gradient of the plotted line, where the closer the smoothness value is to one, the smoother the map is. To test this procedure, we modelled a ‘smooth’ hV4 map using a rectangle filled with a black-to-white horizontal gradient. Some random noise was added to represent disturbances in phase that would be expected as a result of inverted voxels and represent a ‘non-smooth’ map. Fig 4 shows these model maps and the resulting smoothness measures.

### Depth-dependent analysis

To investigate whether surface vessels have a visible effect on retinotopic maps at different cortical depths, for two subjects additional surfaces were generated at three depths using Caret v5.65 [46]. The deepest of these was generated at the grey/white boundary and expanded surfaces were created at depths of 1mm and 2.5mm from the grey/white boundary toward the cortical surface. As human visual cortex has an average thickness of 2-3mm [47], the 1mm surface is approximately located in the mid-depth of grey matter, whilst the 2.5mm surface is close to the grey matter surface, where the large draining veins reside. These surfaces were imported into the mrVISTA session using custom written MATLAB scripts, after which they were aligned to the anatomical data using mrVista. Average time courses for the bowtie, ring and full field scans were linearly interpolated from the mrVista volume space onto the depth-dependent surfaces using MATLAB. FFT analyses were performed on each interpolated time course and correlation analyses were computed for full field time courses; normalised mean maps of the full field data were also generated. Results were displayed on the surfaces using mrMesh. ROIs of V1, hV4 and venous eclipses were defined separately for this data, with reference to ROIs defined for the depth-integrated retinotopic maps.

### Statistical analyses

All additional statistical analyses were conducted using Jamovi 0.9 [48].

## Results

We aimed to evaluate 1) The consistency of the hypotheses that venous artefact obscures part of the lower quarterfield representation of the visual field in hV4, by resulting in either delayed [15] or inverted responses [31]; and 2) Whether it is possible to correct venous artefact in this area to more reliably measure visual field responses along the lower hV4 boundary. Our main findings were that venous eclipses did not always correspond with hV4 map coverage; the absence of venous artefact coincided with incomplete hV4 maps, whilst complete maps were found despite venous artefact being present. Improvements and deteriorations in hV4 maps were noted post correction of inverted voxels, but overall corrected maps were smoother than the original maps and contained increased visual field coverage in the lower quadrant of the left hemisphere. Note that while we include results from all contralateral hemifield quadrants in both the main paper and Supplementary Materials (S1 File), we focus our results largely on the lower quadrant as this is the region most often reported to be ‘missing’ from hV4 maps.

### hV4 visual field coverage

We identified visual field maps of hV4 in both cerebral hemispheres of all 10 subjects. In total there were 13 complete and 7 incomplete lower quadrants in the hV4 maps. The left hemisphere was disproportionately represented, with 6/10 lower quadrants being incomplete, compared to 1/10 in the right hemisphere. Incomplete coverage in the upper, middle upper and middle lower quadrants was relatively rare (7/60 quadrants). Table 1 presents a summary of the completeness of each hemifield quadrant, as well as the presence or absence of a venous eclipse near hV4.

**Table 1:** hV4 visual field coverage of each hemifield quadrant and venous eclipse presence in or near hV4.

	Upper	Middle upper	Middle lower	Lower	Venous eclipse
**Left Hemisphere**					
sub-01	Complete	Complete	Complete	Complete	Absent
sub-02*	Complete	Complete	Complete	Complete	**Present**
sub-03	Complete	Complete	Complete	**Incomplete**	**Present**
sub-04	**Incomplete**	Complete	Complete	**Incomplete**	**Present**
sub-05	Complete	Complete	Complete	**Incomplete**	**Present**
sub-06	Complete	Complete	Complete	**Incomplete**	**Present**
sub-08*	**Incomplete**	**Incomplete**	Complete	Complete	**Present**
sub-09	Complete	Complete	Complete	Complete	Absent
sub-10*	Complete	Complete	Complete	**Incomplete**	Absent
sub-11*	Complete	Complete	Complete	**Incomplete**	Absent
**Total I/P**	**2/10**	**1/10**	**0/10**	**6/10**	**6/10**
**Right Hemisphere**					
sub-01	Complete	Complete	Complete	Complete	Absent
sub-02	Complete	Complete	Complete	Complete	Absent
sub-03	**Incomplete**	Complete	Complete	Complete	Absent
sub-04	Complete	Complete	Complete	Complete	Absent
sub-05	**Incomplete**	**Incomplete**	**Incomplete**	**Incomplete**	**Present**
sub-06	Complete	Complete	Complete	Complete	Absent
sub-08*	Complete	Complete	Complete	Complete	**Present**
sub-09 *	Complete	Complete	Complete	Complete	**Present**
sub-10	Complete	Complete	Complete	Complete	Absent
sub-11	Complete	Complete	Complete	Complete	Absent
**Total I/P**	**2/10**	**1/10**	**1/10**	**1/10**	**3/10**

* Indicates incongruent pairings between the lower quadrant coverage and presence of a venous eclipse.

Note: This table does not include data from Subject 7 as this subject was excluded from analyses.

A 2x4x4 Repeated Measures ANOVA was conducted to test for differences in the percentage of visual field coverage between hemispheres, visual areas and quadrants. The assumption of sphericity was violated for visual area and the hemisphere x quadrant interaction therefore, results reported for those effects have been Greenhouse-Geisser corrected. Significant main effects were found for visual area (F(1.24,11.29) = 53.82, p < .001, *η*_p_^2^ = 0.857) and quadrant (F(3,27) = 12.41, p < .001, *η*_p_^2^ = 0.58). Significant interaction effects were present for hemisphere x quadrant (F(3,27) = 5.07, p = 0.006, *η*_p_^2^ = 0.36) and visual area x quadrant (F(3.37,30.31) = 11.61, p < 0.001, *η*_p_^2^ = 0.563).

Previous literature has shown that in hV4, a portion of the lower visual quarterfield, including the lower vertical meridian, is often missing from maps [15, 18–24, 27]. As hV4 is unique among early visual areas in terms of a consistent finding that it often appears to map less than a complete hemfield and in light of the main effect of visual area reported here, post hoc tests were conducted which showed that coverage in the lower quadrant of hV4 was significantly less than the lower quadrants of V1 (M_diff_ = 35.04%, t(27) = 10.34, p < .001), V2 (M_diff_ = 36.39%, t(27) = 10.74, p < .001) and V3 (M_diff_ = 33.81%, t(27) = 9.98, p < .001)—all Bonferroni adjusted for multiple comparisons. Further to this, it has been shown that the problem of incomplete hV4 maps is often worse in the left hemisphere [15]. As such, a paired samples t-test was conducted between the percent coverage of the lower quadrant in the left (M = 23.8%, SD = 18.4) and right (M = 51%, SD = 28.5) hemispheres. Results showed a significant difference was present, in favour of the right hemisphere having more coverage (M = 54.8%, SE = 5.8) than the left hemisphere (M = 23.8%, SE = 9) (t(9) = -2.81, p = 0.02).

### Venous eclipses

Using the normalised mean maps, we identified venous eclipses in 19 hemispheres, with 9 of these being on the ventral surface close to the measured hV4 maps. Of these 9, 6 were in the left hemisphere. There was no difference between the left and right hemispheres in terms of the total number of venous eclipses present.

We did not find a clear relationship between the presence of a venous eclipse near hV4 and the degree of visual field coverage represented. Whilst incomplete maps of hV4 did correspond with the presence of a venous eclipse in some hemispheres, we identified instances where an incomplete hV4 map was measured despite the absence of a venous eclipse and likewise instances of complete maps of hV4 in the presence of a venous eclipse; we refer to these as ‘incongruent’ pairings (denoted by * in Table 1).

### Correcting hV4 maps

In general correcting inverted voxels had a positive impact on retinotopic maps of hV4. On average, corrected left hemisphere maps were 9.2% smoother than original maps, while corrected right hemisphere maps were 7.7% smoother. Smoothness plots and measurements for all hV4 maps are included as figures in the Supplementary Materials (S1 File). Correcting inverted voxels reversed the classification of coverage from ‘incomplete’ to ‘complete’ in 3/6 lower quadrants (LH; sub-03, sub-05 and sub-10). A summary of quadrant completeness in hV4 after correcting inverted voxels is presented in Table 2.

**Table 2:** hV4 visual field coverage after correcting inverted voxels.

	Upper	Middle upper	Middle lower	Lower
**Left Hemisphere**				
sub-01	Complete	Complete	Complete	Complete
sub-02	Complete	Complete	Complete	Complete
sub-03	Complete	Complete	Complete	**Complete** *
sub-04	Incomplete	Complete	Complete	Incomplete
sub-05	Complete	Complete	Complete	**Complete** *
sub-06	Complete	Complete	Complete	Incomplete
sub-08	Incomplete	Incomplete	Complete	Complete
sub-09	Complete	Complete	Complete	Complete
sub-10	Complete	Complete	Complete	**Complete** *
sub-11	Complete	Complete	Complete	Incomplete
**Total Incomplete**	**2/10**	**1/10**	**0/10**	**3/10**
**Right Hemisphere**				
sub-01	Complete	Complete	Complete	Complete
sub-02	Complete	Complete	Complete	Complete
sub-03	Incomplete	Complete	Complete	Complete
sub-04	Complete	Complete	Complete	Complete
sub-05	Incomplete	Incomplete	Incomplete	Incomplete
sub-06	Complete	Complete	Complete	Complete
sub-08	Complete	Complete	Complete	Complete
sub-09	Complete	Complete	Complete	Complete
sub-10	Complete	Complete	Complete	Complete
sub-11	**Incomplete** *	Complete	Complete	Complete
**Total Incomplete**	**3/10**	**1/10**	**1/10**	**1/10**

Bold* indicates changes from the uncorrected maps.

Note: This table does not include data from Subject 7 as this subject was excluded from analyses.

**Fig 5:**
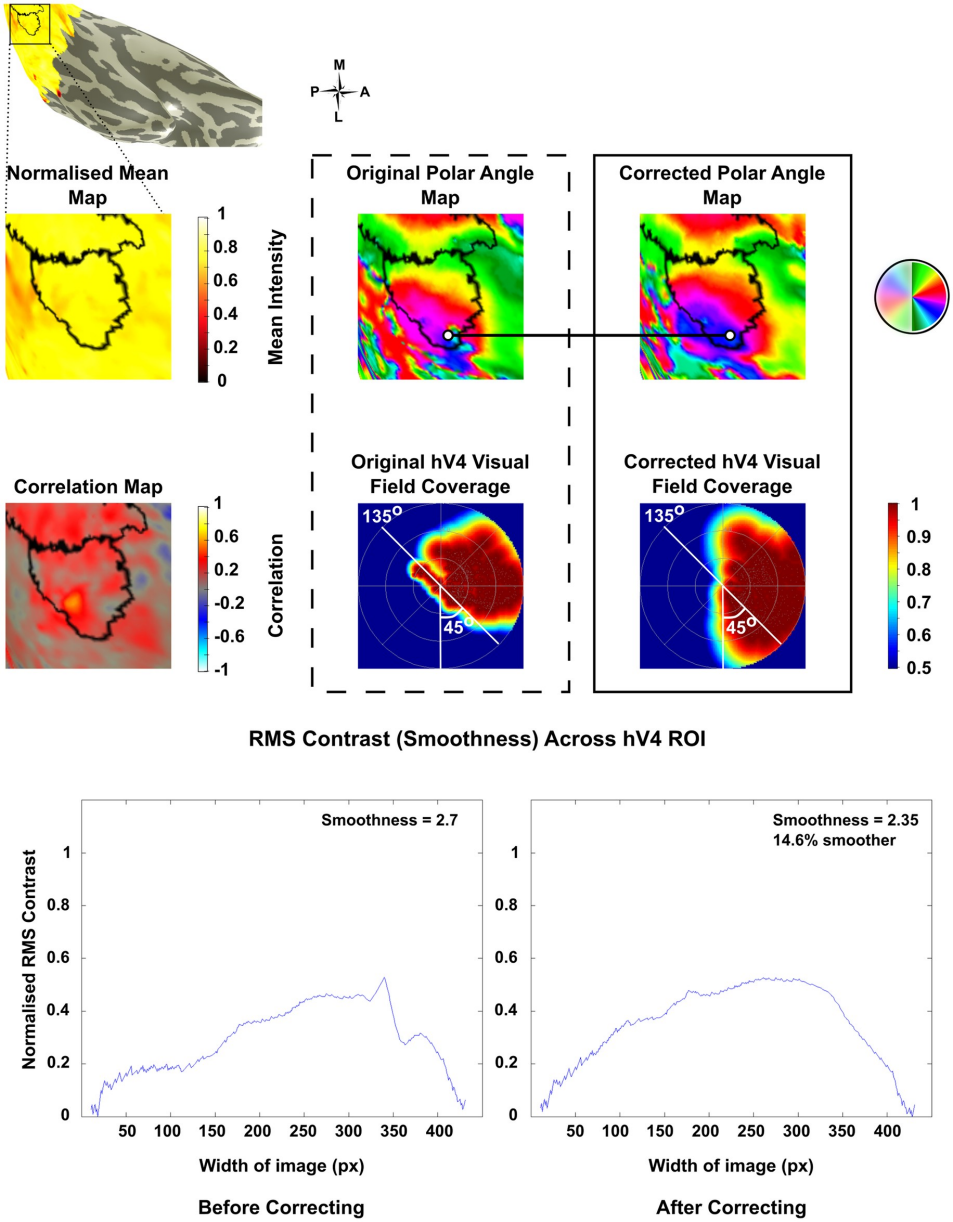
Restoration of coverage in the lower quadrant of hV4 was seen in the left hemisphere of Subject 10. The original phase map shows that coverage does not extend much beyond 45°. In the corrected pRF map, hV4 coverage extends further, almost reaching the lower vertical meridian. Normalised pRF density plots reflect the phase mapping and lower quadrant coverage in the original (11.05%) vs corrected map (86.07%). Smoothness also improved post correction. In addition to these changes, coverage of the ipsilateral hemifield at 135° in the original map is not present in the corrected map.

**Fig 6:**
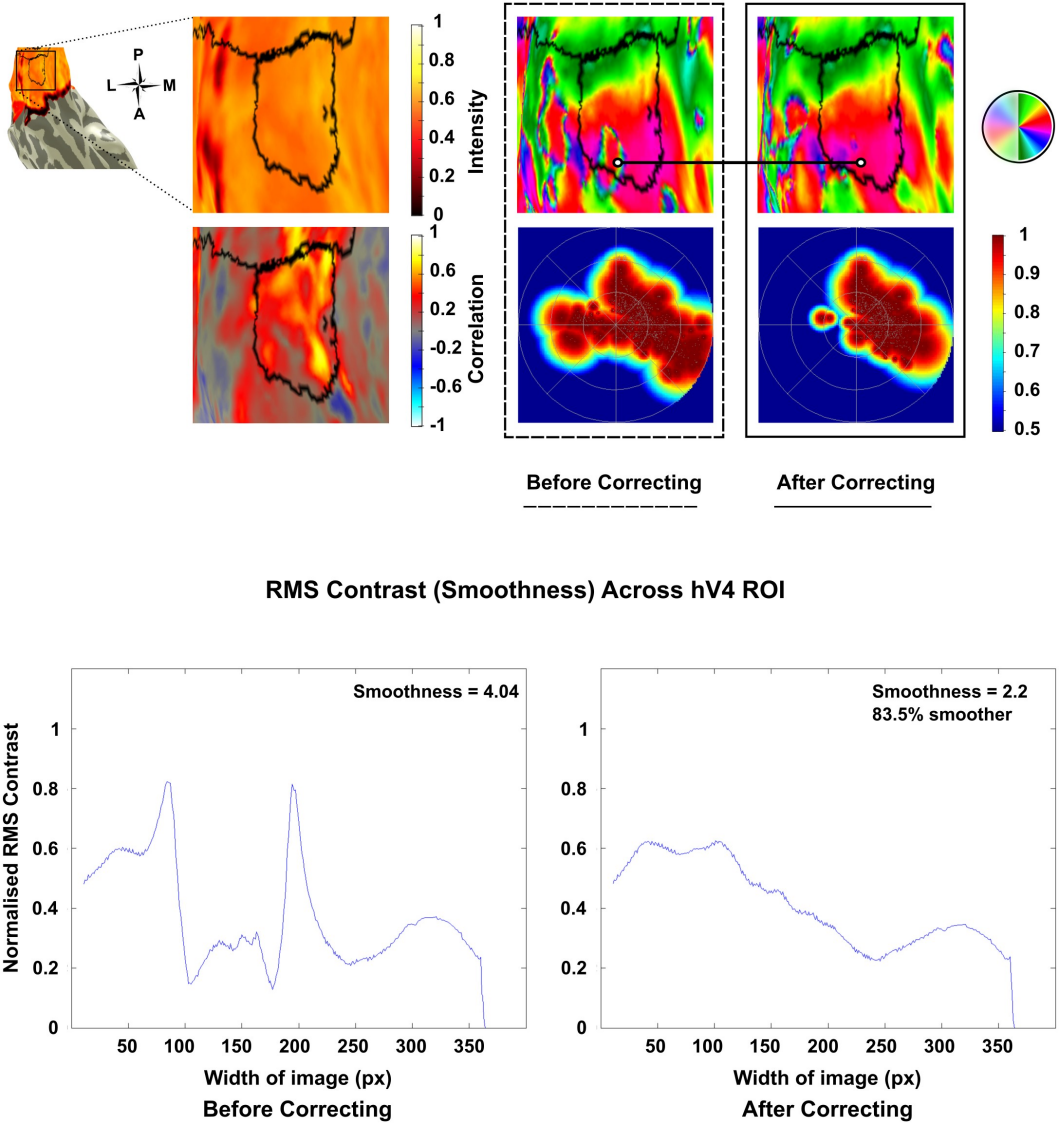
Improvements in the left hemisphere hV4 map of Subject 6 were noted post correction of inverted voxels. Initially a disturbed polar angle map was recorded along the lower boundary, which was restored after flipping inverted voxels. The normalised pRF denisty plot for the corrected map shows fewer voxels appearing to respond to the ipsilateral hemifield and the corrected map is 83.5% smoother, though still incomplete.

**Fig 7:**
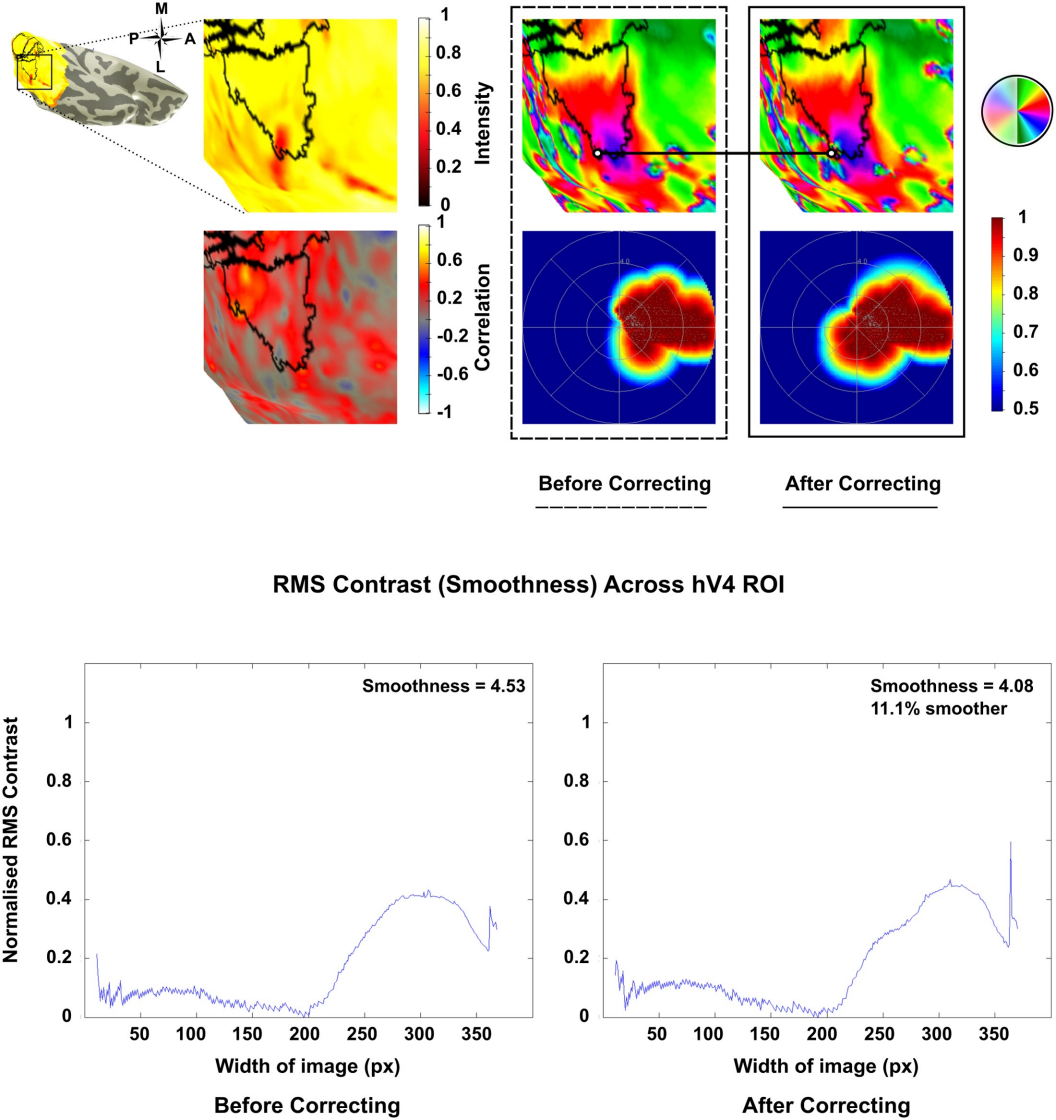
Coverage in the lower quadrant of Subject 3 (LH) increased from 19.1% to 25.8% post correction. Polar angle mapping shows minimal changes and improvements in map smoothness post correction are inconsequential. Regions of improvement are highlighted by the white circles.

**Fig 8:**
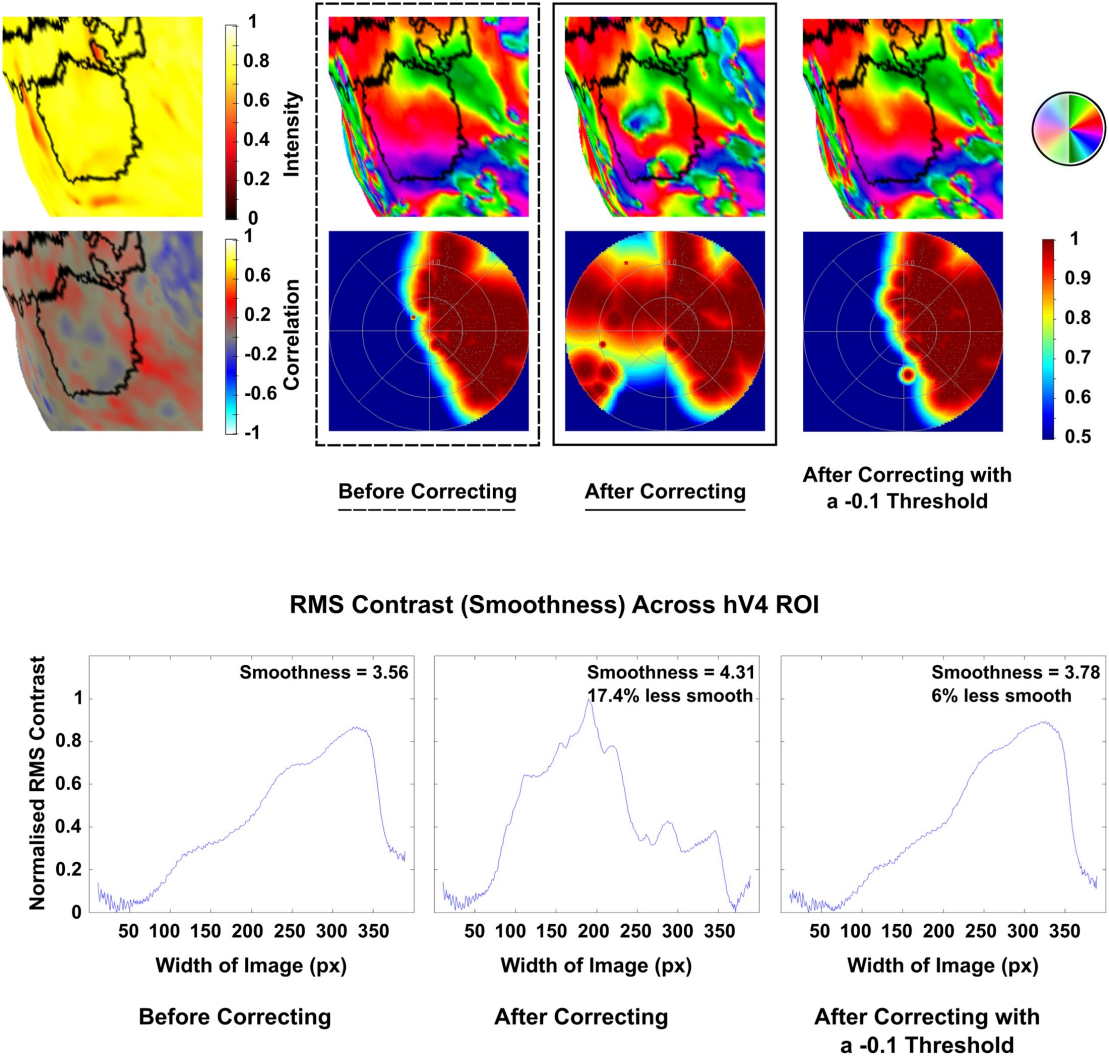
Deterioration of hV4 was present in the left hemisphere of Subject 2 post correction, where a large amount of ipsilateral coverage appeared in the pRF density plot and the polar angle map is considerably disturbed (17.4% less smooth after correction). However, these effects largely disappeared after correcting only inverted voxels with a correlation stronger than -0.1.

A dramatic improvement was seen post correction in the left hemisphere of Subject 10, which was restored from 11% coverage in the lower quadrant to 81.6% post correction (Fig 5). Other maps showed improvements in smoothness and phase mapping despite lower quadrants remaining incomplete in some instances (Figs 6 & 7). However despite these improvements a small number of maps showed a deterioration in smoothness and phase mapping post correction (Fig 8).

A paired samples t-test was conducted on the percent coverage of the lower quadrant of hV4 maps post correction to test if correcting inverted voxels affected the hemispheric bias noted for uncorrected data. The results showed that after correction no significant difference in coverage was present between the right (M = 55.9%, SE = 7.9) and left (M = 34.4%, SE = 8.33) hemispheres (t = -2.21, p = 0.054). Pre and post correction results for all hV4 polar angle maps, smoothness measures and percent quadrant coverage are presented in the Supplementary Materials (S1 File).

### Inverted voxels

**Fig 9:**
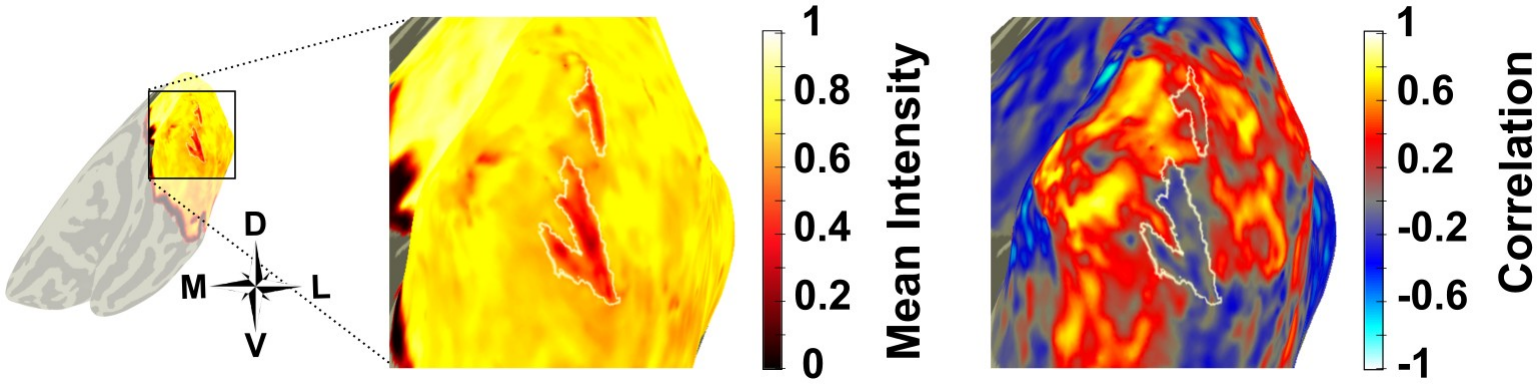
Inverted voxels tended to cluster in regions of the venous eclipse, as shown here in the right hemisphere of Subject 5 where the venous eclipse is defined in white on the normalised mean map. The correlation map shows a cluster of negatively correlated voxels in the same location however, these extend beyond the region the of venous eclipse where corresponding regions of the mean map do show a lower mean intensity compared the locations of positive voxels, though it is not as dramatic as the low mean intensity of the venous eclipse.

Inverted voxels were present in all visual areas examined and always, though not exclusively, clustered in regions corresponding with venous artefact. Because venous artefact is associated both with a drop in mean intensity and inverted responses [11–15, 31], a correlation analysis was conducted on the mean intensity and correlation values of voxels in combined left and right hemisphere V1-V4 ROIs for all subjects. There was a moderate relationship between these values (r = 0.25, p < .001, N = 215 001); a 2D histogram of mean intensity vs correlation is included in the Supplementary Materials (S1 File). Inverted voxels did not always strictly adhere to the region of the venous eclipse, with inverted voxel clusters extending into neighbouring regions (Fig 9; mean intensity and correlation maps for all subjects are included in the Supplementary Materials (S1 File)). Percentages of inverted voxels across visual areas and hemispheres are presented in Table 3.

**Table 3:** Normalised percentages of inverted voxels in visual areas V1–V4, averaged across subjects.

	V1		V2		V3		hV4	
Hemisphere	Left	Right	Left	Right	Left	Right	Left	Right
Total # Voxels	36 158	32 968	31 774	34 373	27 938	29 879	11 555	10 358
% Inverted	7.63	7.36	12.57	15.74	10.61	13.71	10.52	10.39

**Fig 10:**
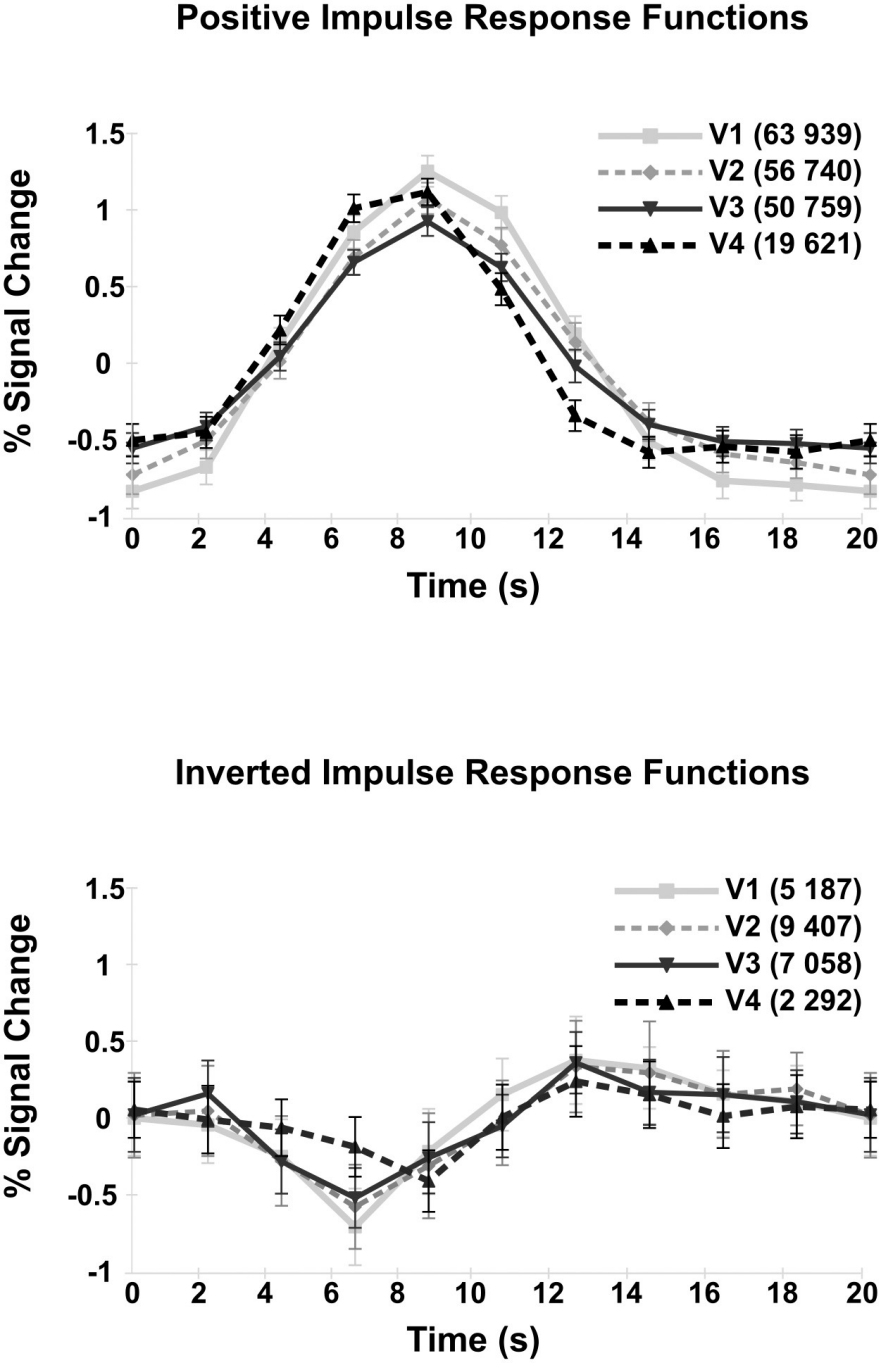
Positive and inverted IRFs for V1-hV4, averaged across subjects and hemispheres. Voxel counts are displayed in brackets. Error bars represent +-1 SEM.

Impulse response functions (IRFs) were calculated for the full field stimulus to examine time courses of positive vs inverted voxels (Fig 10). No differences between the left and right hemisphere IRFs were apparent, therefore data displayed here is collapsed across hemispheres. Voxels exhibiting an inverted BOLD response in hV4 have the lowest percent signal change of all areas; a lower percent signal change is also present in V1-V3 for inverted voxels compared to positive voxels. Positive responses did not vary across visual areas, exhibiting a consistent time-to-peak of 8s after stimulus onset. Inverted responses showed some slight variation, with a time-to-peak of 6s for V1-V3, while the time-to-peak for V4 was 8s.

### Inverted voxels vs. negative BOLD responses

Although our main hypotheses concerned hV4, an ancillary effect of performing the correction procedure on negatively correlated voxels outside this area was noted for visual areas V1, V2 and V3. Instead of corresponding to the venous eclipse, a large number of voxels exhibiting a negative correlation to the full field stimulus could be classified as Negative BOLD Responses (NBRs). These represent neuronal suppression and are usually located just outside (and in some cases just within) the stimulated region [44, 45, 49].

**Fig 11:**
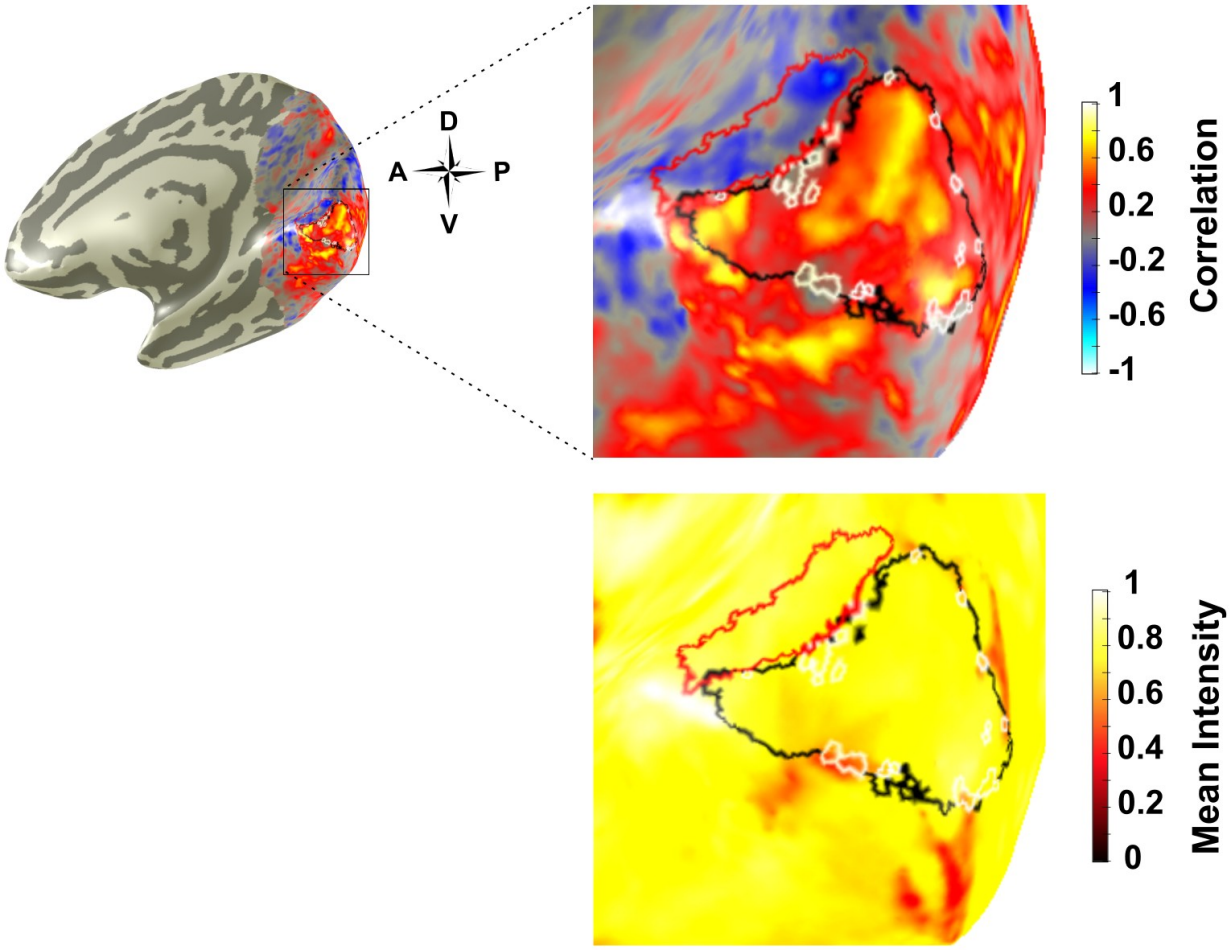
Right hemisphere correlation and mean intensity map from Subject 5 showing ROIs of NBRs outside V1 (red), V1 (black) and inverted voxels within V1 (white). Note the stronger negative correlation values of the NBRs vs. the inverted voxels in the correlation map, in addition to the stronger mean intensity values of the NBRs vs. the inverted voxels in the mean intensity map.

**Fig 12:**
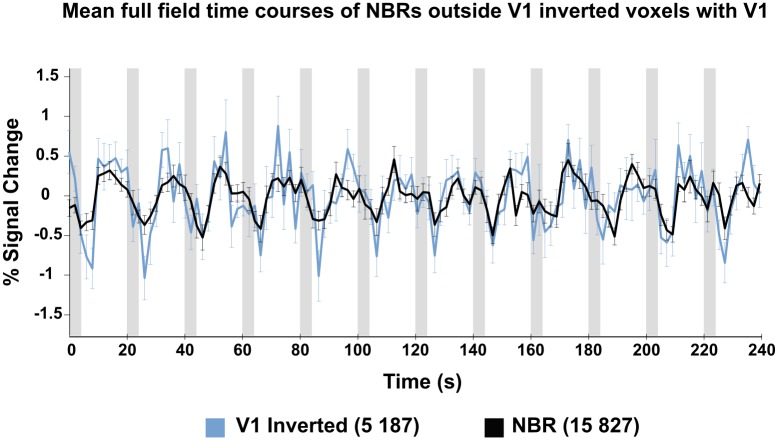
Average full field time course from inverted V1 voxels and NBRs, averaged across the left and right hemispheres of all subjects. The time course of inverted voxels is noisier and shows stronger variability and percent signal change compared to the NBR time course. Error bars represent +-1 SEM. Grey blocks indicate stimulus ON periods.

We consistently found NBRs located just outside the eccentricity range of visual stimulation (i.e. 5.5°eccentricity) for V1-V3. These voxels corresponded to correlation values that were almost as strong in the negative direction as positively correlated voxels that were within the range of visual stimulation (Fig 11). NBRs outside V1 had a higher normalised mean intensity compared to inverted voxels within V1 (0.73, SE = 0.01 vs 0.54, SE = 0.03, averaged across subjects). We also find time courses of inverted voxels to be noisier than NBRs, showing stronger percent signal change and higher variability (Fig 12).

**Fig 13:**
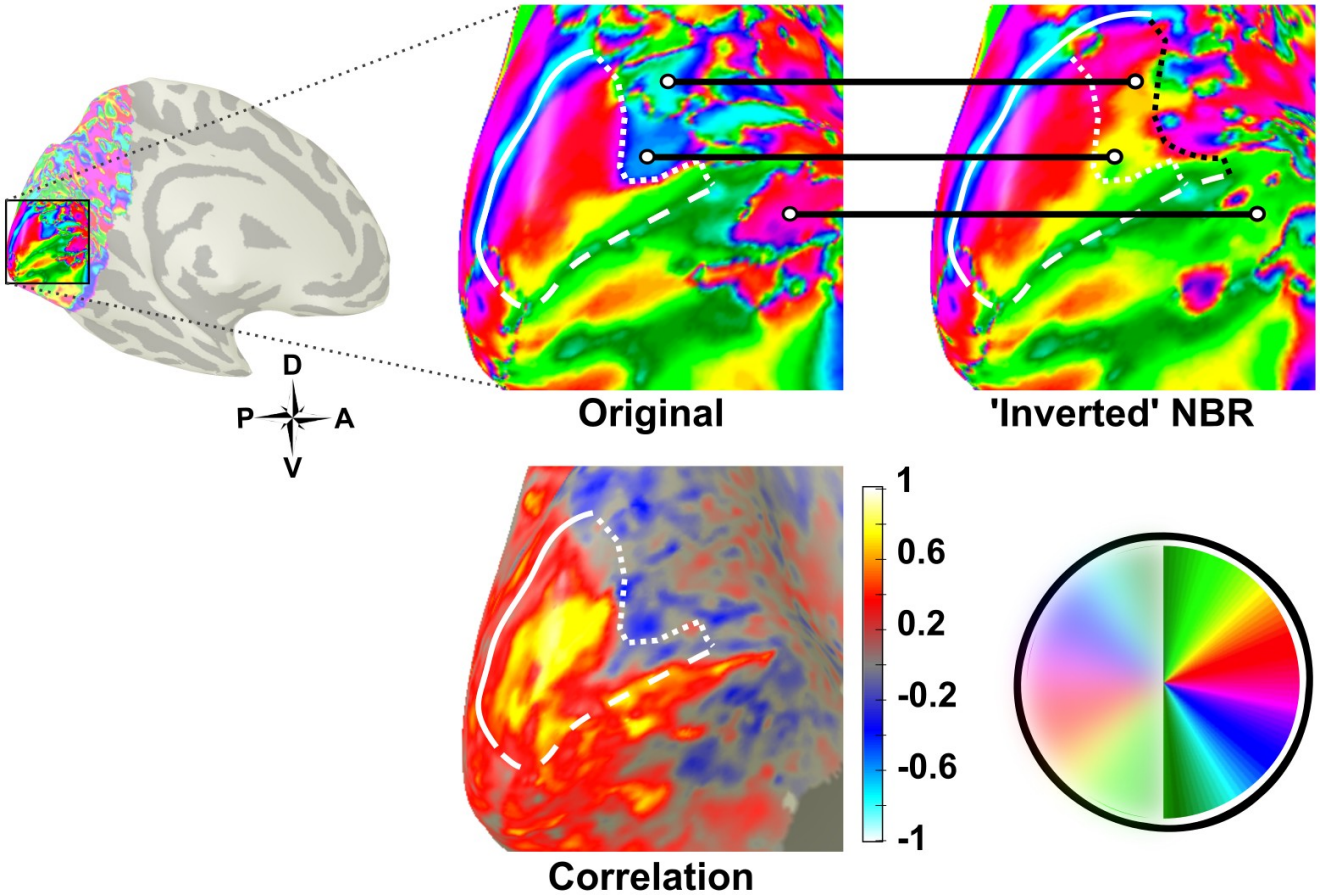
Retinotopic map of V1 (white) in the left hemisphere of Subject 4 before and after flipping negatively correlated time courses. The white circles indicate the same regions in the ‘original’ and ‘corrected’ data. The extension to the V1 map is indicated by the black dotted line in the ‘inverted NBR’ image.

Large improvements in polar angle phase maps were observed at the periphery of V1-V3 after flipping the time courses of NBRs. This was particularly noticable in V1, where large clusters of NBRs were often present in regions immediately adjacent to the region of cortex being stimulated. Here the correction procedure (i.e. flipping of all negatively correlated time courses) had a striking effect, where coverage of the visual field map was extended beyond the furthest eccentricity of the stimulus (Fig 13). This demonstrates the potential to retinotopically map visual cortex beyond the often limited eccentricity range of MR visual display systems.

### Depth-dependent effects of venous artefact on retinotopic maps

**Fig 14:**
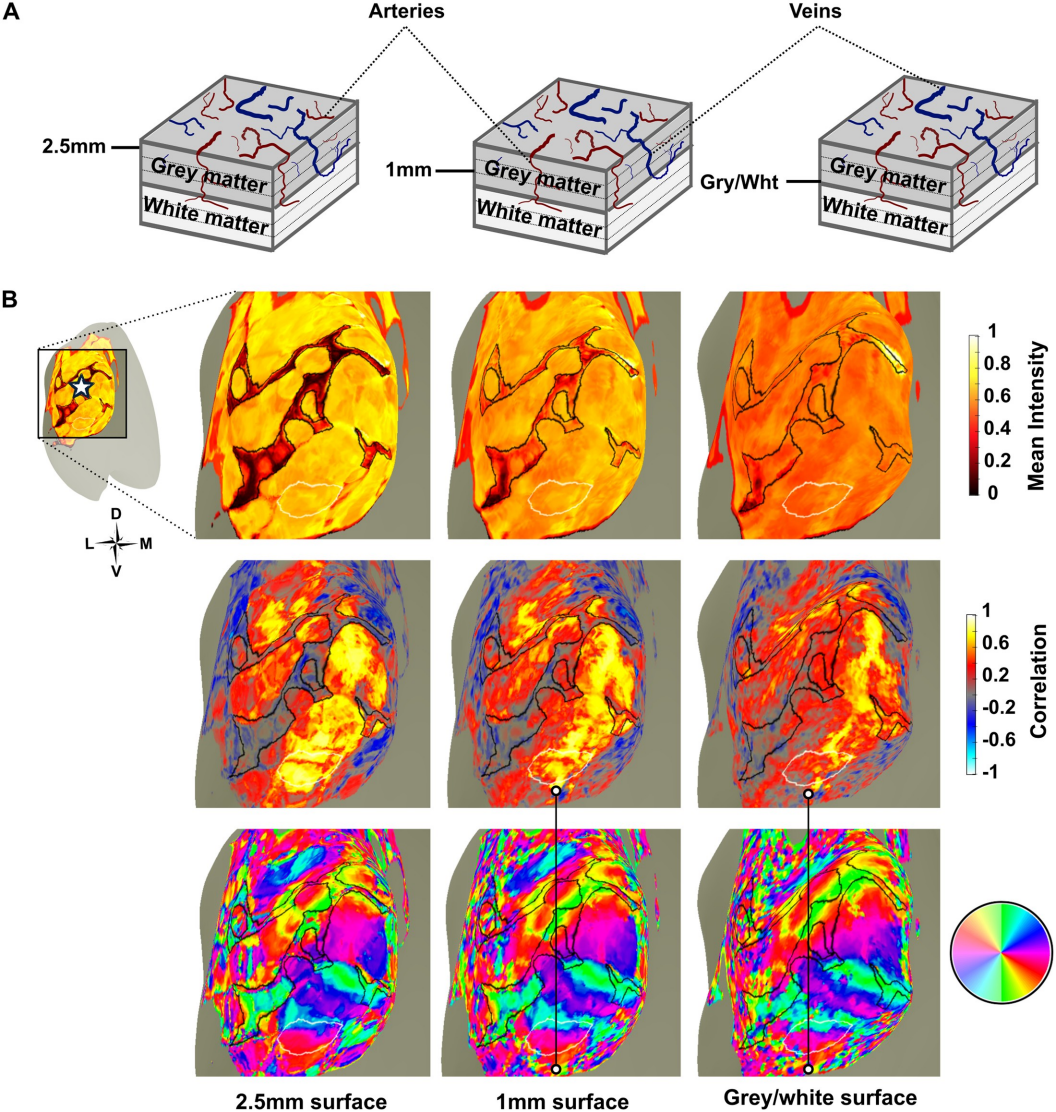
Depth-dependent effects of the venous eclipse in the left hemisphere of Subject 6. A) Schematic of layers of the cerebral cortex depicting cortical arteries (red) and veins (blue). These dive through the grey matter prior to turning at 90° at the white matter and thinning into branches [50]. B) Depth-dependent surfaces at 2.5mm, 1mm and the grey/white boundary showing the venous eclipse (outlined in black) and hV4 (outlined in white). The star in the top left insert represents the occipital pole. Mean intensity maps (top row) show the venous eclipse weakening with depth; a corresponding reduction in the number of inverted voxels within the venous eclipse is evident in the correlation maps (middle row). Polar angle maps (bottom row) show an incomplete map of hV4, which does not change across depth (note the ‘speckles’ indicated by the white dots in the second and third columns highlight what could be the lower vertical meridian of the hV4 map however, as the correlation map shows multiple inverted voxels in this region, it is difficult to trust the responses of these voxels).

To extend our analysis of the effect of venous artefact on retinotopic maps, we performed a post hoc investigation of its impact across cortical depth. Cortical surfaces at three depths (grey/white, 1mm and 2.5mm above the grey/white boundary) were generated for two subjects. Normalised mean maps for all 2.5mm surfaces showed clearly and in detail what appear to be venous eclipses caused by the Superior Saggital Sinus (SSS) and Transverse Sinuses (TSs). This is in contrast to the depth-integrated mean maps (shown in Figs 4 & 6), where averaging data across 3mm of grey matter may have had the effect of weakening the appearance of the venous eclipse—in some places to the extent that it disappears entirely. In the depth-dependent maps, venous eclipses increasingly weaken in appearance with depth, which could be clearly seen in the 1mm and grey/white surfaces (Fig 14).

A reduction in the mean intensity of all voxels was apparent with decreasing cortical depth. This likely reflects the fact that the BOLD signal is generally stronger in layers of grey matter closer to the pial surface [51]. Strikingly, voxels in regions surrounding the venous eclipse show a high mean intensity despite being immediately adjacent to venous artefact. This is particularly evident at the more superficial depths.

**Fig 15:**
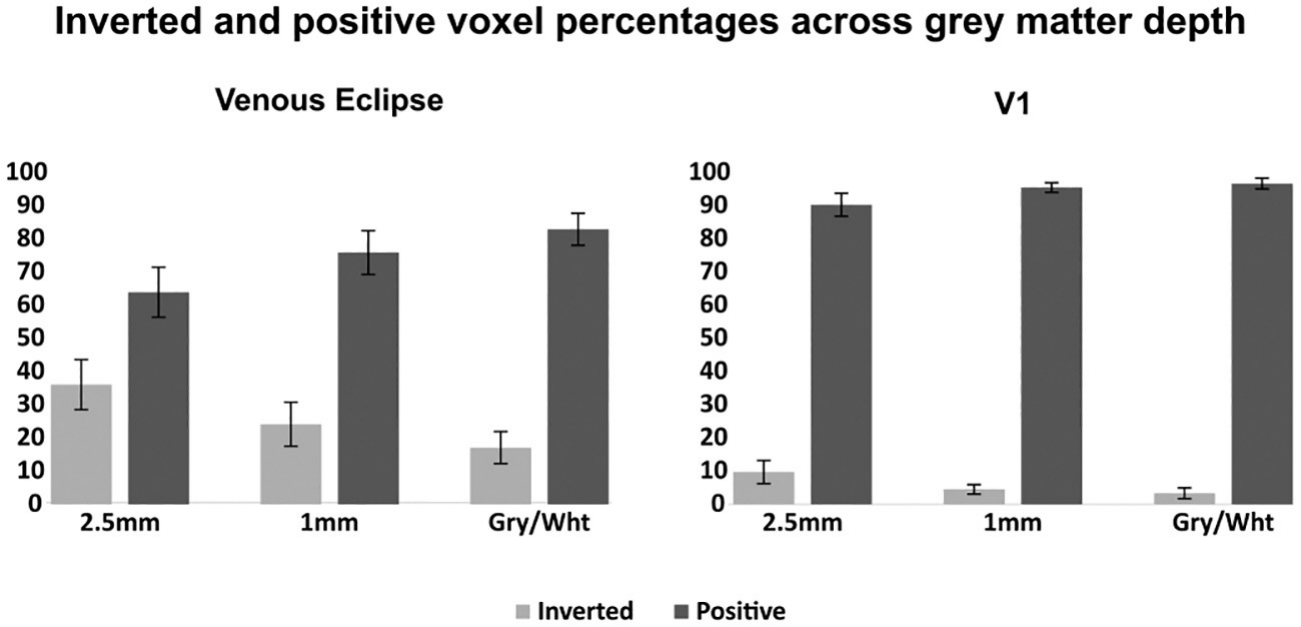
Average percentage of inverted and positive voxels in the venous eclipse and V1 for the left and right hemispheres of Subjects 5 and 6. A reduction in the mean percentages of inverted voxels within both regions is apparent with progression away from the cortical surface and toward the grey/white boundary. The venous eclipse has higher percentages of inverted voxels at every surface level compared to V1. Error bars represent +-1 SD.

Compared to the depth-integrated data, inverted voxels are more tightly restricted to locations within the venous eclipse and not outside it; this is especially true in the 2.5mm surfaces (see Fig 14B, rows 1 & 2). This indicates that whilst voxels immediately beneath surface vessels respond with abnormal time courses, voxels directly adjacent to, but outside this region, behave with expected, positive responses. This expected response is reflected in the mean intensity of these voxels, which is comparable to those which are not near venous artefact. Inverted voxels are present at a higher concentration within the venous eclipse compared to outside it and decrease in frequency with grey matter depth both inside and outside the venous eclipse (Fig 15). Other, more sparsely distributed voxels exhibiting negative correlations appear in regions distant from the venous eclipse in the 1mm and grey/white surfaces which unlike those lying within the venous eclipse, increase in frequency with depth.

## Discussion

Our primary aim was to evaluate the consistency of venous eclipses and inverted voxels as limitations to accurately measuring retinotopic maps of hV4 [15, 31]. We report results pertaining to hV4 maps, venous eclipses and inverted voxels, as examined in depth-integrated and depth-dependent data. We also illustrate the utility of surface maps that are localised at specific grey matter depths and suggest that such maps are a useful tool in studying or avoiding the disturbances that blood vessels can cause to fMRI analysis.

### Correcting inverted voxels can restore the lower boundary of hV4 in some hemispheres

Consistent with previous studies [19, 21, 23, 27, 28], we found that coverage in hV4 maps often does not extend over the entire hemifield. Specifically we find 7/20 lower quadrants in hV4 to cover < 20% of that region, with six of these being in the left hemisphere. Attempts to restore these maps by flipping the time courses of inverted voxels showed that it is possible to improve some of the incomplete maps, convincingly restoring a hemifield representation in the left hemisphere of Subject 10 and increasing the coverage above 20% in the left hemispheres of Subjects 3 and 5. Furthermore, hV4 maps in additional subjects were smoother post correction despite coverage in the lower quadrants remaining incomplete.

The case of Subject 10 serves as a clear example of a complete hemifield representation in hV4 that was obscured by an inadequacy of standard BOLD fMRI measurements. Our successful correction of this hV4 map highlights that some incomplete maps seem to be caused by inverted voxels being present along the lower boundary. These inverted voxels may be the result of venous artefact and can be accounted for using appropriate techniques. Nevertheless, we were unable to restore lower quadrant coverage in 4/7 incomplete hV4 maps.

### Correcting inverted voxels does not always improve hV4 maps

In contrast to the improvements seen in some hV4 maps, most changes post correction were inconsequential however, some deterioration was present in three (LH & RH; Subject 2; LH; Subject 9—these maps are presented in the Supplementary Materials (S1 File)). This is likely due to the correction of voxels with very weak, random negative correlations as evidenced by the results of thresholding the correction for the two subjects whom this affected. Here, we ran pRF models which only corrected inverted voxels with negative correlations stronger than -0.1; the resulting maps did not suffer from the same deterioration present in the maps generated by the non-thresholded correction (Fig 8).

It should be noted here that whilst for some subjects, weak negative correlations may be indicative of noisy voxels which cannot be corrected by inverting their time courses, the same may not be true of all such correlations. It is difficult therefore, to draw conclusions as to whether it is appropriate to threshold the correction, or to what extent a thresholding procedure could be equally applied across subjects.

### Venous eclipses do not always coincide with incomplete hV4 maps

We found little concordance between hV4 map coverage and the presence of venous eclipses in our depth-integrated mean intensity maps, with six incongruent pairings existing in our data. Furthermore, we find four cases of incomplete hV4 maps with no observable venous eclipse in the vicinity (LH; Subjects 10 & 11; RH; Subject 3)—close to half of the incomplete maps we identified.

A potential explanation for this unexpected discrepancy is that the intensity drop characteristic of venous artefact may be limited to voxels in the upper layers of grey matter, due to their closer proximity to surface veins. Averaging the responses of these superficial voxels with those from deeper layers of grey matter may obscure or weaken the amplitude drop of the venous eclipse to the extent it disappears or becomes difficult to identify. Essentially, not seeing venous artefact clearly in mean intensity maps may not signify its absence. Our depth-dependent surfaces support this idea, as they show the venous eclipse fade from being clearly recognisable in the 2.5mm surface to weakening in some parts of the 1mm surface before disappearing entirely in most sections of the grey/white surface.

### Clusters of inverted voxels are present in the region of the venous eclipse

We found inverted voxels to cluster in and around the venous eclipse in most hemispheres (see Fig 9 and the Supplementary Materials (S1 File) for examples), replicating the same finding by Puckett and colleagues [31]. We find similar percentages of inverted voxels in hV4 as Puckett and colleagues however, we note higher percentages in V1, V2 and V3 [31]. While it seems clear that inverted voxels are present at higher frequencies near venous artefact, it is difficult to ascertain whether voxels that appear nearby but outside the venous eclipse are actually in interstices created by the averaging of data from multiple grey layers. It is possible that these inverted voxels are in fact contaminated by venous artefact, which is unidentifiable in depth-integrated mean intensity maps. An alternative possibility is that the venous eclipse spuriously affects the time courses of voxels that lie lateral to its location, in addition to beneath it.

Our depth-dependent results suggest that clusters of inverted voxels are reasonably tightly restricted to the regions of grey matter which show venous artefact, especially in the 2.5mm surface. Responses directly neighbouring the outside of the venous eclipse in this layer show both high mean intensities and strong positive correlations, indicating that voxels which do not form part of the venous eclipse are not impacted by it. More depth-dependent analyses are required to confirm and better quantify this finding.

### Inverted voxels affect most visual areas equally

In contrast to Puckett and colleagues [31], we do not find percentages of inverted voxels to be higher in hV4 compared to V1, V2 and V3. This discrepancy with earlier work may be due to our larger pool of young adult subjects (N = 10, aged 21-26), compared to Puckett and colleagues, whose sample differed in size and age range (N = 4, aged 21-61). This is a potentially important difference, as neurovascular decoupling (where neural activity is not accompanied by compensatory changes in the dilation and constriction of blood vessels) has been reported to induce inverted BOLD responses in regions where blood vessels are impeded as a function of ageing and disease [52, 53]). The smaller number of subjects in Puckett and colleagues’ work may also explain this, as the TS has some natural variability in its proximity to hV4 [15]. The presence of a larger percentage of inverted voxels in one hV4 map due to the TS being more proximate to it may skew the overall findings more easily than in our pool of 10 subjects.

Additionally, there are differences in the extent of visual cortex stimulated in the present study and Puckett and colleagues (10° vs 20°), as well as with the stimuli and timing, which may account for the discrepancy in results. We do not find inverted voxel percentages to differ between the left and right hemisphere; this is consistent with an absence of differences in the number of venous eclipses between hemispheres.

### The pattern of inverted voxels changes with surface depth

In our depth-dependent data, the number of inverted voxels appears to decrease with depth, where within the venous eclipse many inverted voxels in the 2.5mm surface flip to positive in the 1mm and grey/white surfaces. Interestingly the reverse is true outside the venous eclipse where smaller, sporadic clusters of inverted voxels appear in deeper layers when the corresponding voxels in above surfaces show positive correlations.

We cannot say with certainty what may be causing these isolated inverted voxels however, they may simply be due to a lower SNR in deeper grey matter, increasing the frequency of random negative correlations. The sparse distribution and smaller clusters of these inverted voxels may also be the result of small veins from lower cortical layers, which dive through the grey matter from the cortical surface, bending at a right angle upon reaching the white matter before branching back up into lower grey layers (see Fig 14A) [50].

Regardless of the source of these inverted voxels, their impact can be seen clearly in the polar angle maps, where affected regions appear to map non-neighbouring areas of the visual field, disturbing the order of human visual cortex. The presence of these voxels stands in contrast to what is known about the retinotopic organisation of human visual cortex however is in agreement with findings of previous work [31].

### Hemispheric asymmetry in hV4 map coverage cannot be explained by a corresponding asymmetry in venous anatomy

**Fig 16:**
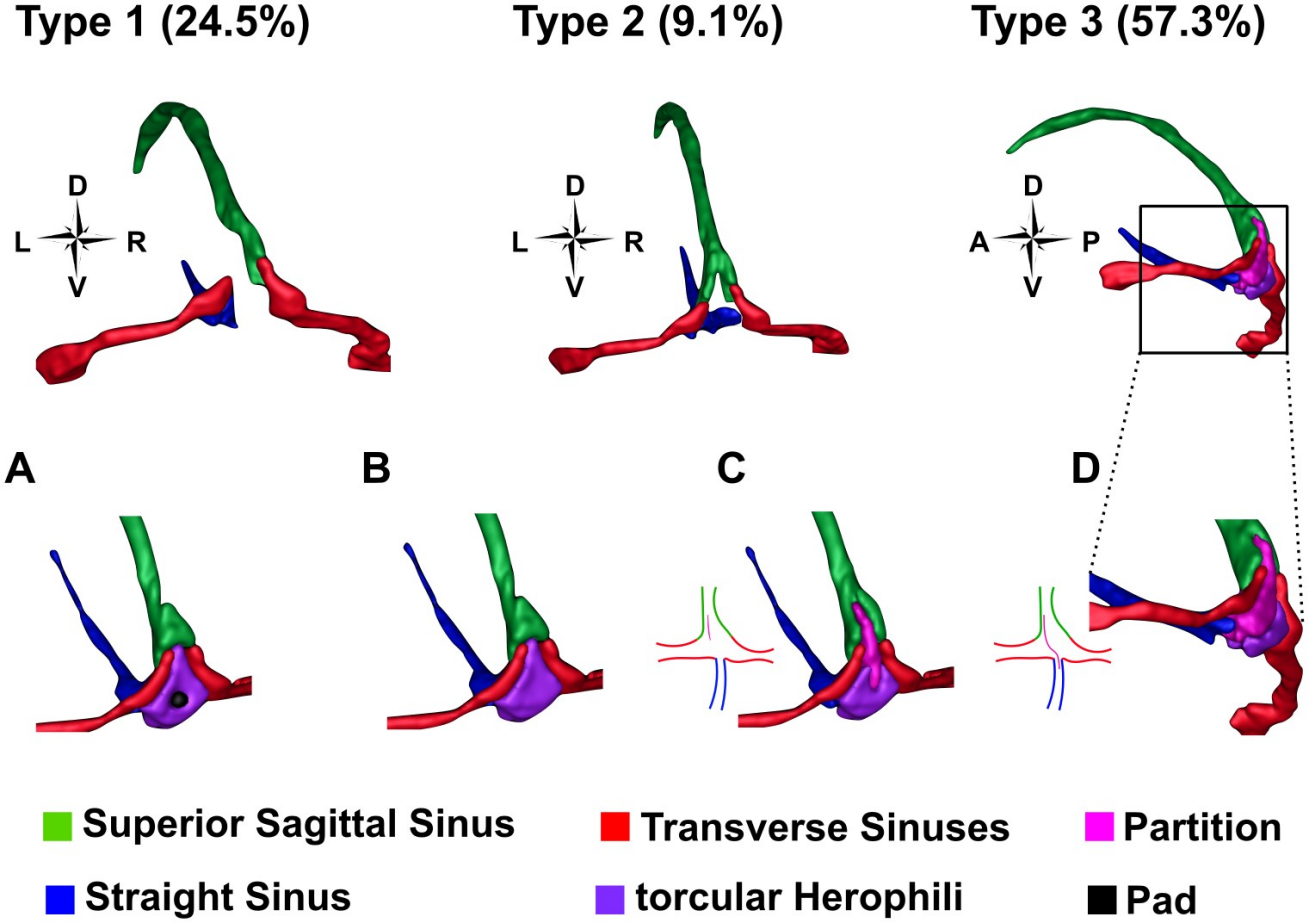
Three main variations of the venous anatomy of the dural sinuses. Percentages refer to the frequency with which each type is seen. In Type 1, the Superior Sagittal Sinus connects to one Transverse Sinus and the Straight Sinus to the other, with the two TSs completely separate from one another. In Type 2, the SSS and SS are forked, with the left forks connecting to the left TS, and the right forks to the right TS. In Type 3 there is some variation of a confluence of the sinuses. A) A ‘pad-like dural elevation’ in the tH [54]. B) Sinuses are connected by a confluence at the occipital pole. C) A partial partition extends from the SSS into the confluence of sinuses. This partition can be closer to the left or the right wall of the SSS. D) A full partition extends from the SSS diagonally through the tH to the SS. The full partition can extend diagonally across from closer to the left or the right wall of the SSS. This figure was created by segmenting the venous anatomy from the venogram of Subject 4, who had Type 3A. Other variations were approximated based on the descriptions in [54].

Having been noted in previous work and corroborated here, the bias towards left hemisphere hV4 maps being incomplete more frequently than right hemisphere maps is a robust finding that warrants explanation. Given that the TS and large surface veins in general give rise to the venous eclipse, consideration is due to the physical anatomy of veins in the occipital cortex. Individual variations exist at the torcular Herophili (tH; the confluence of sinuses at the occipital pole) [54, 55], where variations are separated into three broad categories, depicted in Fig 16. Of particular note is Type 1, where there is an *absence* of a confluence and the right and left transverse sinuses connect to only one of the Superior Sagittal Sinus (SSS) and Straight Sinus (SS) [54, 55] (see ‘Type 1’ in Fig 16).

Differences at the tH are important to note, as research has shown the signal recorded in areas of large draining veins may be reflective of changes in distal regions of cortex [14]. As such, in subjects where the SSS and SS drain into separate TSs, the responses being measured in the region of the TS come from non-homologous regions of each cerebral hemisphere. Specifically, the SSS would be fed by lateral regions of the anterior cerebral hemisphere and the SS by the cerebellum and centre of the head [26]. For hV4 maps belonging to subjects with this venous anatomy, signal recorded in the region of hV4 in the left and right hemispheres may be asymmetric, as the blood in the TSs would be originating from two distinct brain regions. However it is important to note that Type 1 represents only 24.5% of cases and the considerable variability of TS anatomy is difficult to reconcile with the relatively consistent finding of more incomplete left hemisphere hV4 maps.

Alternatively, a bias in TS size, whereby the left TS tends to be larger could serve as a potential explanation, as a higher concentration of deoxyhaemoglobin near left hemisphere hV4 maps should increase the chances of finding incomplete hemifields. However an investigation of 110 adult cadavers found the opposite—a (small) bias towards the right TS being larger—a finding that has been replicated recently [54, 55]. Together this suggests that a strong association between hV4 map coverage and venous anatomy is yet to be consolidated and further work in this area is required.

### Susceptibility artefacts may account for hemispheric asymmetry

As there is a low likelihood that hV4 maps vary so considerably in their representation of the visual field between the left and right hemispheres as found here and elsewhere [19, 21, 23, 27, 28, 56], venous asymmetry is but one of multiple potential causes. Another possible explanation of the hemispheric asymmetry in our hV4 maps is the fact that the phase encoding direction used here (also known as the ‘blip’ or ‘fat-shift’ direction) was left to right.

This has the effect of distorting the EPIs in the same direction, as a result of susceptibility artefacts induced by various types of tissue [57, 58]. However, though sufficient as a hypothesis for our data, the same leftwards asymmetry reported by Winawer and colleagues cannot be attributed to a left to right phase encoding direction, as this data was acquired using a spiral sequence [15]. Nevertheless, the direction of phase encoding is worth taking into consideration when interpreting findings of hemispheric asymmetry in fMRI data, particularly when no known explanation for the asymmetry exists.

### Negative BOLD responses versus inverted voxels

**Fig 17:**
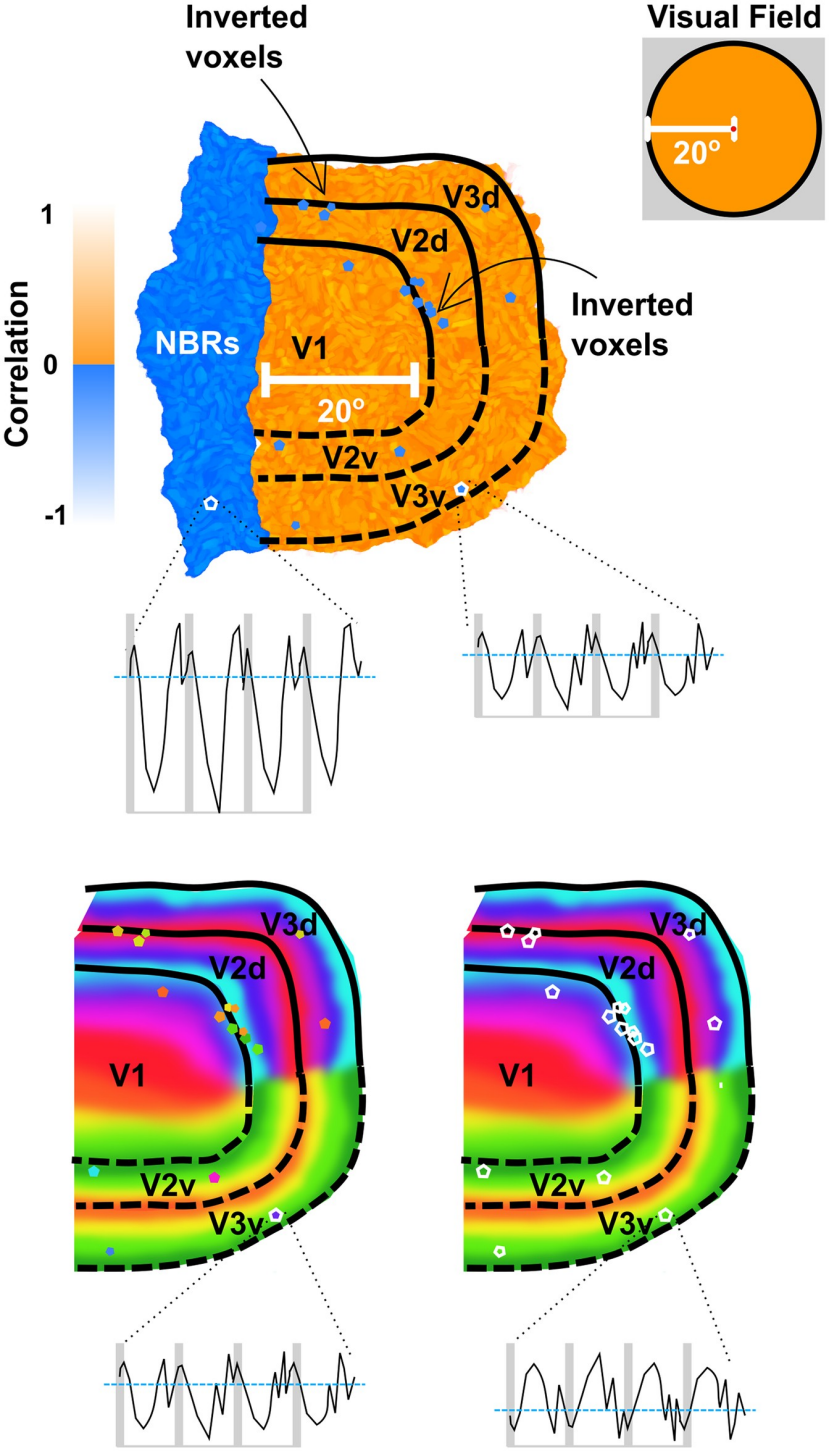
Negative BOLD responses vs. inverted voxels in visual cortex. NBRs form a large cluster immediately outside the eccentricity range of the stimulated region of the visual field. Inverted voxels are located within this range and are more isolated or form smaller clusters, often associated with venous artefact. NBRs have stronger and less noisy time courses. The correction of inverted voxels as shown in the bottom panel produces more coherent retinotopic maps, whereby the phase of corrected inverted voxels match those of their neighbours.

Thus far, we have been considering the impact of inverted voxels on retinotopic maps; it is important here to distinguish these inverted voxels from Negative BOLD Responses (NBRs). We found that voxels showing NBRs were arranged in large clusters located adjacent to but mostly outside the region of cortex that fell within the eccentricity of our stimuli. While many details of the specific drivers of NBRs are still a matter of debate, it is likely that they are due to neural mechanisms and a reduction of neuronal activity [31, 44, 45, 59], as opposed to the haemodynamic mechanism proposed to cause inverted voxels [31]. The dense clustering of NBRs in the region immediately bordering the outside edge of the stimulus where lateral inhibitory effects would be expected clearly demarcates these responses from inverted voxels that lie within the stimulated region of cortex, where such effects would be unexpected. Although we are unable to guarantee that ‘inverted’ voxels are not being selectively inhibited despite encoding a stimulated region of the visual field, this would stand in disagreement with what is known about physiology and retinotopic mapping and hence it is likely that ‘inverted’ voxels and NBRs have different causal mechanisms [5, 29, 31, 60–62]. Differences between NBRs and inverted voxels are summarised in Fig 17.

Beside the differences in their location, we note a number of additional differences between ‘inverted’ voxels and NBRs. NBRs have stronger correlations and higher mean intensities than inverted voxels, often on par with positive adjacent voxels that encode positions within the stimulated region of the visual field (see Fig 11). Inverted voxels also appear more sporadically and in smaller clusters than NBRs (unless they clearly lie within regions likely to be affected by venous artefact from overlying sinuses (see Fig 14)). Time courses of inverted voxels additionally tend to be noisier and there are fewer of them than NBRs (see Fig 12).

The strong and reliable signal recorded from voxels exhibiting NBRs enabled the convincing extension of retinotopic maps of early visual areas after flipping negatively correlated time courses. This allowed for the delineation of the visual field map of V1 beyond the range of our visual display system. As we did not directly stimulate this region of the visual field, these extensions should not be thought of as analogous to directly measured retinotopic representations until a formal relationship between these has been established. Nevertheless, we are confident that the region shown in the ‘extended’ V1 map in Fig 13 is in retinotopic cortex, as we only stimulated 10°of visual field. Our results support previous suggestions that NBRs are the result of suppression, most likely caused by lateral inhibition [45, 63].

### Limitations/Considerations from the depth-dependent data

Examination of the depth-dependent data shows that the venous eclipse is most prominent on superficial surfaces, which is to be expected as these are in close proximity to surface veins. Based on this depth-dependent analysis, it appears that the grey/white boundary may be impacted very little by venous artefact, at least in terms of the mean intensity of voxels in cortex underlying surface veins. The polar angle maps depicted in Fig 14 clearly show an incomplete map of hV4 at the superficial surface, despite the fact there is no venous artefact visible along the lower boundary and where mean intensity and correlation responses are comparable to surrounding regions that are unaffected by venous artefact. At the 1mm and grey/white depths, the map of hV4 still appears to be incomplete.

Careful examination of the region surrounding the lower hV4 boundary in Fig 14 shows some voxels which appear to respond to the lower vertical meridian however, as the correlation maps show that these voxels also exhibit weak or inverted responses, it is difficult to trust the retinotopic mapping in this region. It must be noted that the conservative criteria we used in classifying whether hV4 maps were likely to be impacted by venous artefact resulted in the inclusion of this data as a congruent *Incomplete hV4/Venous eclipse present* pairing in the depth-integrated map. Examination of this data across different grey matter depths indicates that the venous eclipse could not reasonably be expected to impact responses along the lower hV4 boundary and hence this hemisphere might be more accurately classified as an incongruent *Incomplete hV4/Venous eclipse absent* pairing.

Although the depth-dependent analysis formed only a peripheral part of this work, we believe that our findings here are important for several reasons. Firstly, we show that depth-dependent analyses can be performed on medium resolution fMRI data (1.5mm) and that doing so yields additional, meaningful information about the responses of voxels at different cortical depths. Most importantly we demonstrate that it is possible to locate regions that are impacted by what appears to be a venous eclipse with a high level of spatial specificity. In future work, this will enable more accurate predictions of where venous artefact can be expected to impact retinotopic maps in other fMRI analyses, including beyond the visual cortex. Finally, our results suggest that regions outside the venous eclipse do not appear to be impacted by it and deeper cortical layers show only minor impacts of surface veins on voxel responses.

While these findings are useful in helping inform interpretations of our main dataset, it is not known how replicable these findings will be across subjects. A further limitation of these results is that our independent depths are based on the grey/white surface alone, rather than additionally including the pial surface as an upper boundary. Although grey matter thickness in visual cortex is relatively consistent [47], including the pial surface as an upper limit would increase the reliability of findings as we would not be assuming a constant grey matter thickness.

### Implications and future work

In this study we replicate and extend findings from Puckett and colleagues suggesting that inverted voxels can be corrected to produce smoother retinotopic maps in early visual cortex. While this procedure did not resolve every incomplete map of hV4, it may be a useful method to apply in cases where inverted voxels cause disturbances in the order of retinotopic maps.

Debate surrounding the extent of retinotopic coverage in hV4 has largely focused on polar angle representations, due to the ability to demarcate visual area boundaries based on this information. Fewer studies have considered the eccentricity representation present in hV4 [19, 28] and the location of the venous eclipse in relation to eccentricity maps has not yet been examined. Given that the size of pRFs plays a role in visual field coverage and changes in pRF size occur with both visual area and eccentricity [15, 34], closely examining eccentricity maps would be useful in creating a deeper understanding of the impacts of venous artefact in vulnerable regions of visual cortex.

### Conclusion

We demonstrate a hemispheric asymmetry that is biased towards incomplete maps of hV4 appearing predominantly in the left hemisphere, a finding that has never been thoroughly discussed despite being corroborated in multiple earlier studies and for which an explanation is wanting [19, 21, 23, 27, 28]. The leftwards bias found for incomplete hV4 maps cannot be explained by a corresponding anatomical bias of the TS [54], suggesting that venous eclipses can explain some, not all, incomplete hV4 maps. It appears to be the case that an additional, more consistent source of the hemispheric asymmetry must exist to explain these incomplete maps and we suggest this cause should be more consistently biased towards the left.

Venous eclipses and inverted voxels have both been proposed as explanations for incomplete maps of hV4 [15, 31]. We confirm and support previous findings identifying cases where inverted voxels cluster in regions of the venous eclipse and we show that correcting time courses of inverted voxels convincingly restored a complete hemifield map of hV4 on the ventral surface in the left hemisphere of Subject 10 as well as improving the disrupted retinotopic maps in several others. This strongly supports the notion that hV4 maps are complete but may appear incomplete due to inadequacies in measurement.

## Supporting information

S1 File Supplementary materials. These supplementary materials contain three sections. Section 1 (p. 1-20): Figures of the left and right hemisphere hV4 maps for all 10 subjects, showing the mean intensity, correlation, polar angle pRF and corrected pRF maps, as well as visual field coverage plots and smoothness plots of hV4 from the original and corrected analyses. Section 2 (p.21): A 2D histogram plotting voxel density as a function of mean intensity and correlation for all 10 subjects. Section 3 (p.22-26): Raw percentages tables of hemifield quadrant coverage in V1-V4 for all 10 subjects and V4 percent coverage post correction.
(PDF)
