# Supplementary material for: Vascular effects on the BOLD response and the retinotopic mapping of hV4
Source: PLoS One. 2019 Jun 13;14(6):e0204388. doi: 10.1371/journal.pone.0204388 (PMC6563965; doi:10.1371/journal.pone.0204388)
Supplement: S1 File — These supplementary materials contain three sections. Section 1 (p. 1-20): Figures of the left and right hemisphere hV4 maps for all 10 subjects, showing the mean intensity, correlation, polar angle pRF and corrected pRF maps, as well as visual field coverage plots and smoothness plots of hV4 from the original and corrected analyses. Section 2 (p.21): A 2D histogram plotting voxel density as a function of mean intensity and correlation for all 10 subjects. Section 3 (p.22-26): Raw percentages tables of hemifield quadrant coverage in V1-V4 for all 10 subjects and V4 percent coverage post correction. (PDF) [file pone.0204388.s001.pdf]

Vascular effects on the BOLD response and the retinotopic mapping of hV4 – Supplementary Materials

These supplementary materials contain four maps of hV4 for each hemisphere of our 10 subjects – mean intensity, correlation, polar angle pRF and corrected polar angle pRF maps, visual field coverage plots and smoothness plots of hV4 from the original and corrected analyses and tables of the raw percentages of hemifield quadrant coverage in V1-V4 for all subjects, as well as V4 post correction percent coverage.

Sub-01 – Left Hemisphere

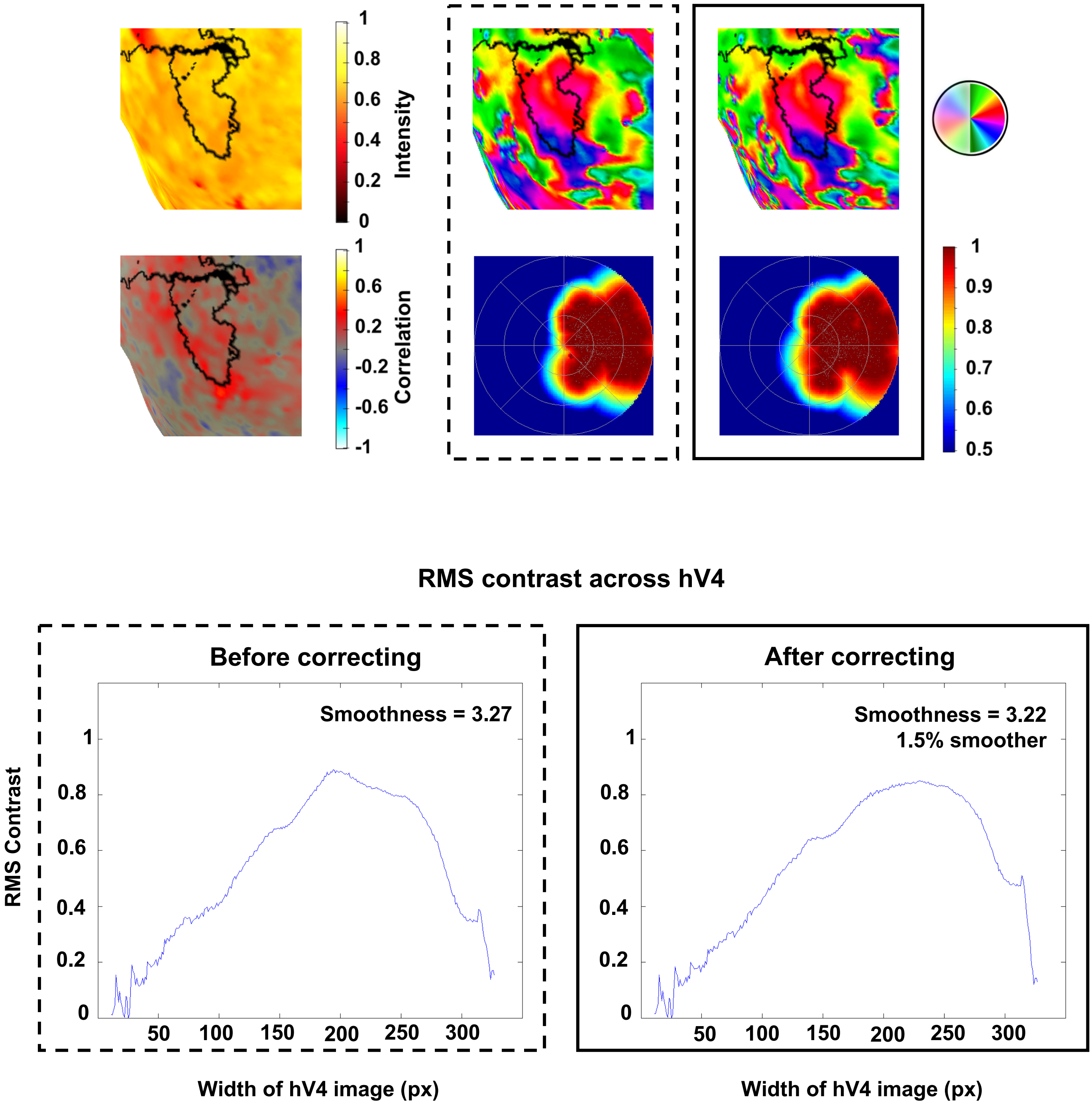

Subject 1 LH. Minimal changes are seen in hV4 after flipping inverted voxel time courses.

Sub-01 – Right Hemisphere

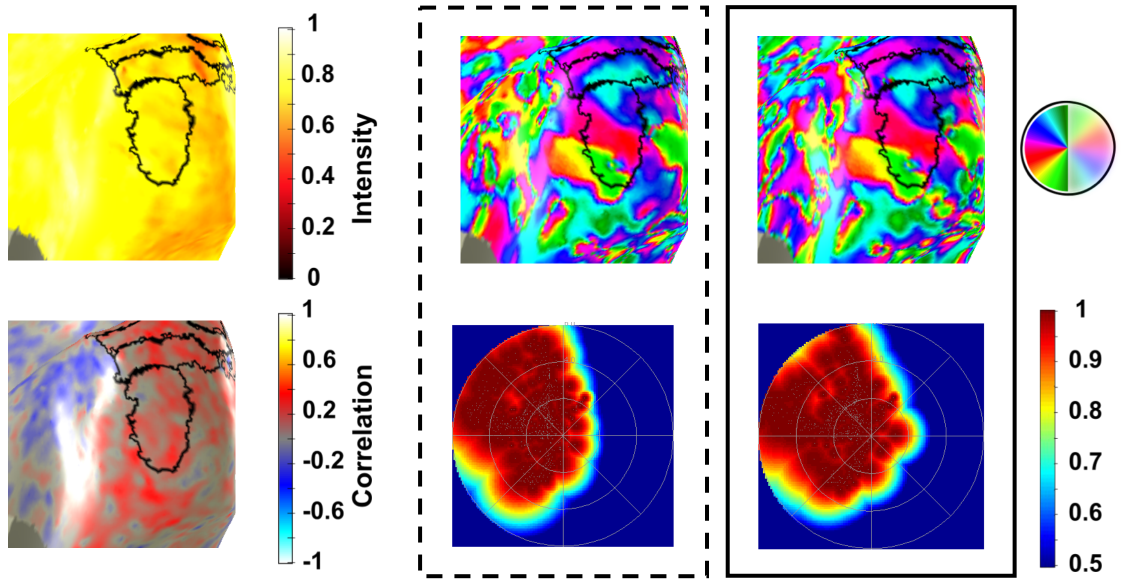

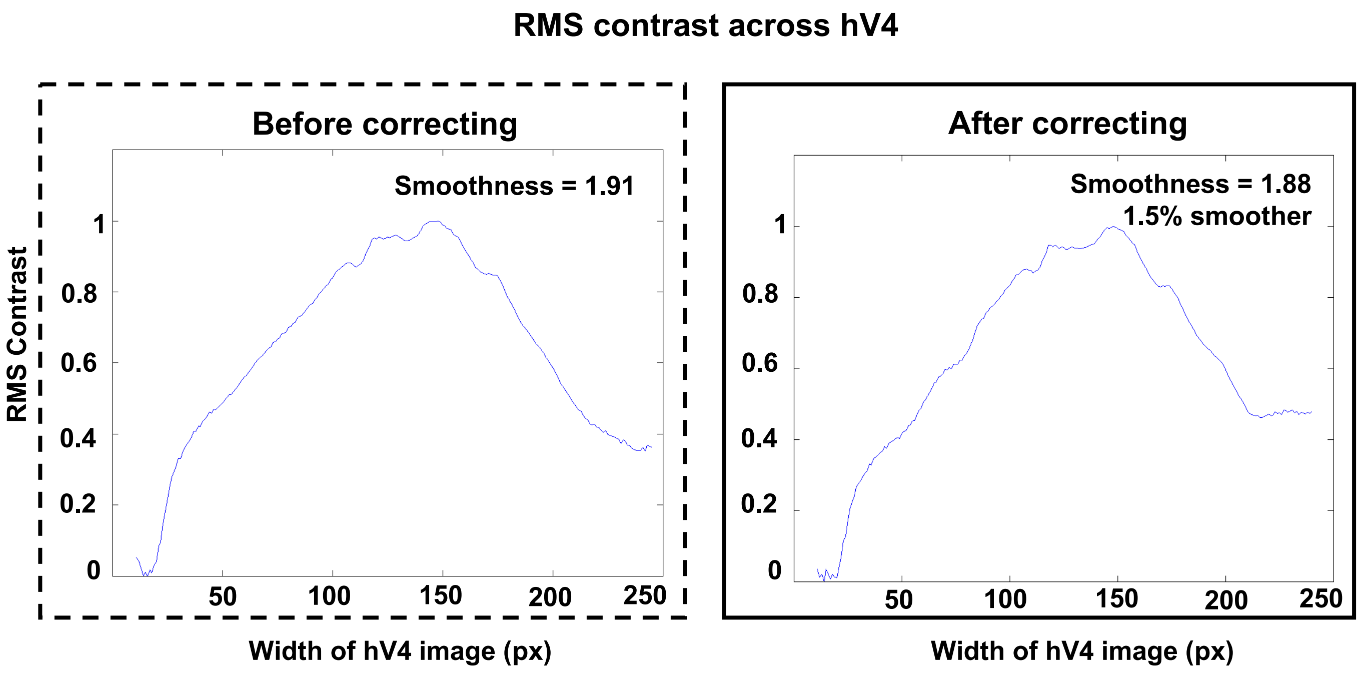

Subject 1 RH. Minimal changes are seen in hV4 after flipping inverted voxel time courses.

Sub-02 – Left Hemisphere

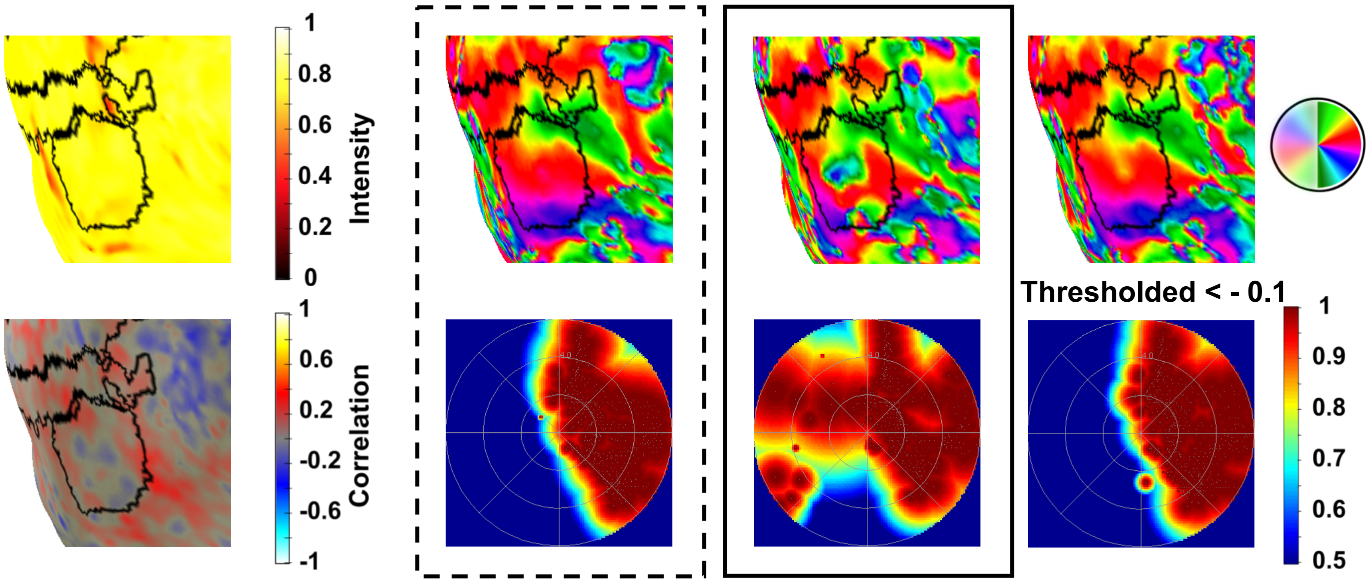

RMS contrast across hV4

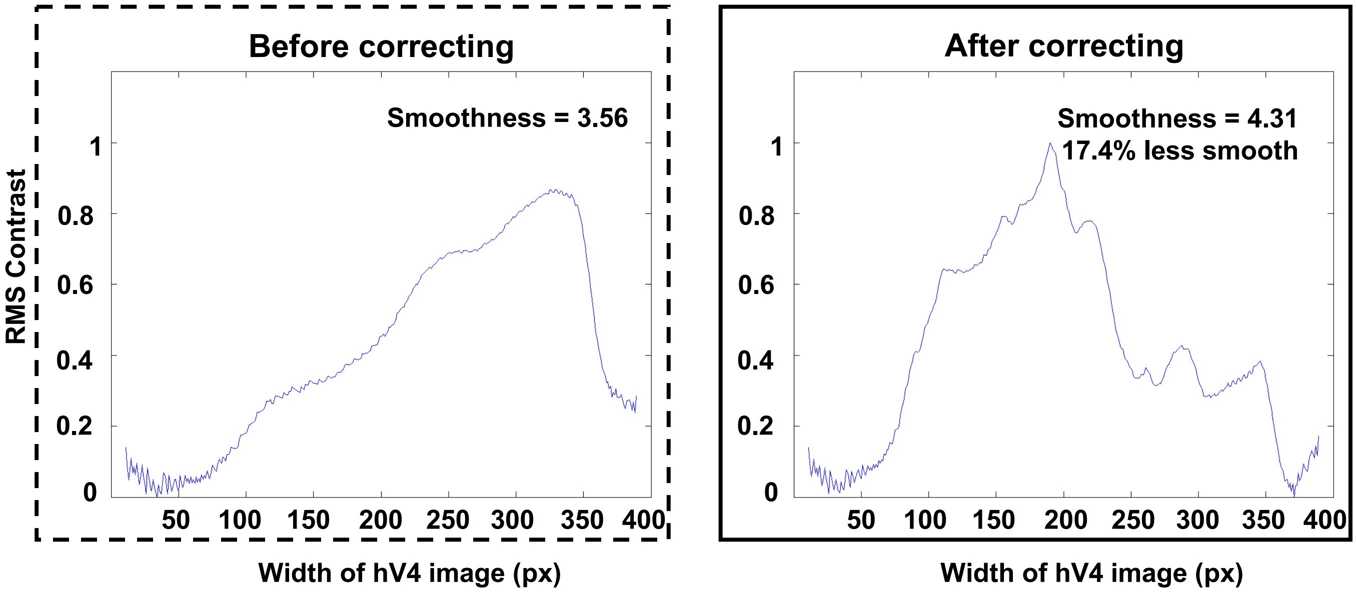

Subject 2 LH. The correlation map shows a large number of inverted voxels in hV4 however this is not reflected in the form of disturbed polar angle map prior to flipping inverted voxels. Venous artefact can be seen surrounding the hV4 map in the left hemisphere. hV4 mapping and smoothness are worse after correcting inverted voxels. Thresholding out weak correlations improves this

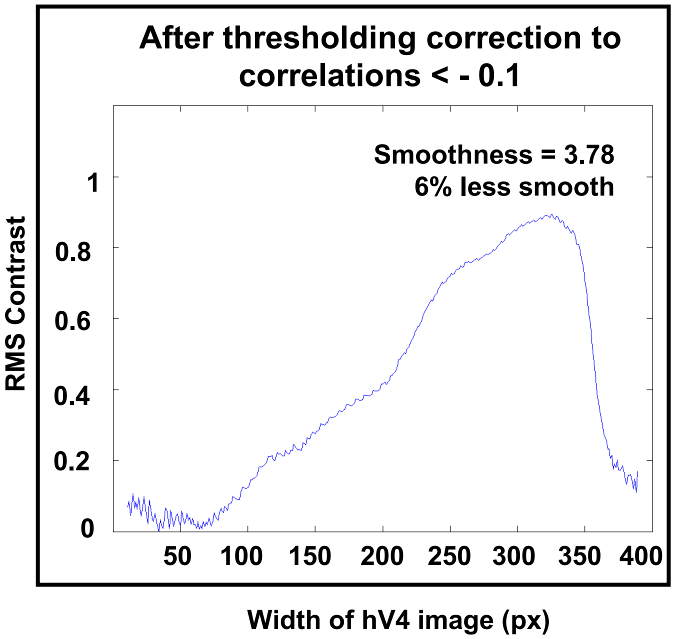

Sub-02 – Right Hemisphere

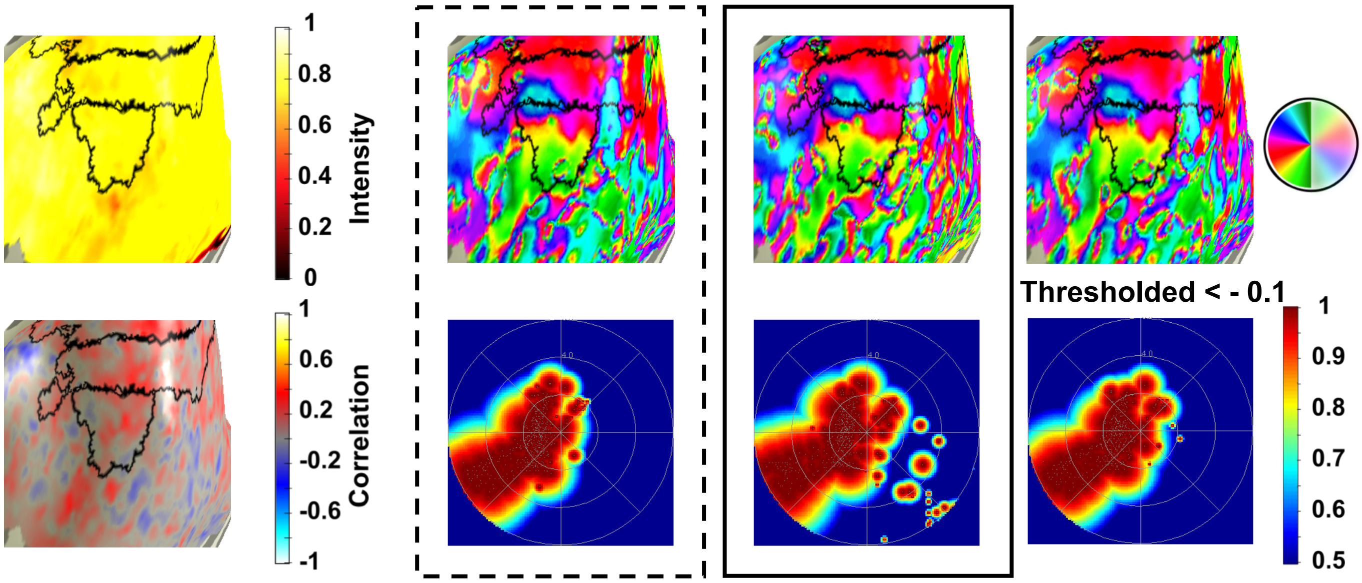

RMS contrast across hV4

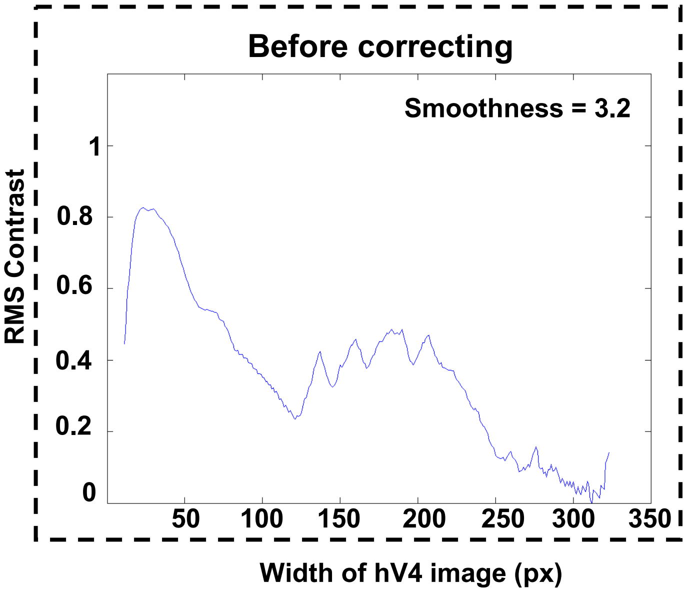

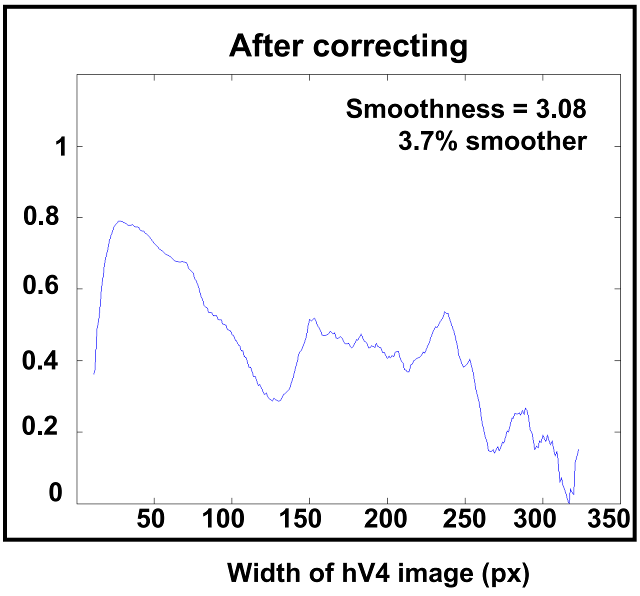

Subject 2 RH. Minimal changes are present in the hV4 map after flipping inverted voxels; there is slightly more ipsilateratal coverage present in the correcting map, which improves after thresholding weak negative correlations (<-0.1).

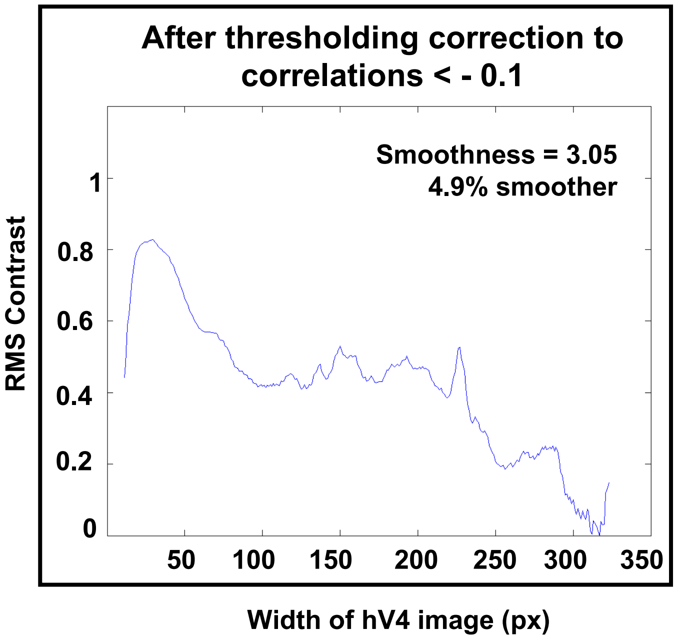

Sub-03 – Left Hemisphere

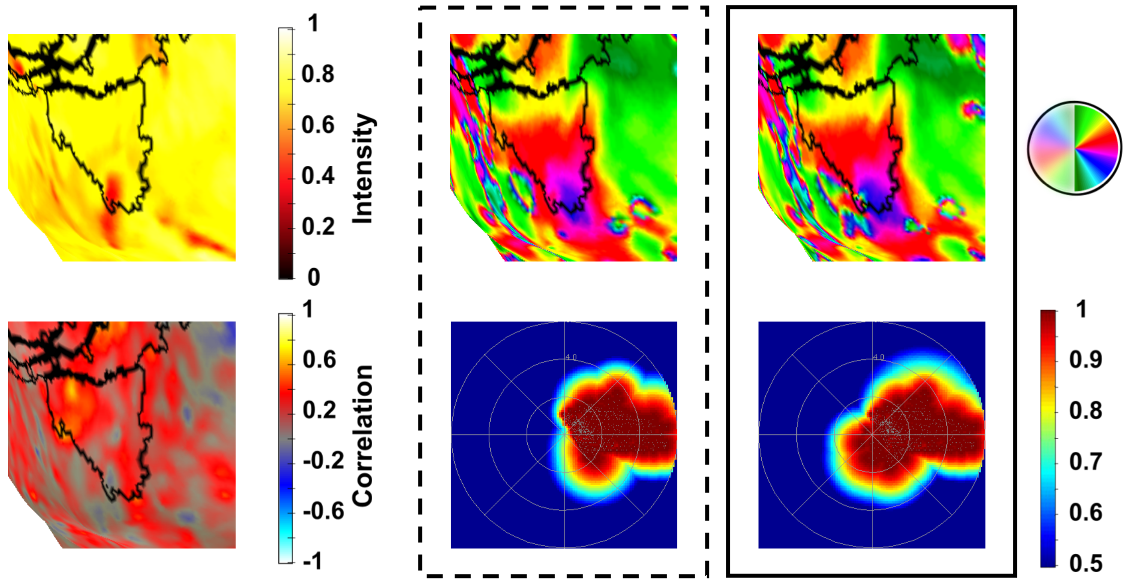

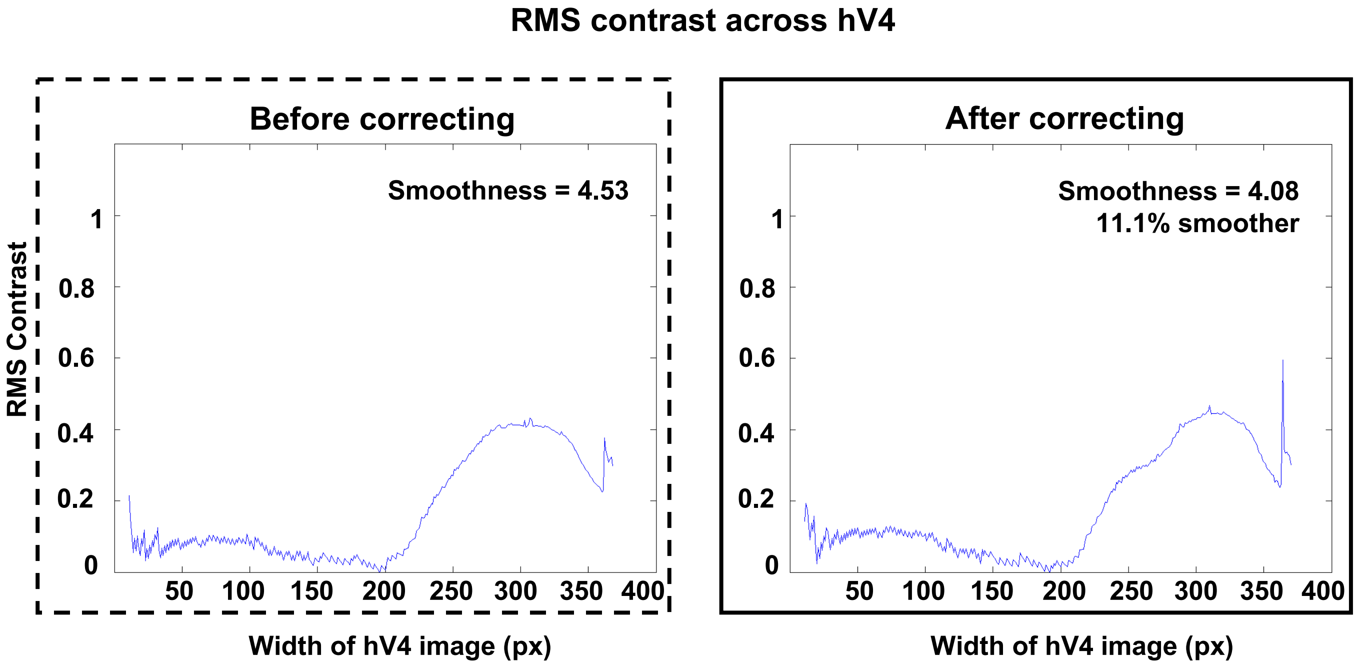

Subject 3 LH. Minimal changes are seen in hV4 after flipping inverted voxel time courses.

Sub-03 – Right Hemisphere

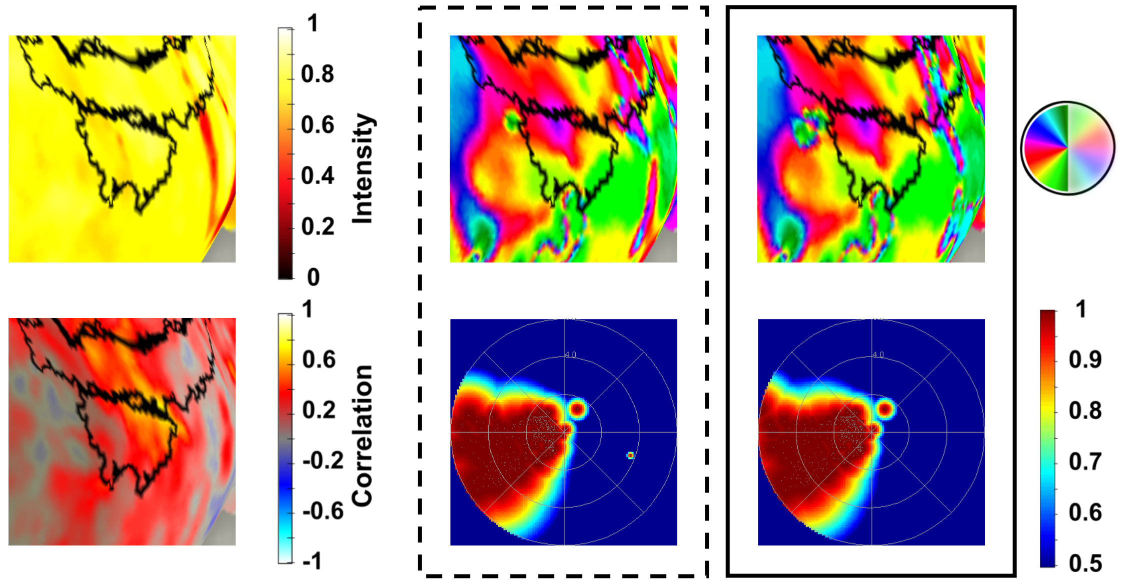

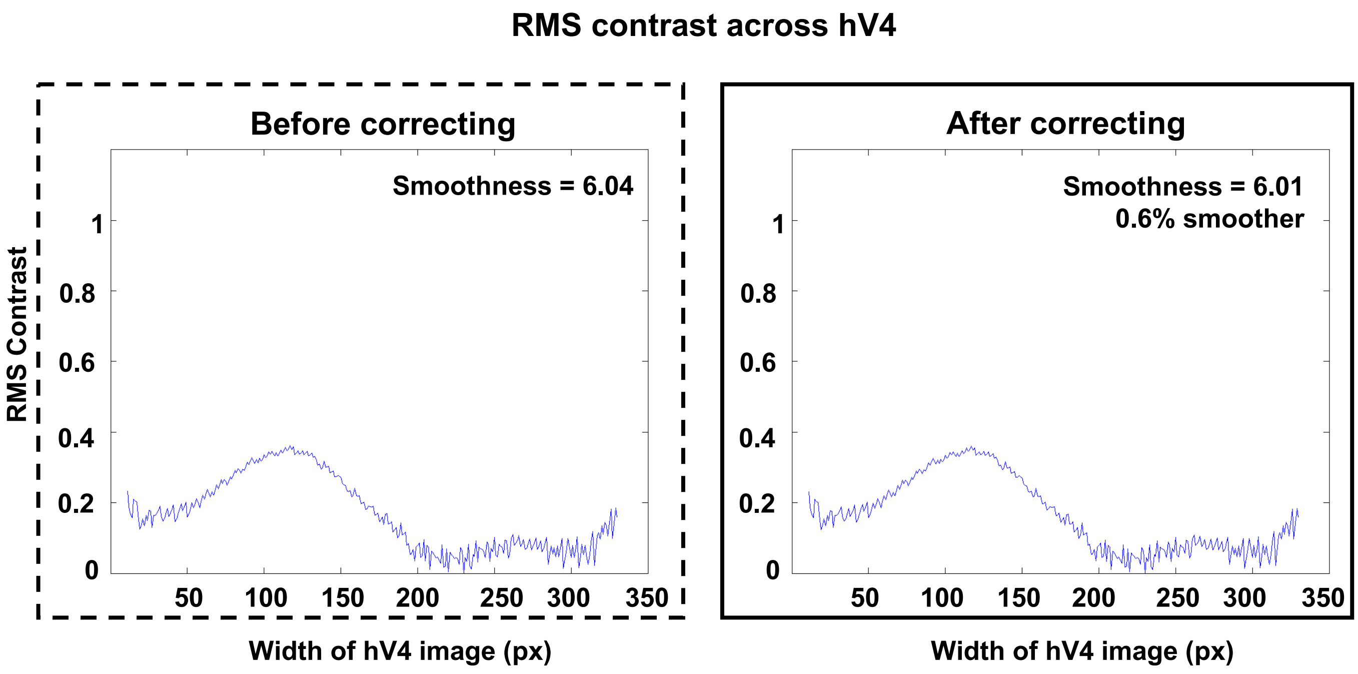

Subject 3 RH. Minimal changes are seen in hV4 after flipping inverted voxel time courses.

Sub-04 – Left Hemisphere

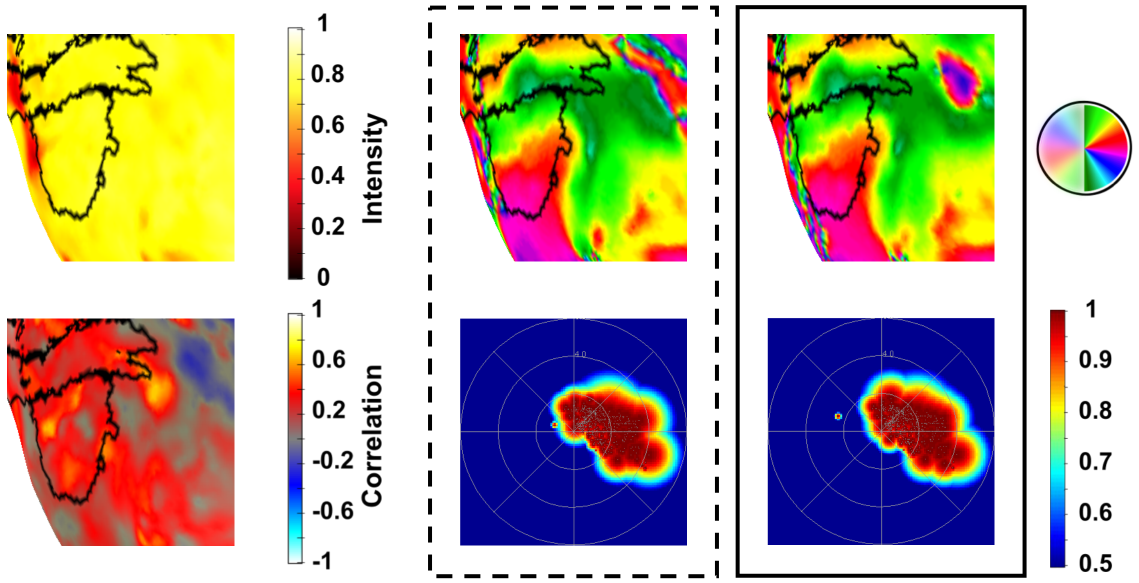

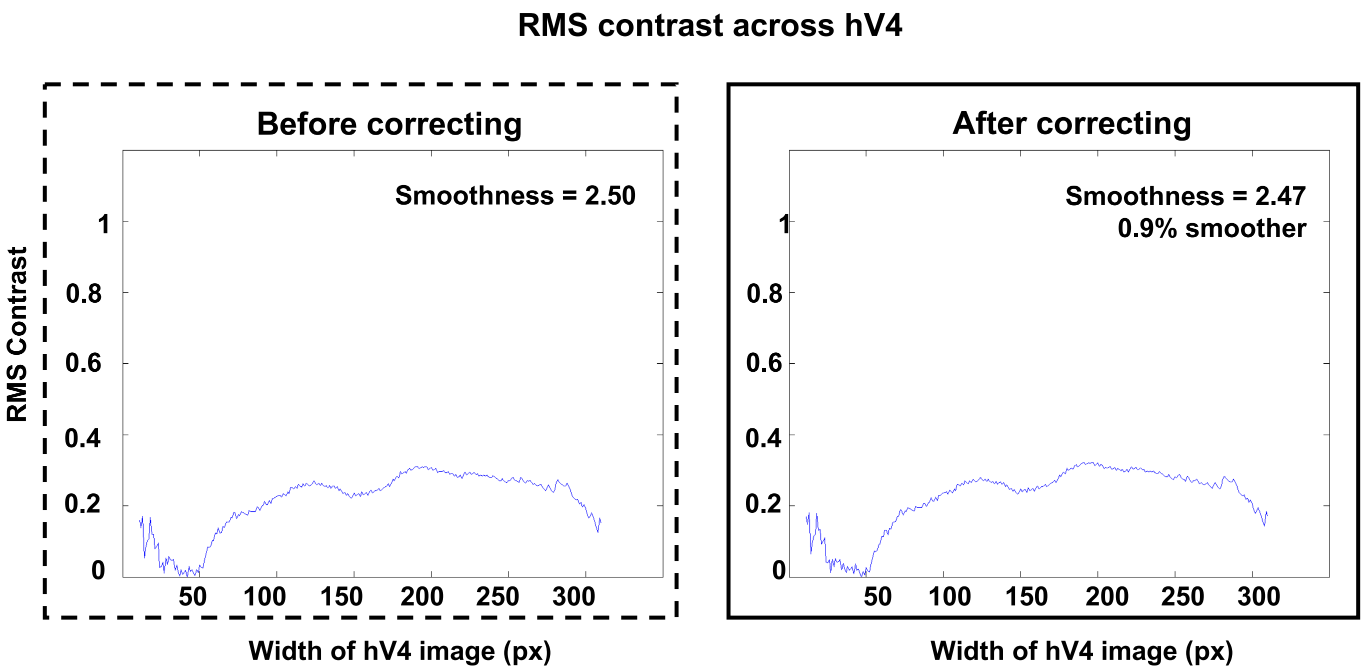

Subject 4 LH. Minimal changes are seen in hV4 after flipping inverted voxel time courses.

Sub-04 – Right Hemisphere

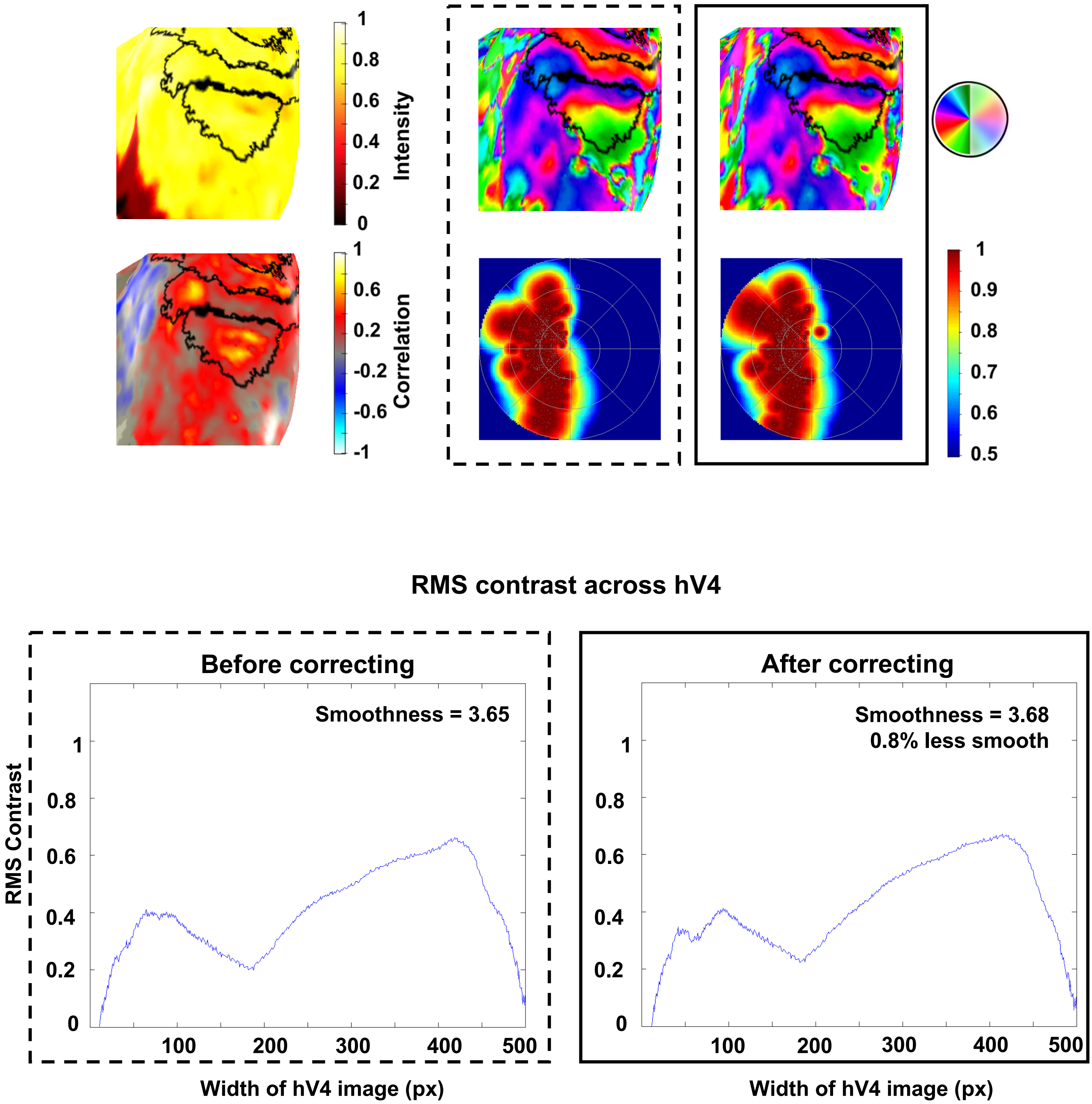

Subject 4 RH. Minimal changes are seen in hV4 after flipping inverted voxel time courses.

Sub-05 – Left Hemisphere

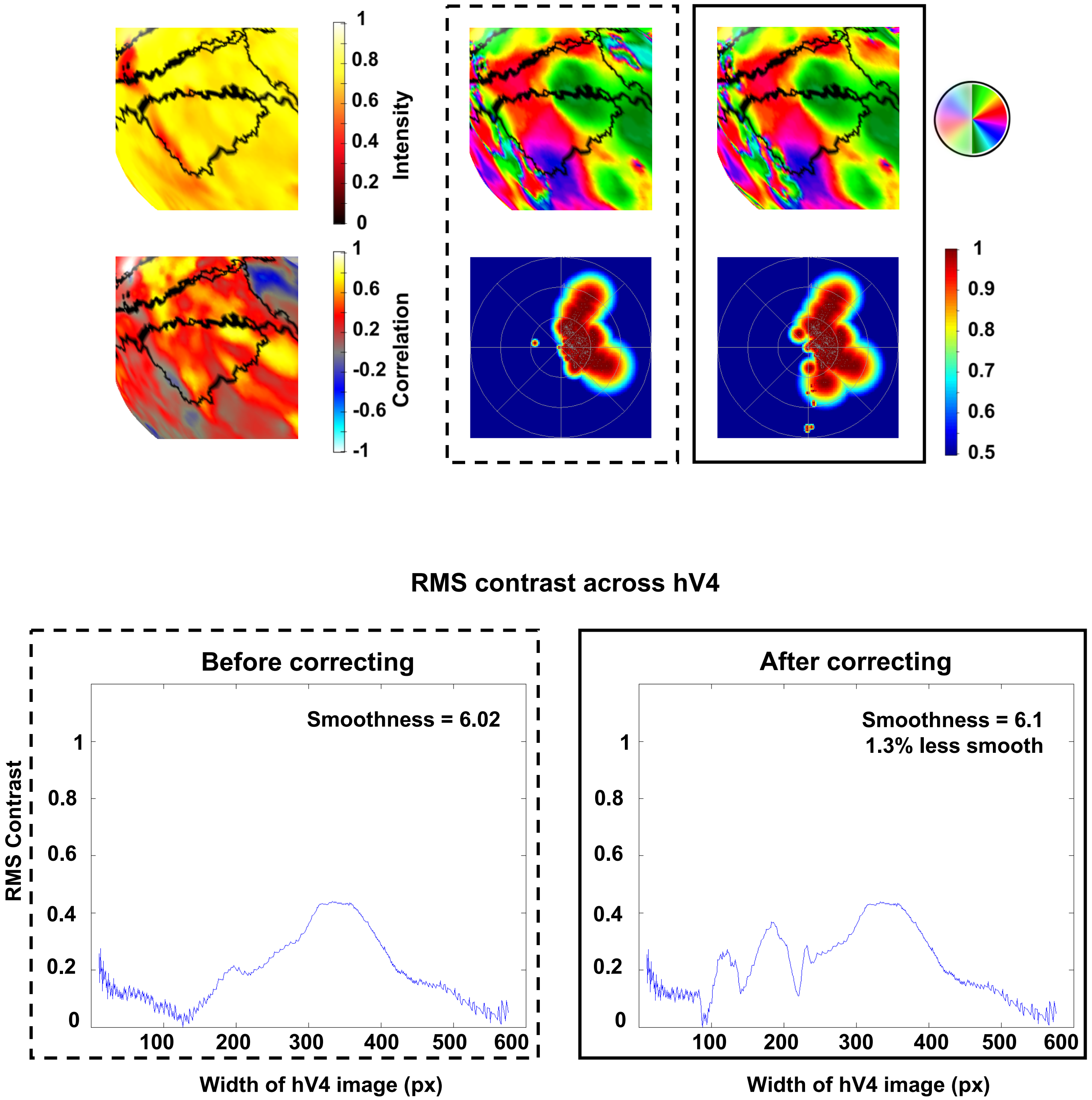

Subject 5 LH. Minimal changes are seen in hV4 after flipping inverted voxel time courses.

Sub-05 – Right Hemisphere

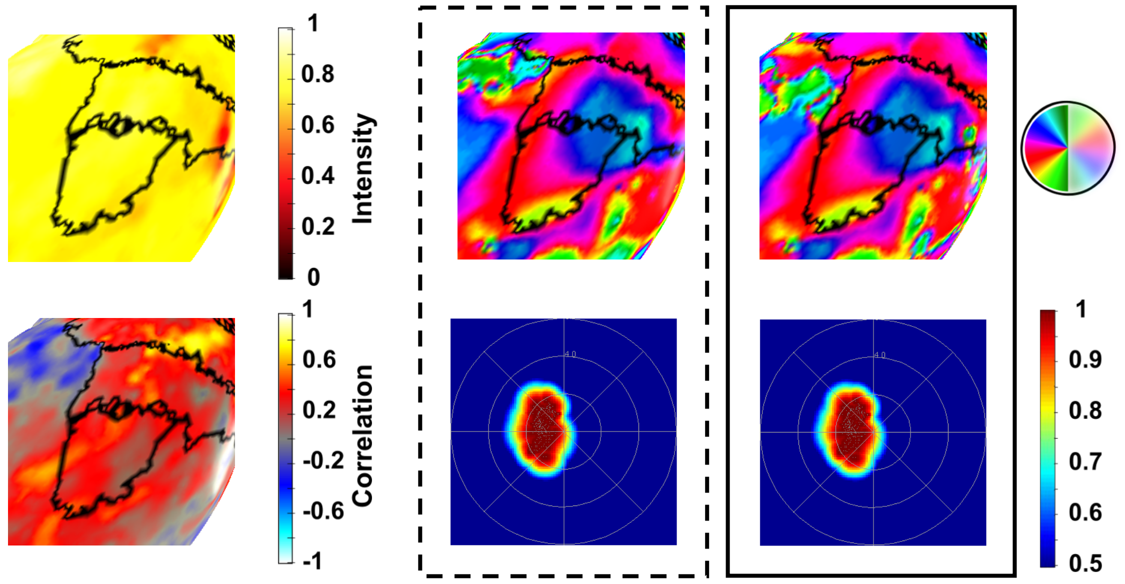

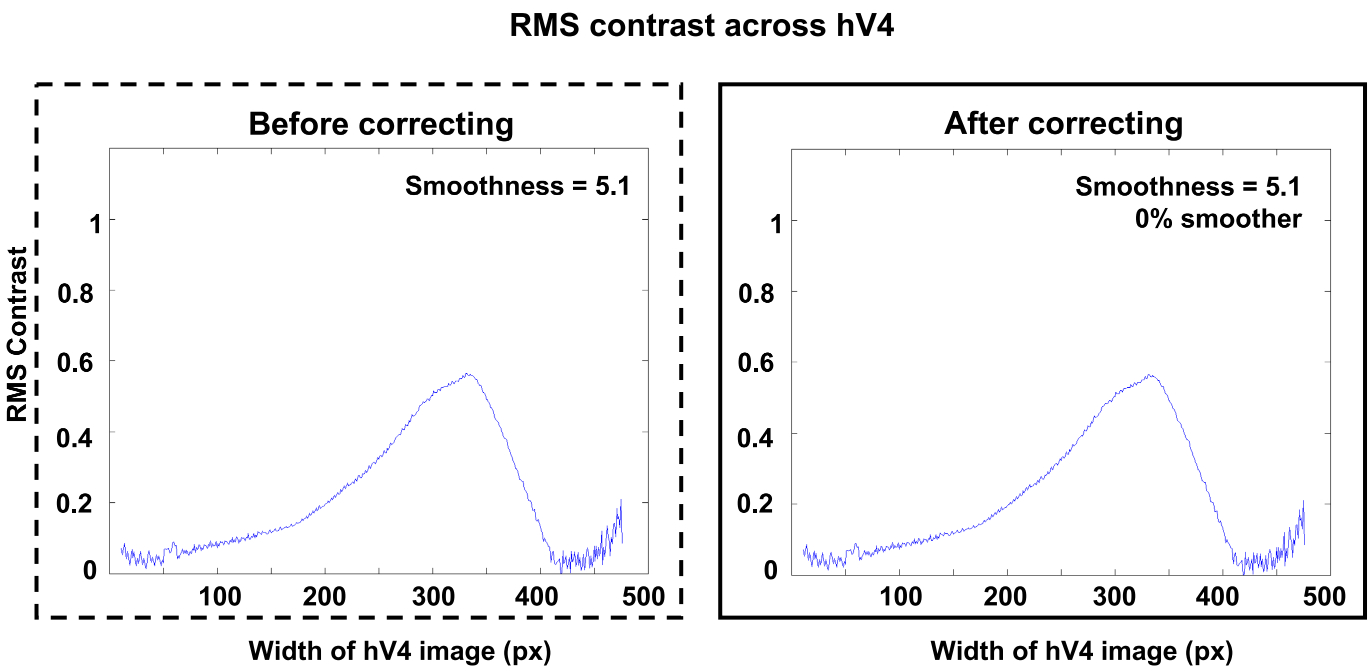

Subject 5 RH. Minimal changes are seen in hV4 after flipping inverted voxel time courses.

Sub-06 – Left Hemisphere

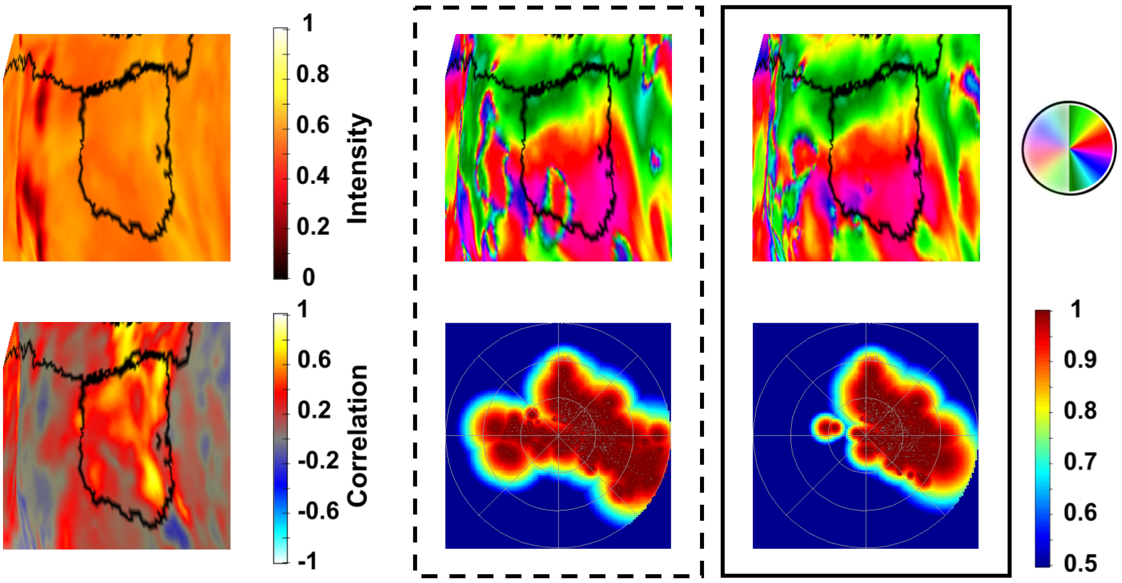

RMS contrast across hV4

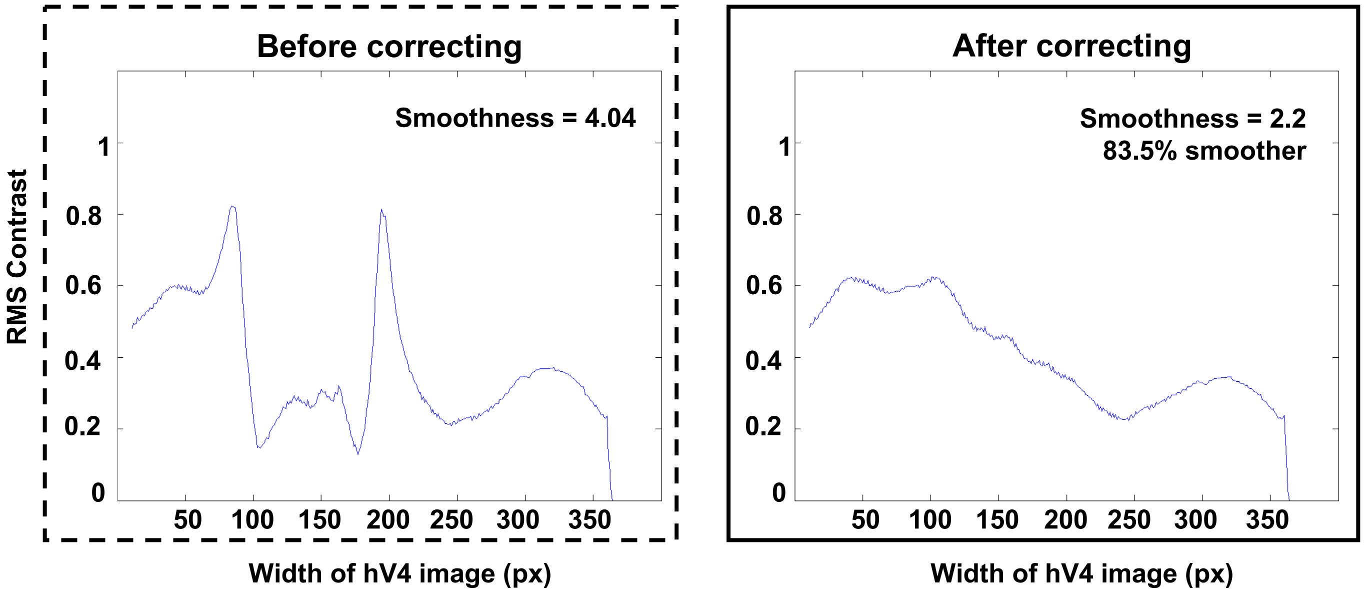

Subject 6 LH: A disturbed polar angle map is recorded along the lower boundary prior to flipping inverted voxels, which is corrected after flipping inverted voxels. This is reflected in fewer voxels appearing to respond to the ipsilateral hemifield in the visual field coverage plot and a much smoother map post-correction.

Sub-06 – Right Hemisphere

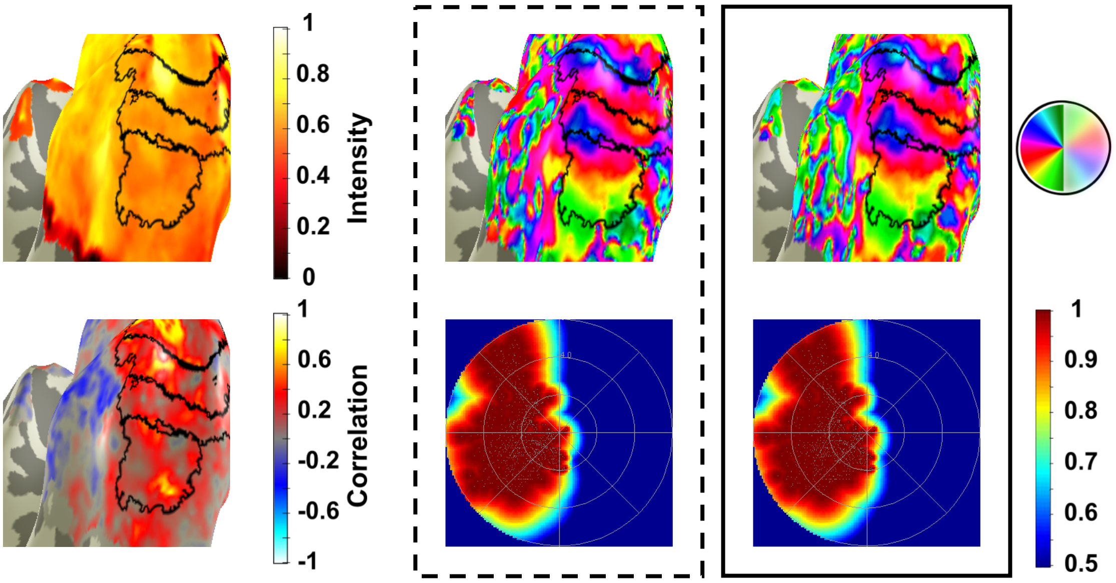

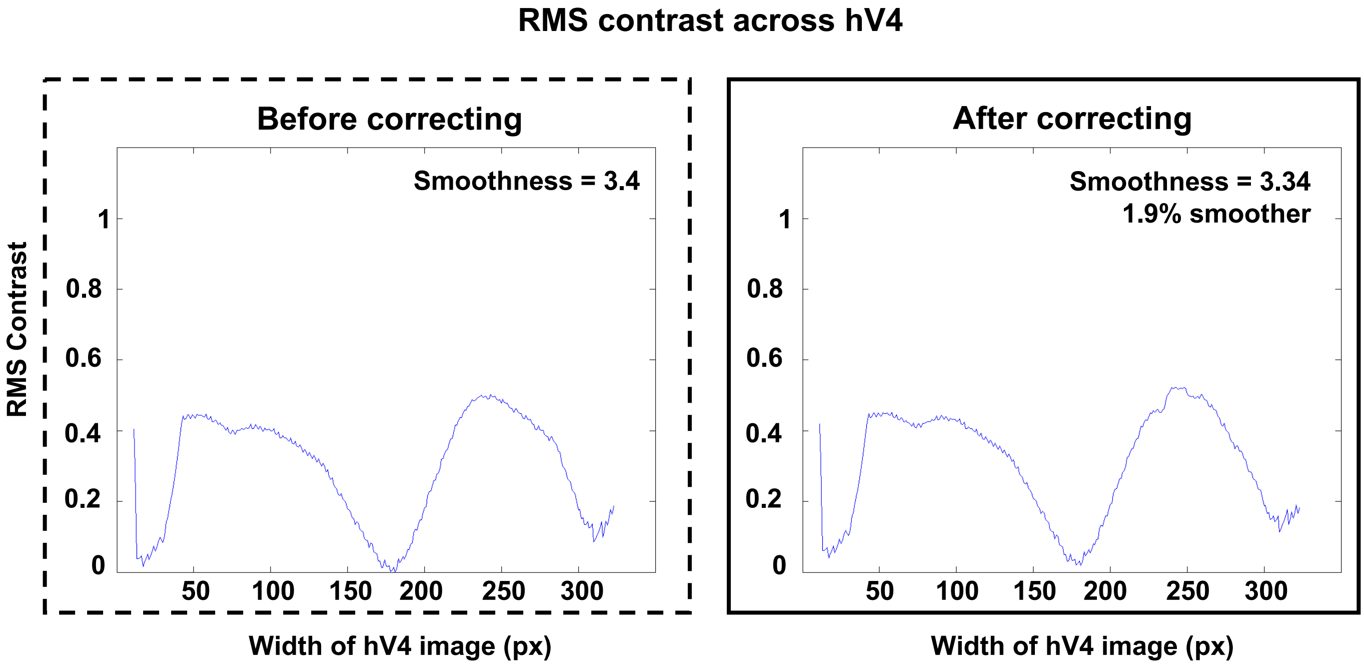

Subject 6 RH. Minimal changes are seen in hV4 after flipping inverted voxel time courses.

Sub-08 – Left Hemisphere

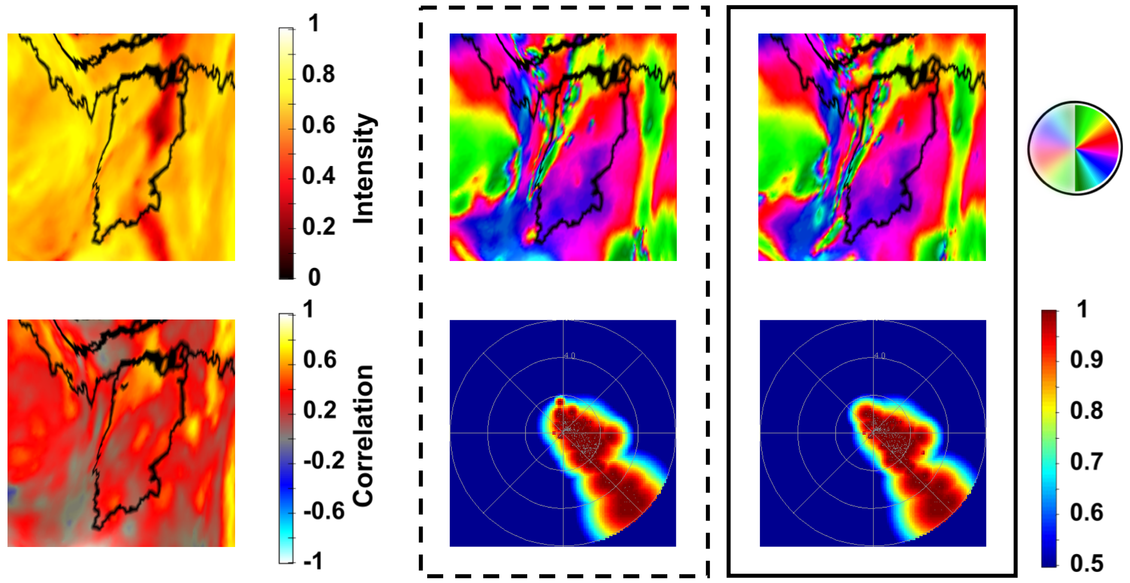

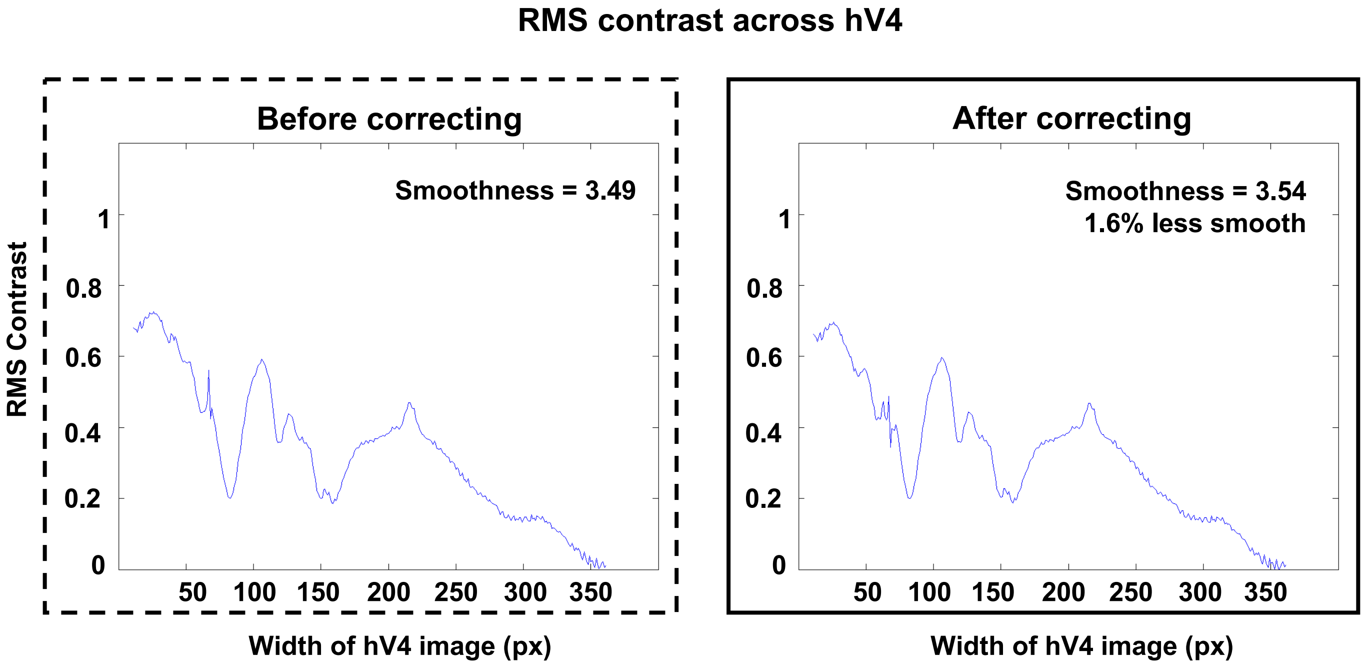

Subject 8 LH. Minimal changes are seen in hV4 after flipping inverted voxel time courses.

Sub-08 – Right Hemisphere

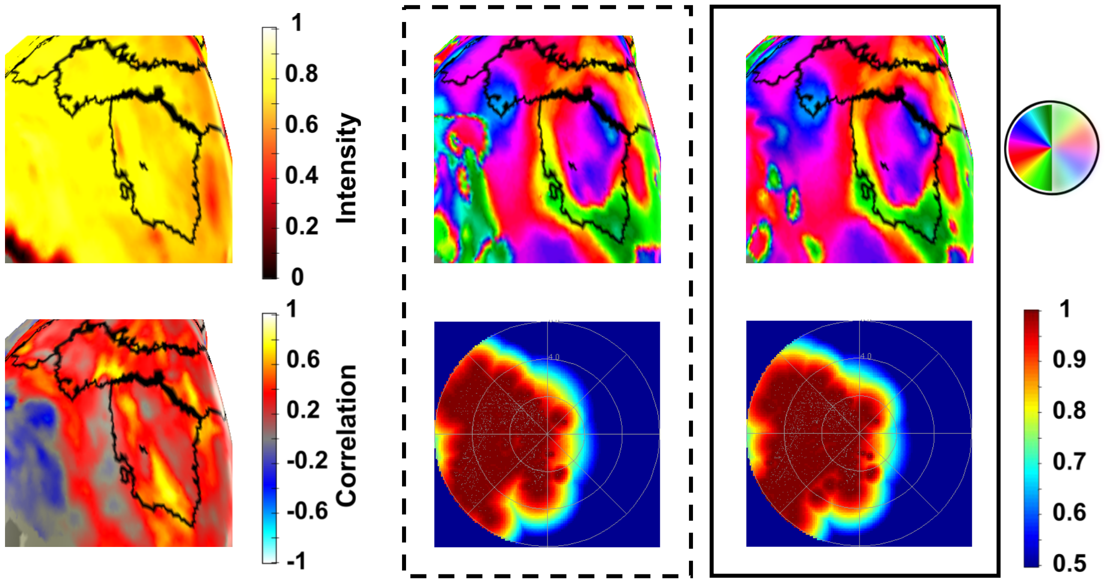

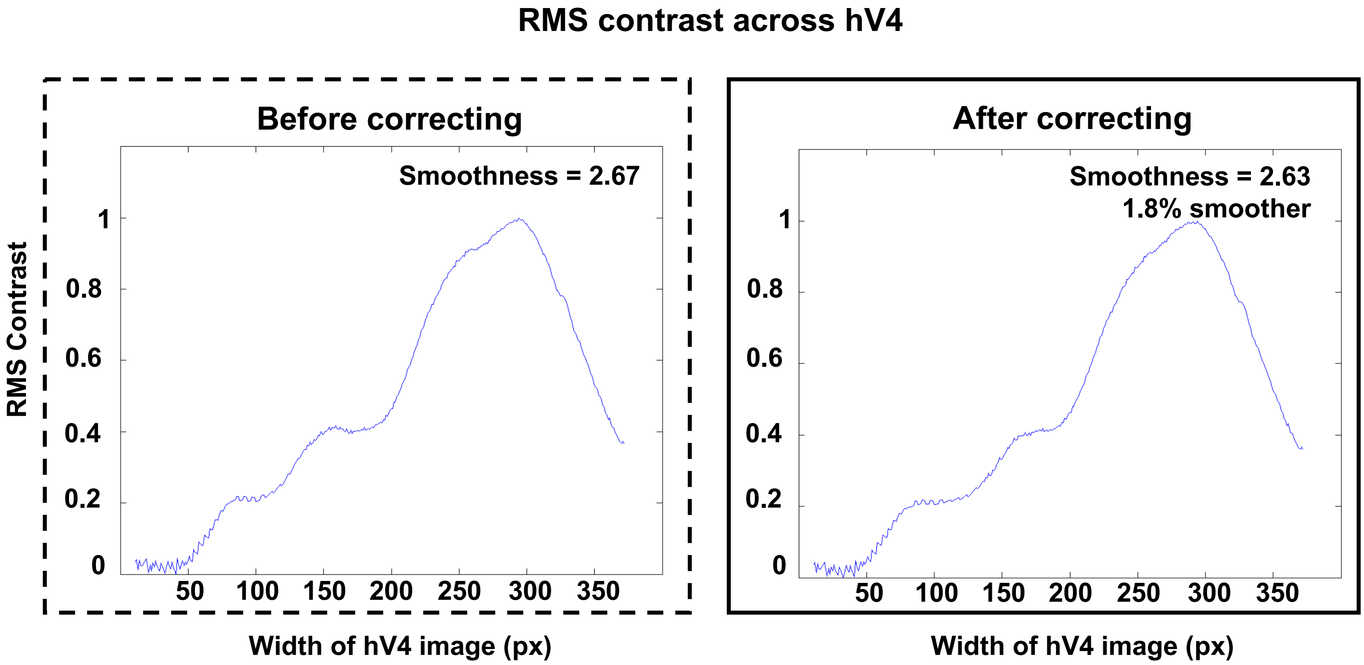

Subject 8 RH. Minimal changes are seen in hV4 after flipping inverted voxel time courses.

Sub-09 – Left Hemisphere

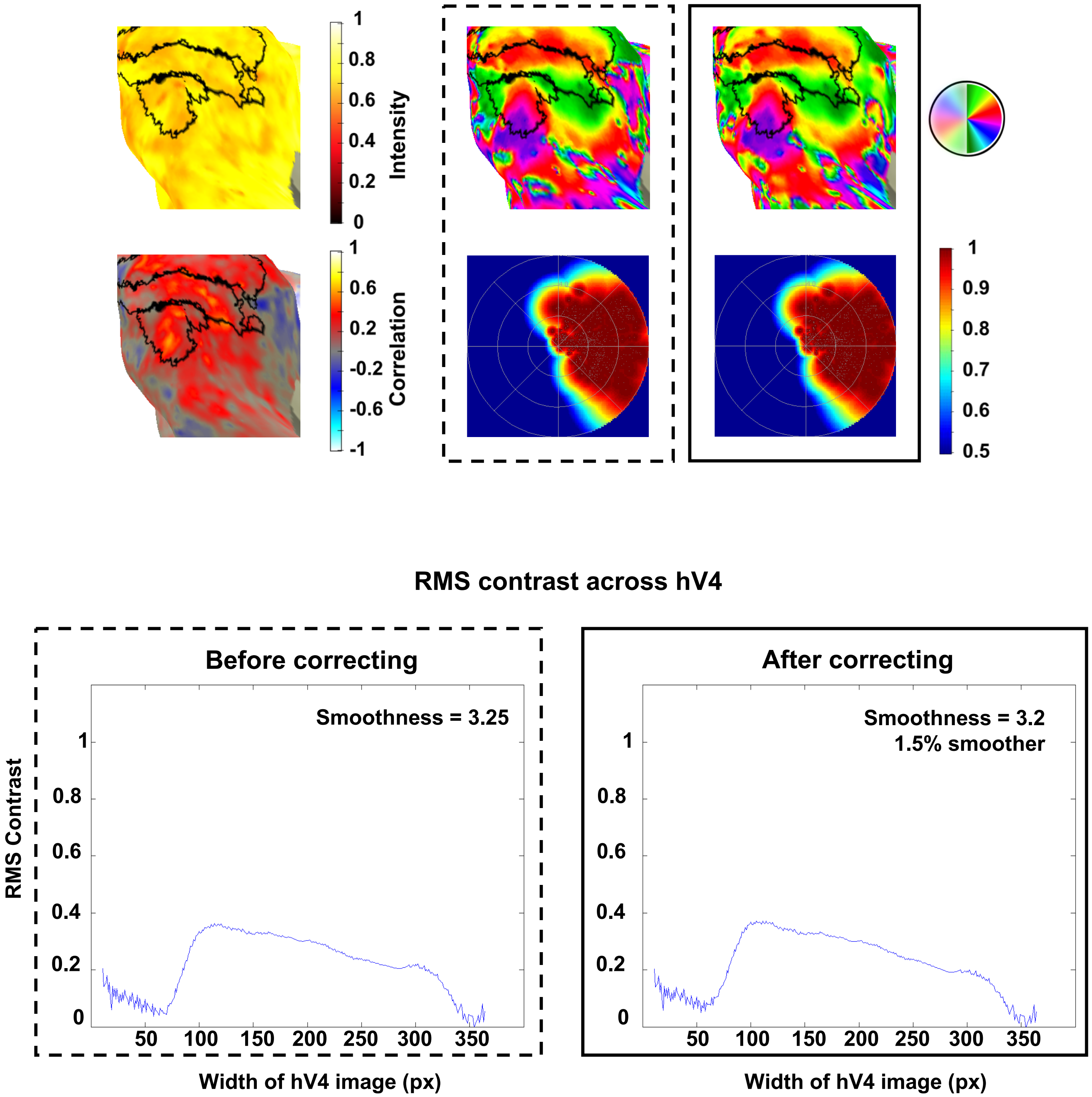

Subject 9 LH. Minimal changes are seen in hV4 after flipping inverted voxel time courses.

Sub-09 – Right Hemisphere

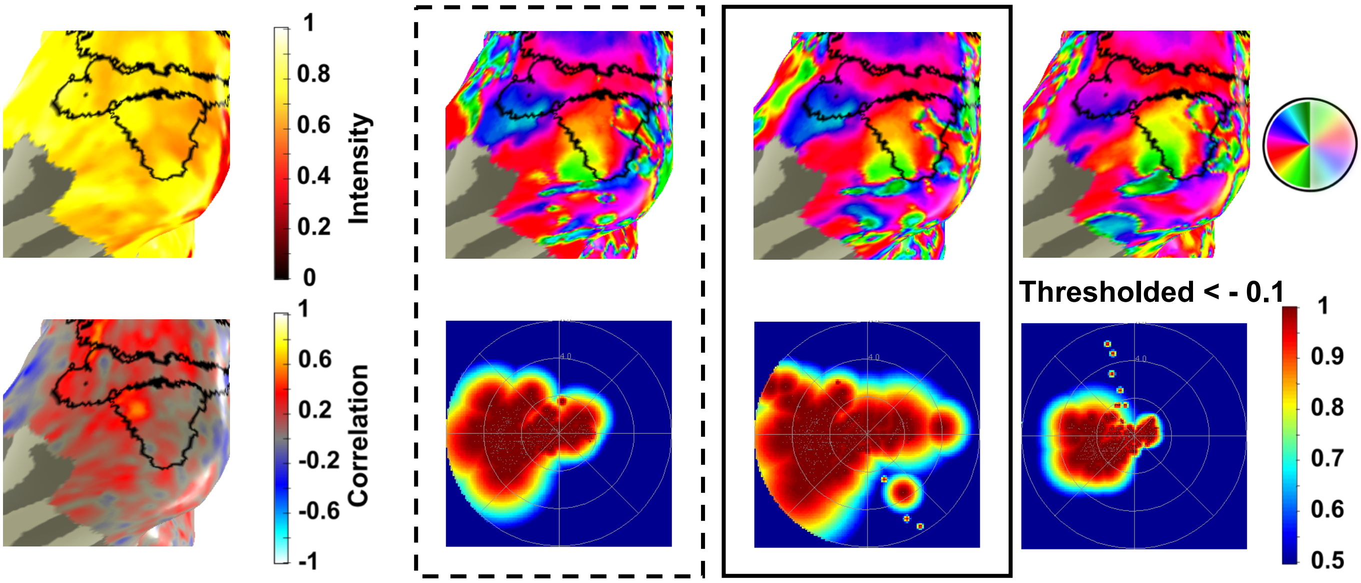

RMS contrast across hV4

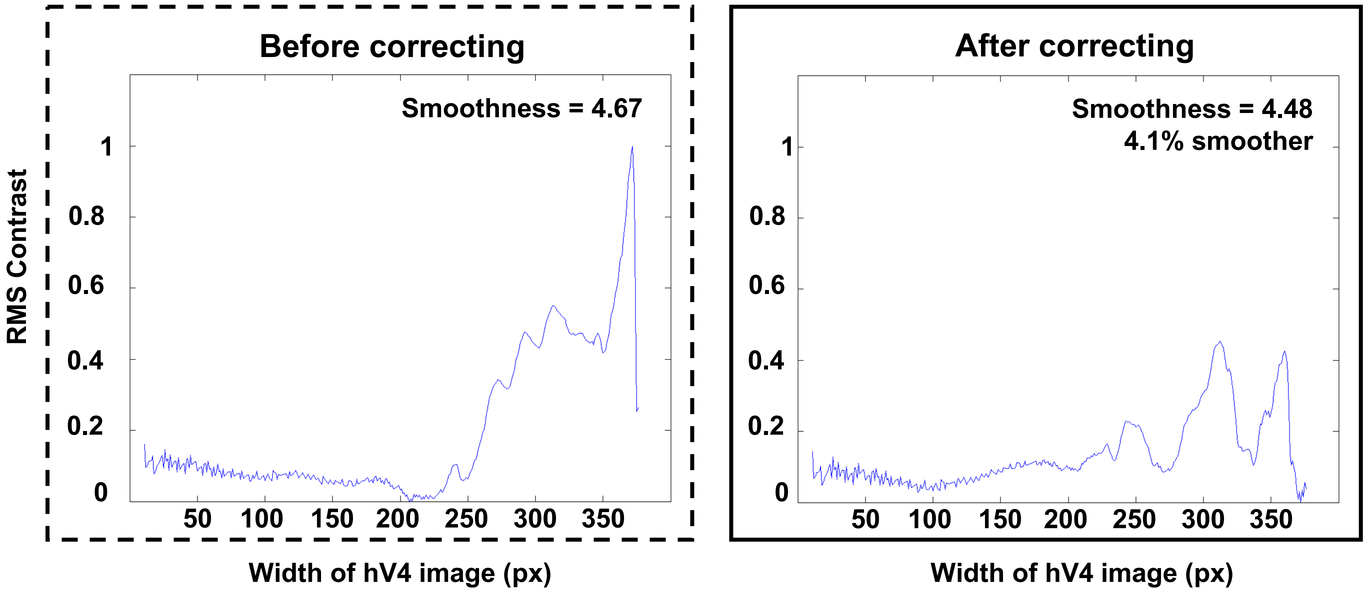

Subject 9 RH. Flipping inverted voxels results in more ipsilateral coverage post correction, which is somewhat improved by only correction inverted voxels with a correlation > -0.1 however, the resulting pRF plot is still less accurate after correction.

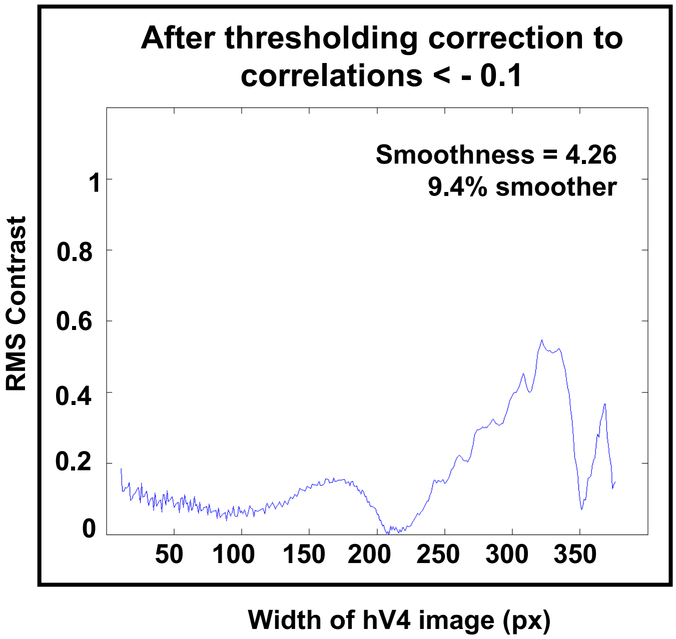

Sub-10 – Left Hemisphere

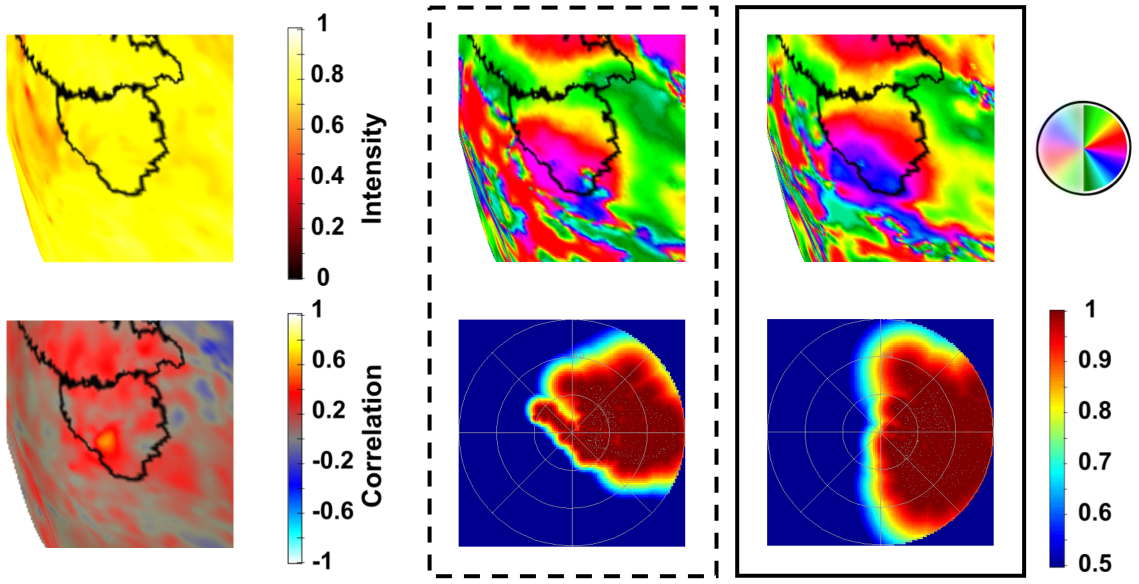

RMS contrast across hV4

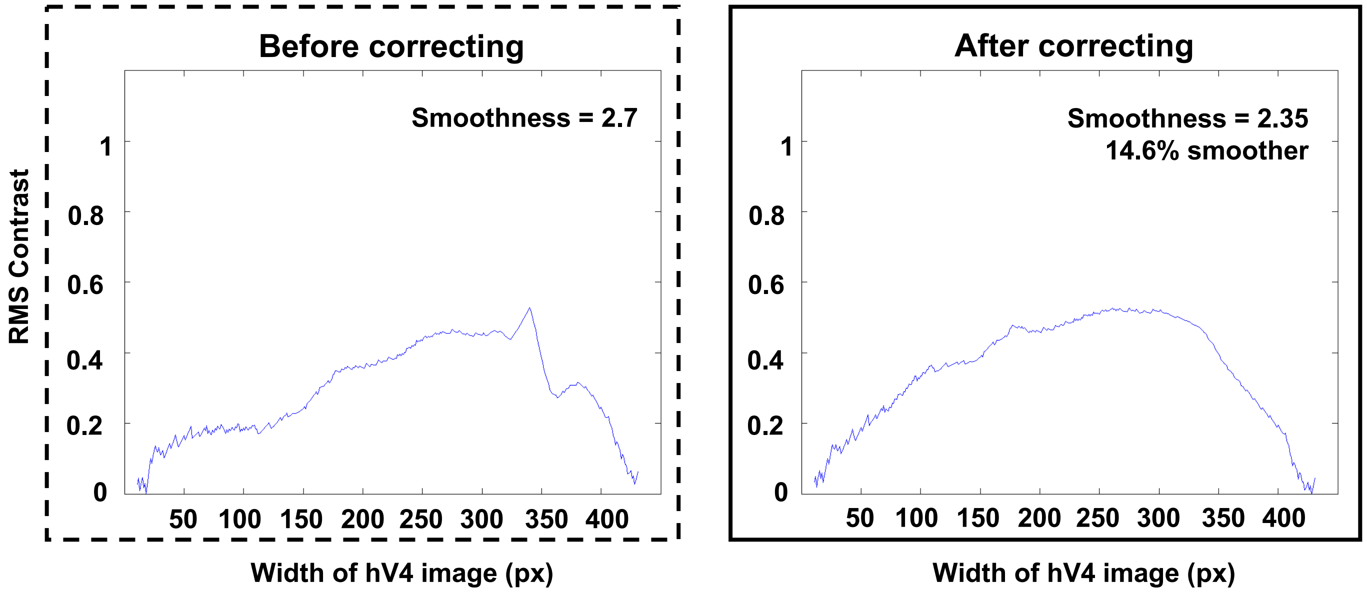

Subject 10 LH. A stronger lower visual quarterfield is represented in the polar angle map after flipping inverted voxels. The visual field coverage plot is dramatically improved by the inversion procedure, correcting the ipsilateral visual field representation and extending the lower quarterfield coverage to the lower vertical meridian. The corrected map is also much more smooth than the original.

Sub-10 – Right Hemisphere

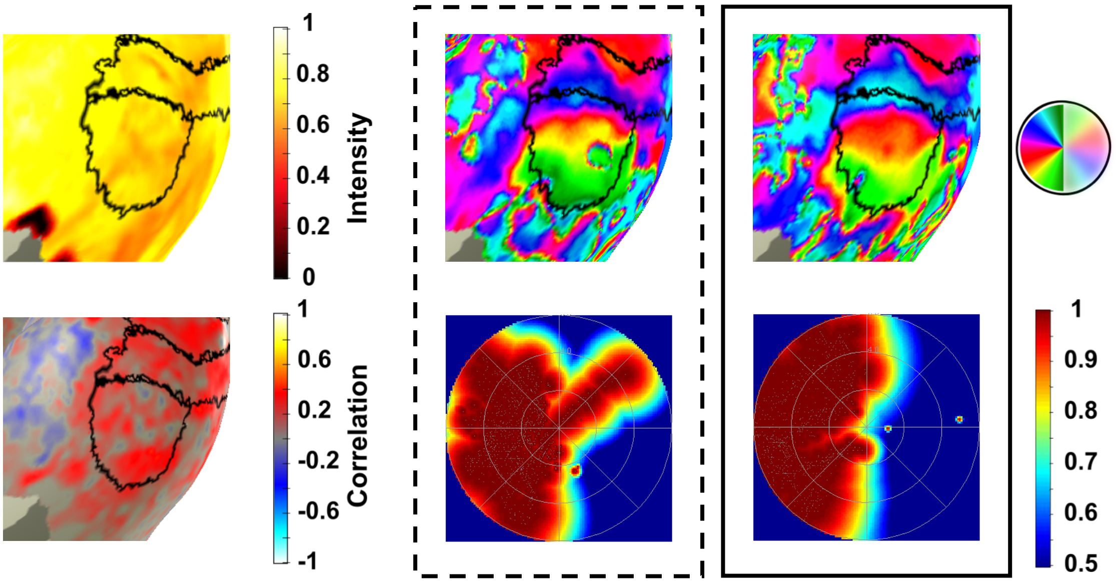

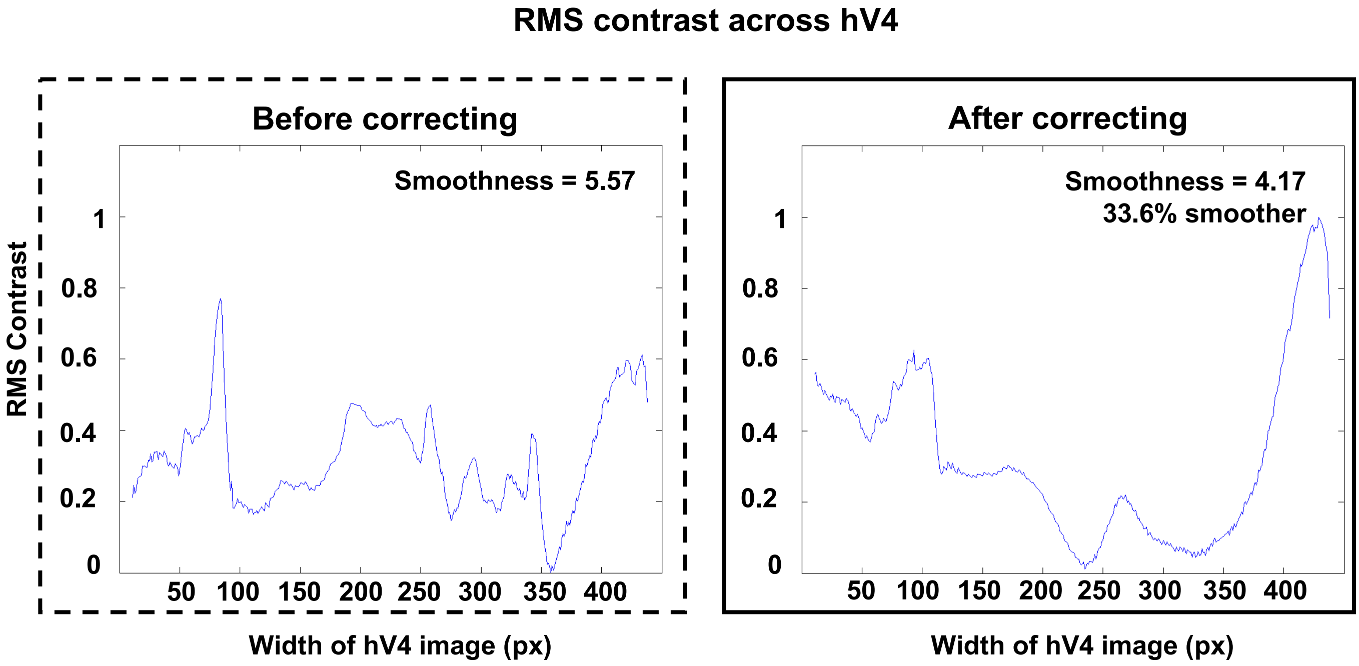

Subject 10 RH. A clearer upper vertical meridian is distinguished in the polar angle map after flipping inverted voxels, in addition to the map being smoother overall. The visual field coverage plot shows less ipsilateral visual field coverage.

Sub-11 – Left Hemisphere

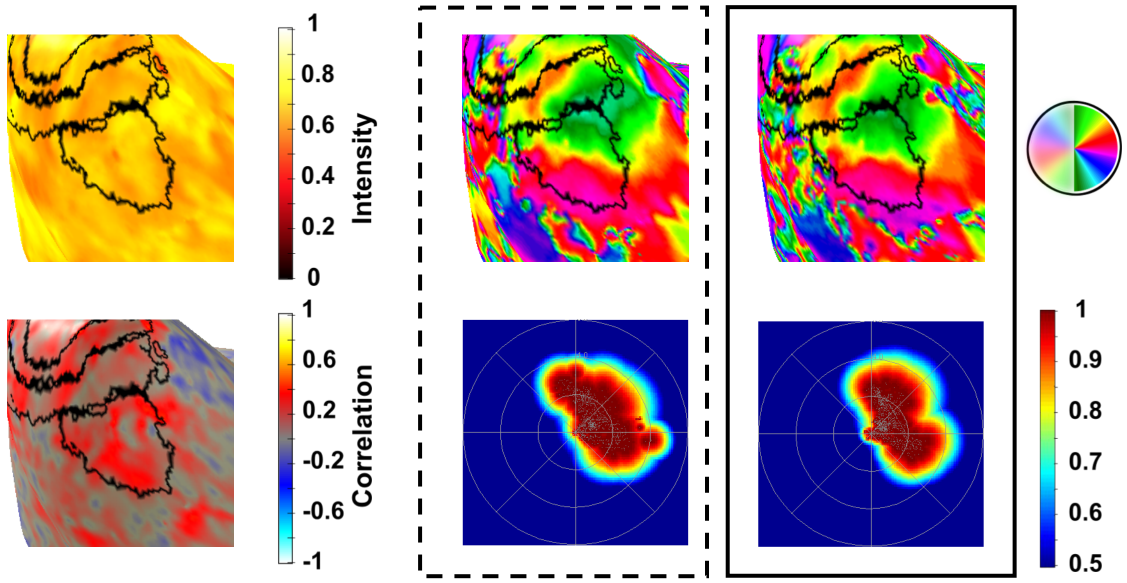

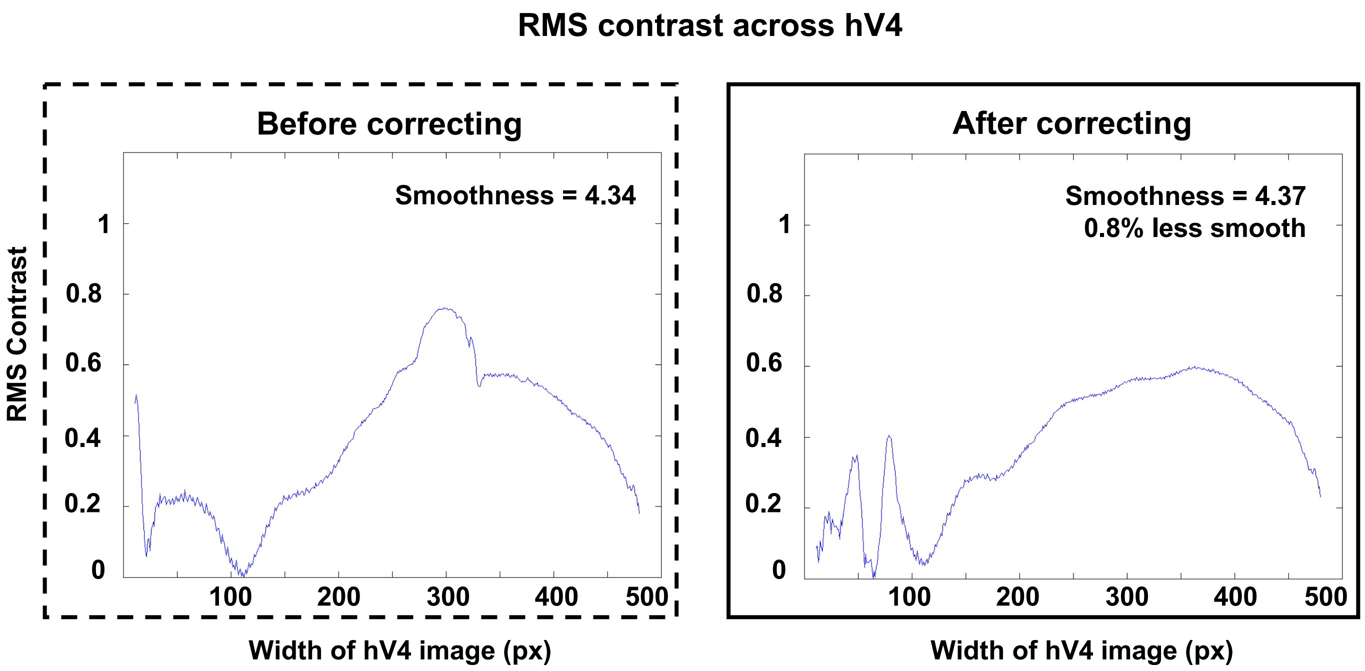

Subject 11 LH. Minimal changes are seen in hV4 after flipping inverted voxel time courses.

Sub-11 – Right Hemisphere

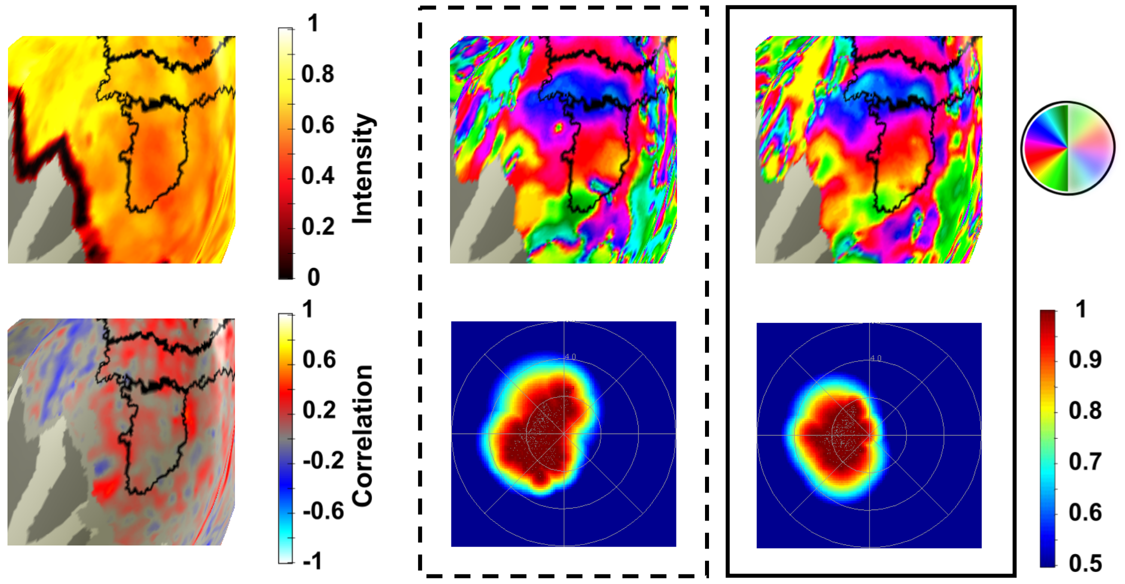

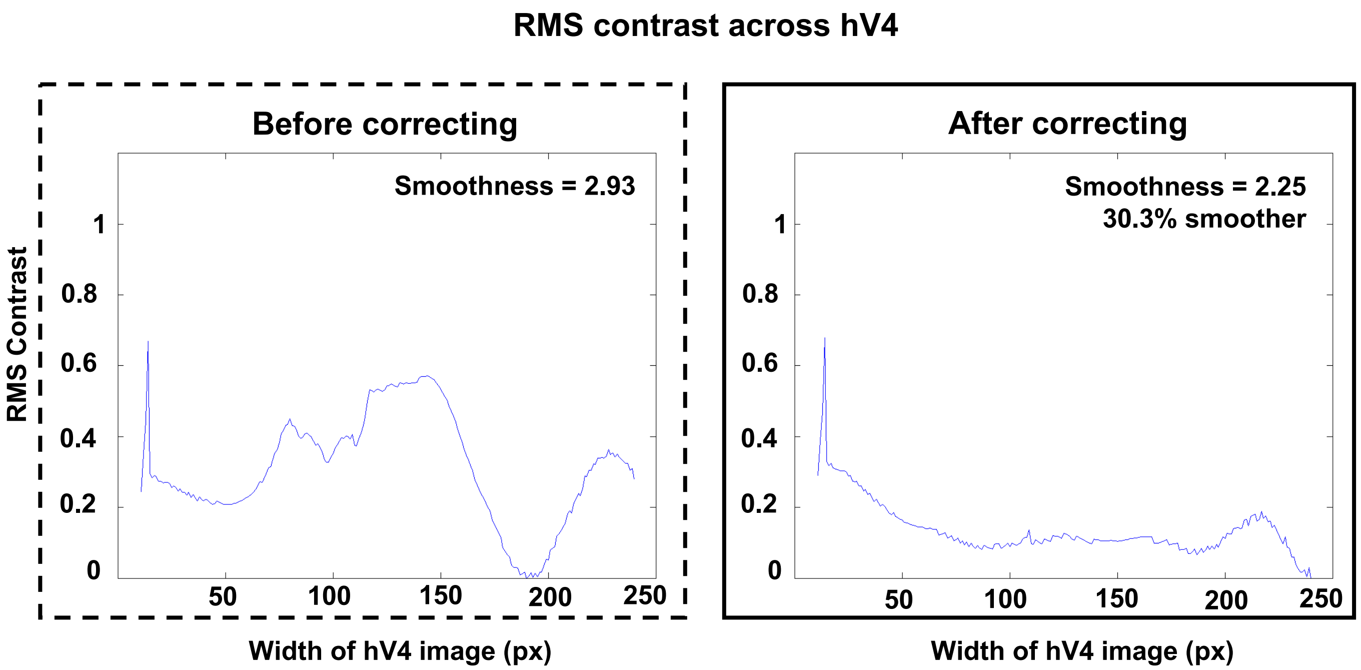

Subject 11 RH. A clearer upper vertical meridian is distinguishable in the polar angle map after flipping inverted voxels, as well as the lower boundary not being disturbed by an apparent upper quarterfield representation. This is reflected in the smoothness measure post correction, with an improvement of 30.3% in map smoothness.

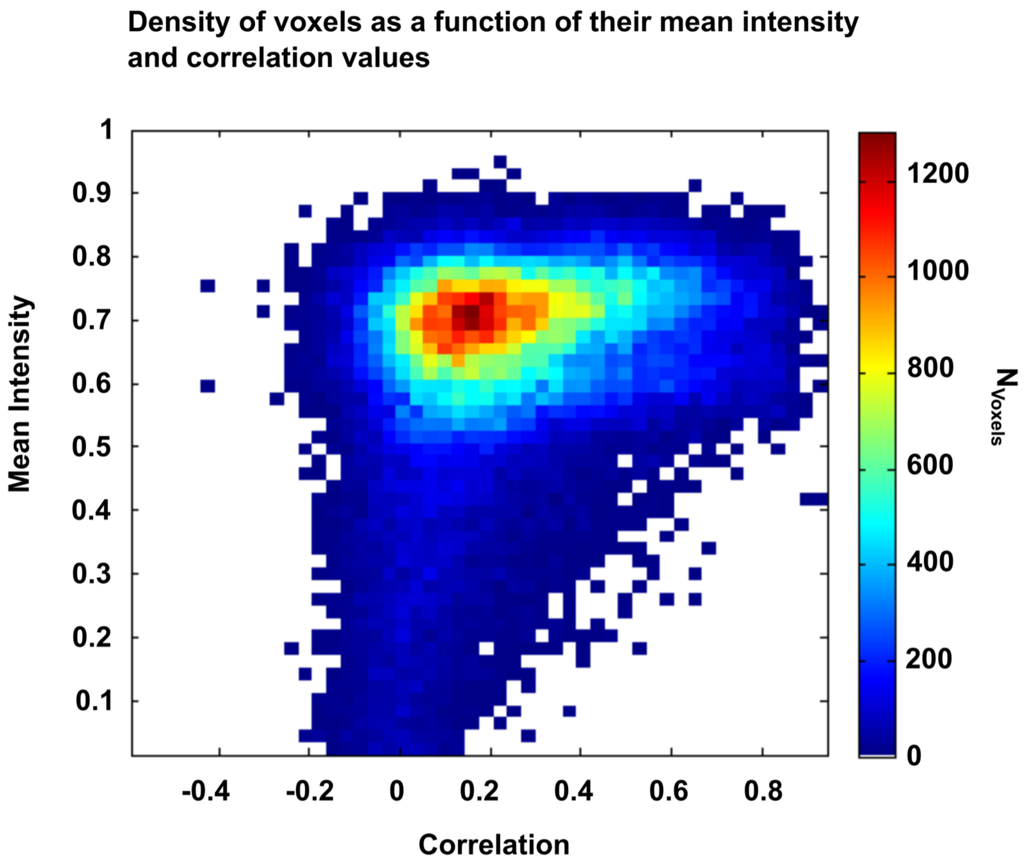

2D histogram: Voxels from combined V1—V4 left and right hemisphere ROIs from all subjects as a function of their mean intensity and correlation values. Voxels with a positive correlation tend to have stronger mean intensities. There is a moderate correlation between these values ($r = 0.25, p<0.001, N_{voxels} = 215\ 001$).

Table 1: Percentage of visual field covered in each hemifield quadrant - V1.

V1

	**Upper**	**Mid-upper**	**Mid-lower**	**Lower**
		**Left Hemisphere**		
sub-01	89.9	93.9	97.6	98.5
sub-02	88.4	88.0	95.6	98.6
sub-03	78.6	92.7	96.9	74.5
sub-04	95.8	97.4	97.8	97.6
sub-05	95.8	95.9	86.4	84.6
sub-06	87.1	90.8	98.6	98.6
sub-08	95.7	96.9	97.4	96.1
sub-09	85.6	95.3	98.6	98.6
sub-10	90.0	93.9	98.6	98.6
sub-11	89.8	85.0	84.8	47.5
**Average**	**89.7**	**93.0**	**95.2**	**89.3**
		**Right Hemisphere**		
sub-01	94.5	94.4	88.0	75.0
sub-02	89.5	94.6	95.8	97.4
sub-03	82.9	84.1	85.6	97.4
sub-04	78.1	90.3	91.5	96.1
sub-05	86.3	76.8	89.5	84.1
sub-06	83.9	90.6	95.8	90.3
sub-08	87.9	89.2	95.4	97.4
sub-09	94.6	94.7	95.0	94.3
sub-10	94.6	94.7	90.8	90.3
sub-11	64.0	86.8	95.8	97.4
**Average**	**85.6**	**89.6**	**92.3**	**92.0**

Table 2: Percentage of visual field covered in each hemifield quadrant - V2.

V2

	**Upper**	**Mid-upper**	**Mid-lower**	**Lower**
		**Left Hemisphere**		
sub-01	95.8	97.4	98.6	98.6
sub-02	78.9	97.4	93.0	87.8
sub-03	84.8	96.7	93.6	98.6
sub-04	95.6	94.4	97.0	96.1
sub-05	88.1	82.3	74.2	73.7
sub-06	95.8	93.5	98.3	97.8
sub-08	79.2	90.8	92.3	98.4
sub-09	74.5	97.4	98.6	98.6
sub-10	93.9	97.4	98.6	98.6
sub-11	95.7	97.4	94.2	83.3
**Average**	**88.2**	**94.5**	**93.8**	**93.1**
		**Right Hemisphere**		
sub-01	85.3	93.1	95.5	97.4
sub-02	94.7	94.7	95.8	97.4
sub-03	91.5	94.4	95.3	95.0
sub-04	80.2	93.0	86.5	97.4
sub-05	89.2	86.0	88.8	90.9
sub-06	74.9	94.7	94.4	97.4
sub-08	73.9	91.9	85.5	93.8
sub-09	94.7	94.7	95.8	97.2
sub-10	81.4	94.7	95.8	97.4
sub-11	93.3	94.7	95.8	94.9
**Average**	**85.9**	**93.2**	**92.9**	**95.9**

Table 3: Percentage of visual field covered in each hemifield quadrant - V3.

V3

	**Upper**	**Mid-upper**	**Mid-lower**	**Lower**
		**Left Hemisphere**		
sub-01	93.3	97.4	85.4	98.6
sub-02	95.8	97.4	93.4	98.5
sub-03	95.8	97.4	88.7	92.3
sub-04	95.8	97.0	90.3	68.3
sub-05	95.8	97.4	69.9	69.4
sub-06	93.3	97.4	94.3	68.9
sub-08	38.0	97.4	78.3	87.8
sub-09	95.8	97.4	98.6	98.6
sub-10	95.8	97.4	97.7	98.6
sub-11	95.8	94.2	97.5	92.0
**Average**	**89.5**	**97.1**	**89.4**	**87.3**
		**Right Hemisphere**		
sub-01	94.7	94.0	94.4	95.7
sub-02	81.6	94.7	95.8	97.4
sub-03	84.7	94.7	84.0	97.4
sub-04	83.4	91.2	72.8	97.4
sub-05	49.9	57.6	95.8	89.9
sub-06	81.5	94.7	95.8	97.4
sub-08	84.1	94.7	95.8	97.4
sub-09	89.9	94.7	95.8	97.4
sub-10	91.8	94.7	95.7	97.4
sub-11	86.3	94.7	72.5	37.0
**Average**	**82.8**	**90.6**	**89.8**	**90.5**

Table 4: Percentage of visual field covered in each hemifield quadrant - V4.

V4

	**Upper**	**Mid-upper**	**Mid-lower**	**Lower**
		**Left Hemisphere**		
sub-01	50.2	97.4	95.1	35.8
sub-02	92.0	96.7	98.6	48.8
sub-03	24.3	78.6	63.9	**19.1**
sub-04	**15.4**	44.8	56.9	**1.3**
sub-05	49.1	25.3	46.7	**7.8**
sub-06	38.6	55.8	98.0	**17.4**
sub-08	**5.8**	**13.7**	55.2	50.5
sub-09	64.0	97.4	98.6	40.1
sub-10	58.0	96.0	82.5	**11.0**
sub-11	35.9	40.3	44.6	**6.7**
**Average**	**43.3**	**64.6**	**74.0**	**23.8**
		**Right Hemisphere**		
sub-01	94.4	94.7	75.7	50.2
sub-02	25.4	26.8	92.1	50.3
sub-03	**8.1**	65.2	95.8	51.8
sub-04	64.3	78.4	58.5	92.0
sub-05	**14.0**	**16.3**	**15.8**	**11.7**
sub-06	80.4	84.3	95.8	67.6
sub-08	49.5	94.7	92.8	74.8
sub-09	21.6	74.8	85.9	28.3
sub-10	72.2	93.7	94.9	97.4
sub-11	27.8	27.5	36.3	24.4
**Average**	**45.8**	**65.6**	**74.4**	**54.8**

*Bold font in subject row indicates an incomplete hemifield quadrant.

Table 5: Percentage of visual field covered in each hemifield quadrant - V4 post correction.

V4

	Upper	Mid-upper	Mid-lower	Lower
		**Left Hemisphere**		
sub-01	47.5	97.4	98.0	42.2
sub-02	91.2	97.4	98.6	48.7
sub-03	24.8	77.4	61.8	**25.8**
sub-04	18.9	40.3	59.0	3.3
sub-05	49.3	25.3	46.7	**31.4**
sub-06	38.6	51.7	82.4	11.3
sub-08	4.8	13.7	53.2	48.9
sub-09	64.0	97.4	98.6	40.5
sub-10	70.1	97.4	98.6	**86.1**
sub-11	40.7	35.1	40.1	5.3
**Average**	**44.0**	**63.3**	**73.7**	**34.4**
		**Right Hemisphere**		
sub-01	92.4	94.7	82.2	44.7
sub-02	26.2	29.0	94.9	48.2
sub-03	8.1	62.6	95.8	51.8
sub-04	70.7	83.4	57.3	91.7
sub-05	14.0	16.3	15.8	11.7
sub-06	80.4	84.7	95.8	66.6
sub-08	46.6	93.8	92.7	73.5
sub-09	23.2	87.4	95.6	57.3
sub-10	94.6	94.7	95.6	91.2
sub-11	**14.5**	31.4	33.0	22.1
**Average**	**47.1**	**67.8**	**75.9**	**55.9**

*Bold font in subject row indicates a change from a complete/incomplete to an incomplete/complete hemifield quadrant.
